# Hypervalent iodine-catalyzed amide and alkene coupling enabled by lithium salt activation

**DOI:** 10.3762/bjoc.20.122

**Published:** 2024-06-24

**Authors:** Akanksha Chhikara, Fan Wu, Navdeep Kaur, Prabagar Baskaran, Alex M Nguyen, Zhichang Yin, Anthony H Pham, Wei Li

**Affiliations:** 1 Department of Chemistry and Biochemistry, School of Green Chemistry and Engineering, The University of Toledo, 2801 West Bancroft Street, Toledo, Ohio 43606, United States

**Keywords:** amide coupling, hypervalent iodine catalysis, lithium salt activation, olefin oxyamination, oxazoline

## Abstract

Hypervalent iodine catalysis has been widely utilized in olefin functionalization reactions. Intermolecularly, the regioselective addition of two distinct nucleophiles across the olefin is a challenging process in hypervalent iodine catalysis. We introduce here a unique strategy using simple lithium salts for hypervalent iodine catalyst activation. The activated hypervalent iodine catalyst allows the intermolecular coupling of soft nucleophiles such as amides onto electronically activated olefins with high regioselectivity.

## Introduction

**Scheme 1:**
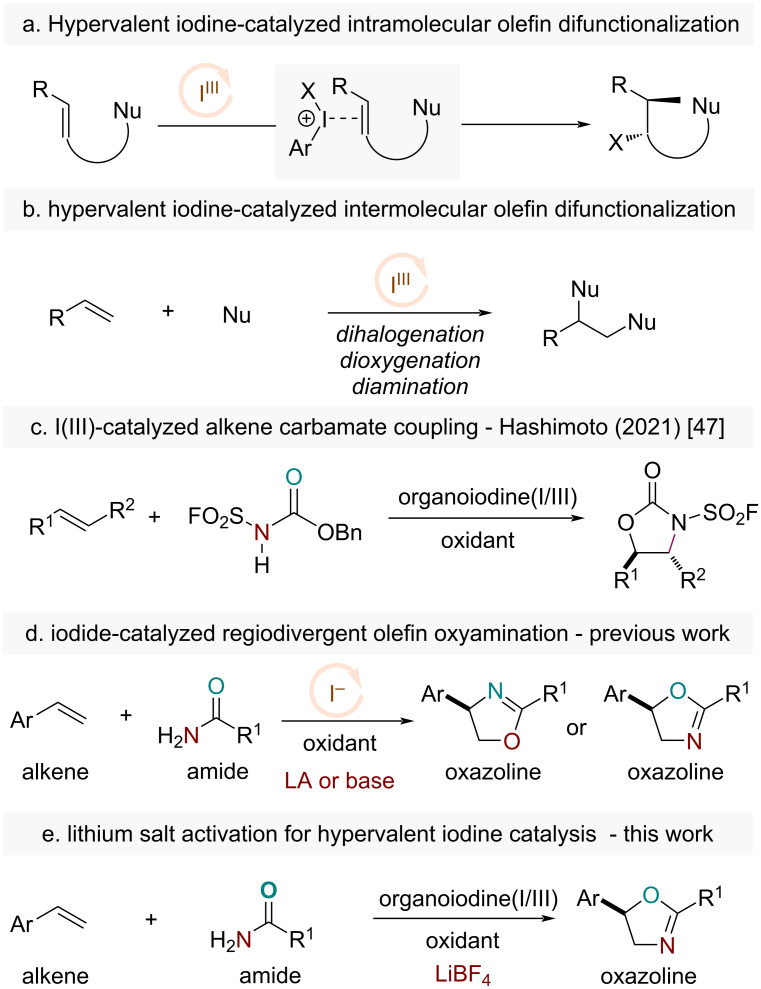
Hypervalent iodine-catalyzed olefin difunctionalizations background.

Hypervalent iodine(III) reagents, also known as λ^3^–iodanes, have been well established and used in organic synthesis for the past decades [1–5]. The pioneering works of Fuchigami and Fugita, Ochiai, Kita, and later the development of chiral hypervalent iodines by Wirth, Kita, Ishihara, Muñiz, and many others, have firmly established these reagents as useful catalysts for a wide variety of chemical transformations [6–17]. A number of features, including low toxicity, high stability, ease of handling, and versatile reactivity, etc. render these catalysts highly attractive for adoption in organic synthesis. In particular, the field of olefin difunctionalization, known for its rapid assembly of molecular complexity, has been a fertile ground for innovation for hypervalent iodine catalysis, which often involves the catalytic use of an iodoarene with stoichiometric oxidants such as MCPBA, Selectfluor, etc. [18–20]. Earlier and recent hypervalent iodine-catalyzed olefin halofunctionalizations by several groups have predicated on the use of intramolecular olefin substrates tethered with a nucleophile to avoid the lack of regiochemical additions (Scheme 1a) [21–28]. Intermolecular hypervalent iodine-catalyzed olefin difunctionalizations have been realized for olefin dihalogenation, dioxygenation and diamination reactions, where often the same type of nucleophiles were incorporated (Scheme 1b) [29–40]. Intermolecular hypervalent iodine catalysis with the regioselective additions of two distinct nucleophilic functionalities across an olefin, however, remains challenging with limited solutions [41–46]. Notably, an interesting work by Hashimoto has recently enabled the intermolecular addition of *N*-(fluorosulfonyl)-protected carbamates as oxyamination reagents across a variety of olefin structures [47]. This work engages the hypervalent iodine catalyst in an anionic ligand exchange with the substrate, which then partitions into an ion pair suitable for olefin activation, followed by the addition of the bifunctional anionic carbamate (Scheme 1c).

Our hypothesis here aims to directly access the reactivity of the cationic hypervalent iodine catalyst through an initial activation first, which we reason will then enable soft nucleophiles such as unadorned amides to readily participate in the ensuing olefin addition. In this regard, we wondered if the hypervalent iodine with difluoro ligands could undergo salt metathesis with lithium salts such as LiBF_4_ or LiPF_6_ to afford the more reactive cationic hypervalent iodine catalyst. The cationic hypervalent iodine catalyst could then activate the olefin to allow the addition of bifunctional nucleophiles such as an amide to achieve an overall olefin oxyamination process. We have previously reported a series of iodide-catalyzed processes, in which the electrophilicity of the halogen source could be modulated to render different classes of nucleophiles for additions onto olefins in various olefin difunctionalization reactions [48–52]. In particular, we demonstrated that addition of either a Lewis acid or a base could activate amides to couple with alkenes regioselectively to furnish their respective oxazoline regioisomer (Scheme 1d). Herein, we report that lithium salts such as LiBF_4_ or LiPF_6_, which are often used in lithium-ion batteries, can be used to activate hypervalent iodine catalysts to enable olefin oxyamination reactions with simple bifunctional amide nucleophiles (Scheme 1e).

## Results and Discussion

Our studies here focused on the development of hypervalent iodine-catalyzed amide and alkene coupling reaction [53–55]. In this case, we started with styrene (**1**) and benzamide (**2**) as the standard substrates. Using iodotoluene **A** as the hypervalent iodine catalyst precursor, Selectfluor as the oxidant, and LiBF_4_ as the lithium salt for hypervalent iodine activation, we were gratified to observe the formation of the desired oxazoline **3** in 59% yield as the major regioisomer in nitromethane (MeNO_2_) solvent (Table 1, entry 1). To further improve the reaction efficiency, we screened several additional parameters including solvents and concentration. In these cases, we found that while both acetonitrile and MeNO_2_ (0.25 M) were suitable solvents, other solvents in general afforded no product formation (Table 1, entries 2–6). Lower catalyst loading and longer reaction time did not improve the overall reaction efficiency (Table 1, entries 6–8). Furthermore, we evaluated several salt additives containing different counterions, and found that LiBF_4_ was the optimal additive (Table 1, entries 7, 9, and 10). The optimal conditions were shown in entry 7 in Table 1, resulting in the formation of the desired oxazoline product **3** in 61% isolated yield. Control reactions in the absence of either the precatalyst or oxidant afforded no product formation (Table 1, entries 11 and 12). The control reaction in the absence of the lithium salt only afforded 8% of the oxazoline product **3** (Table 1, entry 13). These reactions validated the critical roles of each individual component to achieve an efficient reaction.

**Table 1:** Amide and alkene reaction optimization studies.

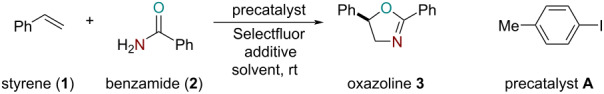					

entry^a^	precatalyst (mol %)	solvent (M)	additive (mol %)	yield (%)^c^	rr

1	**A** (20)	MeNO_2_ (0.5)	LiBF_4_ (100)	59	>95:5
2	**A** (20)	MeCN (0.5)	LiBF_4_ (100)	41	94:6
3	**A** (20)	MeOH (0.5)	LiBF_4_ (100)	0	–
4	**A** (20)	DMF (0.5)	LiBF_4_ (100)	0	–
5	**A** (20)	MeNO_2_ (0.3)	LiBF_4_ (100)	62	>95:5
6^b^	**A** (20)	MeNO_2_ (0.25)	LiBF_4_ (100)	64	>95:5
7	**A** (20)	MeNO_2_ (0.25)	LiBF_4_ (100)	65 (61)	>95:5
8	**A** (15)	MeNO_2_ (0.5)	LiBF_4_ (100)	55	>95:5
9	**A** (20)	MeNO_2_ (0.5)	LiPF_6_ (100)	56	>95:5
10	**A** (20)	MeNO_2_ (0.5)	AgBF_4_ (100)	12	>95:5
11	–	MeNO_2_ (0.5)	LiBF_4_ (100)	0	–
12^d^	**A** (20)	MeNO_2_ (0.5)	LiBF_4_ (100)	0	–
13	**A** (20)	MeNO_2_ (0.5)	–	8	>95:5

^a^Optimized conditions: styrene (**1**, 0.25 mmol), iodotoluene **A** (20 mol %), LiBF_4_ (100 mol %), Selectfluor (150 mol %), benzamide (**2**, 400 mol %), MeNO_2_ (0.25 M), rt, 16 h. Yields were determined by crude ^1^H NMR using 1,3-benzodioxole as the internal standard. ^b^Reaction time is 24 hours. ^c^The yield in parenthesis is isolated yield. ^d^No Selectfluor added.

**Figure 1:**
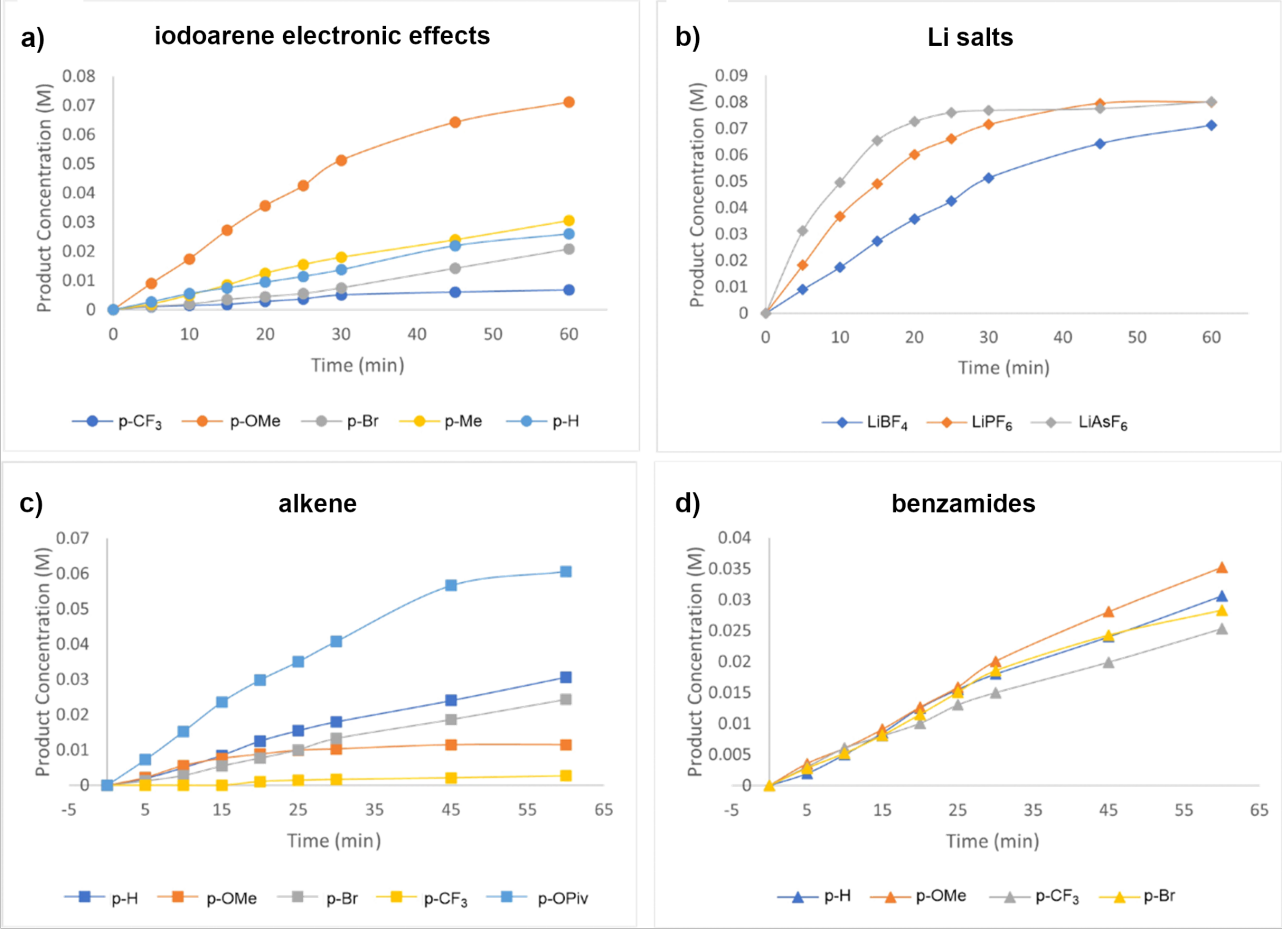
Time studies of the amide and alkene coupling. a) Iodoarene time studies: styrene (**1**), para-substituted iodoarenes, LiBF_4_, and benzamide (**2**). b) Li salt time studies: styrene (**1**), iodoanisole, Li salts, and benzamide (**2**). c) Alkene time studies: para-substituted styrenes, iodotoluene **A**, LiBF_4_, and benzamide (**2**). d) Benzamide time studies: styrene (**1**), iodotoluene **A**, LiBF_4_, and para-substituted benzamides.

To understand this coupling reaction better, we have also performed time studies to elucidate the effects of several key features in this reaction. First, we studied the iodoarene catalyst precursor and the lithium salt in terms of their effects on the overall reaction rate. In this case, we observed that the overall reaction proceeded faster with the more electron-rich iodoarene catalysts than electron-poor ones. Qualitatively, the electron-rich iodoarene catalysts are likely to be worse at activating the olefins than the electron-deficient hypervalent iodine catalysts. Therefore, the faster rate with the electron-rich catalyst precursor is because the electron-rich iodoarene catalyst precursors are more easily oxidized to the hypervalent iodine catalyst with difluoro ligands. Interestingly, the use of different lithium salts also impacted the overall reaction rate, with the reaction using the less coordinating LiAsF_6_ salt proceeding faster than LiPF_6_ and LiBF_4_. This time study suggested that the hypervalent iodine precatalyst with the less coordinating counterion is more reactive to activate the olefin. We also conducted kinetic studies on how the olefin and amide structures impacted the overall reaction rate. The more electron-rich olefins generally proceeded faster than the electron-poor ones, suggesting that a significant positive charge was likely built up on the olefin prior to the nucleophilic addition. On the other hand, the electronic nature of the para-substituted benzamides had little impact on the overall reaction rate as both electron-rich and electron-deficient benzamides proceeded with similar kinetic profiles. All the kinetic plots are shown in Figure 1.

**Figure 2:**
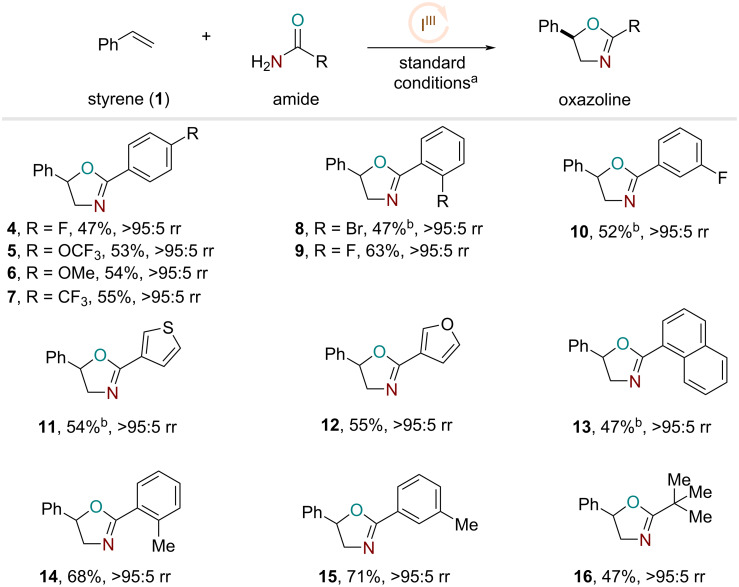
Amide substrate scope studies. a) Standard conditions: styrene (0.25 mmol), iodotoluene (20 mol %), LiBF_4_ (100 mol %), Selectfluor (150 mol %), amide (400 mol %), MeNO_2_ (0.25 M), rt, 16 h. b) Iodoanisole (20 mol %).

With the optimized conditions and kinetic information in hand, we turned our attention to the amide substrate scope. In this case, both electron-rich and -deficient benzamides proceeded to the desired products in reasonable yields and high regioselectivities (Figure 2, products **4**–**7**). Concurring with our kinetic data, the electronic nature of the amide bears little impact on the overall reaction rate and in this case, on the final yields as well. Similarly, ortho- and meta-substituted benzamides with halogen functionalities could also generate the desired oxazoline products with reasonable yields (Figure 2, products **8**–**10**). Heteroaromatic amides could also furnish the oxazolines **11** and **12** with good efficiency. Naphthaleneamide also generated the desired product **13**, albeit with slightly lower efficiency. Interestingly, *o*- and *m*-methyl-substituted benzamides provided a significant yield boost to provide the oxazoline structures **14** and **15**. Finally, sterically encumbered tertiary amides participated in the reaction to afford the respective regioisomeric product **16**.

**Figure 3:**
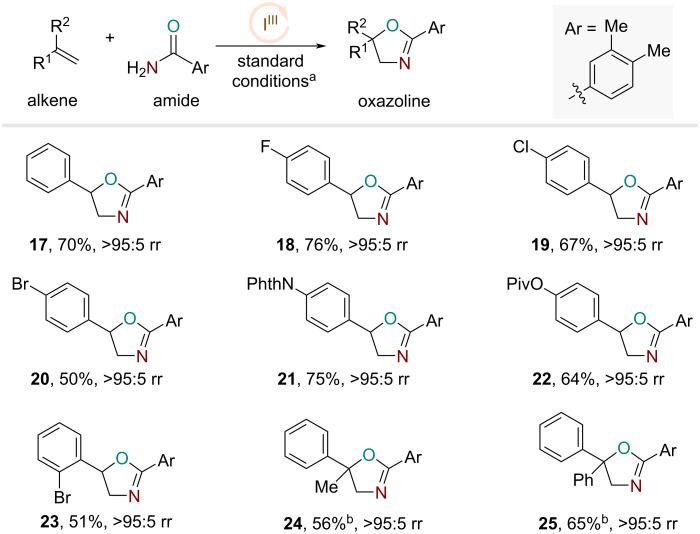
Alkene substrate scope studies. a) Standard conditions: alkene (0.25 mmol), iodotoluene (20 mol %), LiBF_4_ (100 mol %), Selectfluor (150 mol %), 3,4-dimethylbenzamide (400 mol %), MeNO_2_ (0.25 M), rt, 16 h. b) Iodoanisole (20 mol %), MeCN (0.25 M).

Encouraged by these results, we then turned our attention to explore the extent of alkene substrate scope using 3,4-dimethylbenzamide, which afforded the oxazoline product **17** in 70% yield (Figure 3). Based on this optimal amide structure, we examined various electronically activated olefins under the optimal reaction conditions. A number of styrenyl derivatives with para-substituted halogens, ester, and phthalimide proceeded smoothly with good yields and excellent regioselectivities to access the oxazoline products as single regioisomers (Figure 3, products **18**–**22**). The *o*-bromo-substituted styrene also afforded the corresponding product **23**. Furthermore, 1,1-di-substituted α-methylstyrene and α-phenylstyrene produced the respective oxazoline products with high regioselectivity and reasonable yields using iodoanisole as the catalyst precursor (Figure 3, products **24** and **25**).

**Figure 4:**
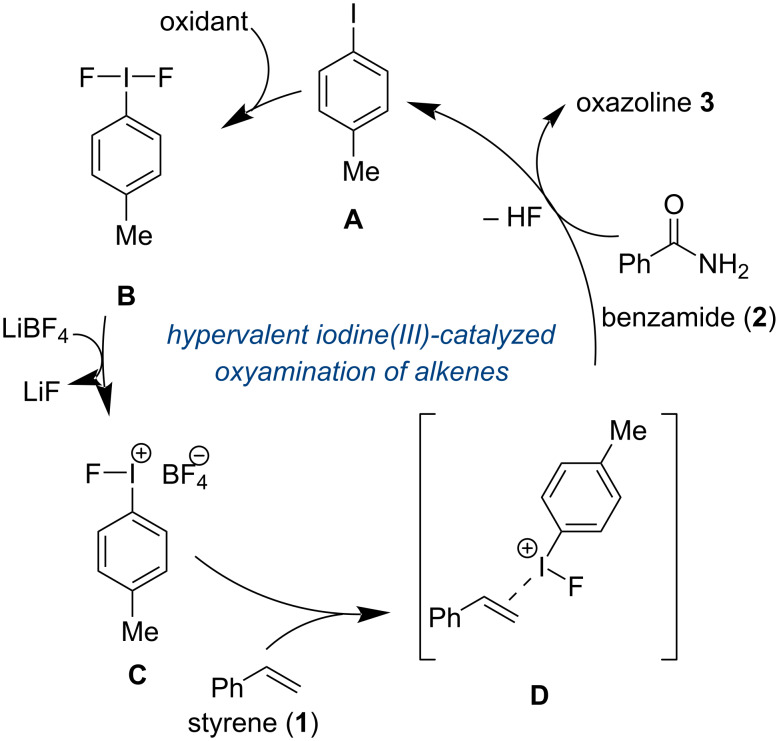
Proposed catalytic cycle for the hypervalent iodine-catalyzed amide and alkene coupling.

The proposed catalytic cycle (Figure 4) begins with iodotoluene **A** which is oxidized by Selectfluor salt into the difluorinated iodotoluene **B**. Then, LiBF_4_ can perform a salt metathesis with **B** to produce LiF along with the active hypervalent iodoarene catalyst **C**. The activated hypervalent iodine catalyst **C** can coordinate to the alkene to form complex **D.** The nucleophilic oxygen of the amide will attack in the internal position and subsequent cyclization will furnish the desired oxazoline.

## Conclusion

We have developed a hypervalent iodine-catalyzed amide and alkene coupling reaction. This reaction protocol furnished useful oxazoline products and introduced the use of lithium salts to activate hypervalent iodine catalysts. This strategy rendered the participation of simple and unadorned amides as bifunctional nucleophiles to achieve olefin oxyamination reactions. Time studies of these reactions further unveiled interesting mechanistic features that will be useful for our future catalysis development and asymmetric reaction designs.

## Supporting Information

File 1 Spectral characterization of the products and kinetic studies.

## Data Availability

The data that supports the findings of this study is available from the corresponding author upon reasonable request.
