# Supplementary material for: Hypervalent iodine-catalyzed amide and alkene coupling enabled by lithium salt activation
Source: Beilstein J Org Chem. 2024 Jun 24;20:1405–11. doi: 10.3762/bjoc.20.122 (PMC11216091; doi:10.3762/bjoc.20.122)
Supplement: File 1 — Spectral characterization of the products and kinetic studies. [file Beilstein_J_Org_Chem-20-1405-s001.pdf]

BEILSTEIN JOURNAL OF ORGANIC CHEMISTRY

Supporting Information

for

Hypervalent iodine-catalyzed amide and alkene coupling enabled by lithium salt activation

Akanksha Chhikara, Fan Wu, Navdeep Kaur, Prabagar Baskaran, Alex M. Nguyen, Zhichang Yin, Anthony H. Pham and Wei Li

Beilstein J. Org. Chem. **2024,** *20,* 1405–1411. doi:10.3762/bjoc.20.122

Spectral characterization of the products and kinetic studies

License and Terms: This is a supporting information file under the terms of the Creative Commons Attribution License (https://creativecommons.org/licenses/by/4.0). Please note that the reuse, redistribution and reproduction in particular requires that the author(s) and source are credited and that individual graphics may be subject to special legal provisions.
The license is subject to the *Beilstein Journal of Organic Chemistry* terms and conditions: (https://www.beilstein-journals.org/bjoc/terms)

Table of contents

1. General information S2

2. Experimental procedures S3

3.Spectral characterization of the products S3–S17

4. Kinetics data S18–S25

5. References S26

6. Spectral data S27–S54

General information

Commercial reagents and solvents were purchased from Sigma Aldrich, Oakwood Chemicals, Alfa Aesar, Matrix Scientific, Acros Organic and were used as received. Amides were recrystallized before use. Organic solutions were concentrated under reduced pressure on an IKA rotary evaporator using an acetone–dry ice bath. Chromatographic purification of products was accomplished using flash chromatography on 230–400 mesh silica gel. Thin-layer chromatography (TLC) was performed on Analtech 250 mm silica gel HLF UV-250 plates. Visualization of the developed plates was performed by fluorescent quenching and potassium permanganate. ^{1}H and ^{13}C NMR spectra were recorded on a Bruker instrument (600 and 150 MHz) or INOVA 600 (600 and 150 MHz) and are internally referenced to residual protio solvent signals (for $CDCl_3$, 7.26 and 77.0 ppm, respectively). ^{19}F NMR spectra were recorded on a Varian VXRS 400 (375 MHz). Data for ^{1}H NMR are reported as follows: chemicals shift (ppm), multiplicity (s = singlet, d = doublet, t = triplet, q = quartet, h = heptet, m = multiplet, br = broad), integration, coupling constant (Hz). ^{13}C and ^{19}F NMR spectra were recorded as chemical shifts in ppm and multiplicity where appropriate. IR spectra were recorded on a PerkinElmer FT-IR spectrophotometer and reported in terms of wavenumber of absorption (cm^{-1}). High resolution mass spectra were obtained on a Waters Synapt High-Definition Mass Spectrometer (HDMS) by electrospray ionization at the University of Toledo, OH, USA and a Maxis Ultra High-resolution ESI LC/MS at the University of Wisconsin-Madison, WI, USA.

General procedure for oxazoline synthesis

To an 8 mL vial equipped with a stir bar was added catalyst (11 mg, 0.05 mmol), benzamide (1.0 mmol), $LiBF_4$ (23 mg, 0.25 mmol) and F-TEDA-BF_4 (132 mg, 0.375 mmol). Then, $MeNO_2$ (2.0 mL) was added via syringe, followed by the alkene (0.25 mmol). The reaction mixture was then stirred for 16 h at room temperature. The reaction mixture was diluted with EtOAc (3 mL) and aqueous Na_2CO_3 solution (1 M, 2 mL) was then added. The organic layer was separated, and the aqueous layer was extracted with EtOAc (2 × 2 mL). The combined organic layer was dried over Na_2SO_4 and concentrated under reduced pressure to give the crude product, which was purified by column chromatography on silica gel to afford the pure product.

Spectral characterization of the products

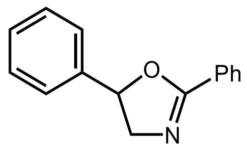

2,5-Diphenyl-4,5-dihydrooxazole (3): This compound was prepared according to the General Procedure, using styrene (28 μL, 0.25 mmol) and benzamide (121 mg, 1.0 mmol). After purification by column chromatography SiO_2 (10% to 20% EtOAc in hexanes), the title compound was isolated as a brown oil (34 mg, 61% yield). Spectral data matched with previously reported literature.[1]

^{1}H NMR (600 MHz, $CDCl_3$): 8.05 (d, J = 7.6 Hz, 2 H), 7.53 (t, J = 7.3 Hz, 1 H), 7.46 (t, J = 7.6 Hz, 2 H), 7.43-7.30 (m, 5 H), 5.69 (t, J = 9.0 Hz, 1 H), 4.51 (dd, J = 14.5, 10.1 Hz, 1 H), 4.02 (dd, J = 14.6, 7.8 Hz, 1 H); ^{13}C NMR (150 MHz, $CDCl_3$): δ 163.9, 141.0, 131.3, 128.7, 128.3, 128.2, 128.2, 127.6, 125.6, 80.9, 63.1

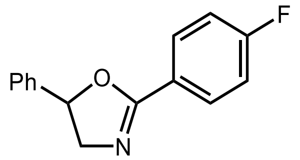

2-(4-Fluorophenyl)-5-phenyl-4,5-dihydrooxazole (4): This compound was prepared according to the General Procedure, using styrene (28 μL, 0.25 mmol) and 4-fluorobenzamide (139 mg, 0.5 mmol). After purification by column chromatography SiO_2 (10% to 15% EtOAc in hexanes), the title compound was isolated as a colorless oil (28 mg, 47% yield). Spectral data matched with previously reported literature.[1]

^{1}H NMR (600 MHz, $CDCl_3$): δ 8.02 (d, *J* = 7.7 Hz, 2 H), 7.57-7.48 (m, 1 H), 7.48-7.41 (m, 2 H), 7.34 (dd, *J* = 8.1,5.5 Hz, 2 H), 7.08 (t, *J* = 8.4 Hz, 2 H), 5.71-5.62 (m, 1 H), 4.49 (dd, *J* = 14.7, 10.3 Hz, 1 H), 3.98 (dd, *J* = 14.9, 7.9 Hz, 1 H); ^{13}C NMR (150 MHz, $CDCl_3$): δ 163.9, 163.4, 161.8, 136.8, 136.8, 131.5, 128.4, 128.2, 127.6, 127.5, 127.4, 115.8, 115.7, 63.1.

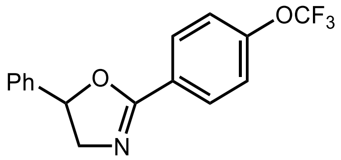

5-Phenyl-2-(4-(trifluoromethoxy)phenyl)-4,5-dihydrooxazole (5): This compound was prepared according to the General Procedure, using styrene (28 μL, 0.25 mmol) and 4-(trifluoromethoxy)benzamide (205 mg, 1.0 mmol). After purification by column chromatography SiO_2 (10% to 15% EtOAc in hexanes), the title compound was isolated as a colorless oil (41 mg, 53% yield).

^{1}H NMR (600 MHz, $CDCl_3$): δ 8.05 (d, *J* = 7.3 Hz, 2 H), 7.55-7.49 (m, 2 H), 7.49-7.41 (m, 3 H), 7.31-7.24 (m, 2 H), 5.63 (dd, *J* = 10.1, 7.9, Hz, 1 H), 4.50 (dd, *J* = 14.9, 10.1 Hz, 1 H), 3.97 (dd,

J = 14.9, 7.9 Hz, 1 H): ^{13}C NMR (150 MHz, $CDCl_3$): 163.9, 143.3, 131.6, 131.3, 130.4, 128.7, 128.5, 128.3, 127.3, 124.2, 122.9, 80.1, 63.1; ^{19}F NMR (375 MHz, $CDCl_3$): δ -63.3 (s, 3 F); IR (neat): 2961, 1741, 1645, 1334, 1198, 1110, 1061, 1022, 898, 670. 544 cm^{-1}; HRMS (ESI) *m/z* calcd for $C_{18}H_{20}NO_2S$ [$(M+H)^+$] 308.0820, found 308.0829.

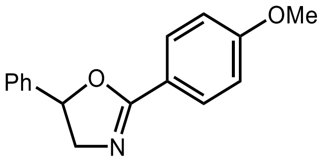

2-(4-Methoxyphenyl)-5-phenyl-4,5-dihydrooxazole (6): This compound was prepared according to the General Procedure, using styrene (28 μL, 0.25 mmol) and 4-methoxybenzamide (151 mg, 1.0 mmol). After purification by column chromatography SiO_2 (10% to 15% EtOAc in hexanes), the title compound was isolated as a colorless oil (34 mg, 54% yield). Spectral data matched with previously reported literature.[1]

^{1}H NMR (600 MHz, $CDCl_3$): δ 8.01 (d, J = 7.3 Hz, 2 H), 7.50 (t, J = 7.3 Hz, 1 H), 7.43 (t, J = 7.5 Hz, 3 H), 7.34-7.27 (m, J = 8.8 Hz, 2 H), 6.95 (d, J = 9.0 Hz, 2 H), 5.63 (t, J = 9.0 Hz, 1 H), 4.45 (dd, J = 14.9, 10.1 Hz, 1 H), 4.00 (dd, J = 15.0, 8.1 Hz, 1 H), 3.82 (s, 3 H); ^{13}C NMR (150 MHz, $CDCl_3$): δ 163.8, 162.2, 141.1, 130.0, 128.8, 128.2, 125.7, 120.0, 113.7, 80.9, 63.0, 55.4.

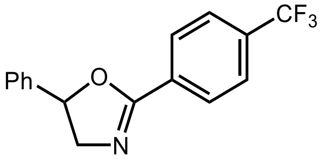

5-Phenyl-2-(4-(trifluoromethyl)phenyl)-4,5-dihydrooxazole (7): This compound was prepared according to the General Procedure, using styrene (28 μL, 0.25 mmol) and 4-(trifluoromethyl)benzamide (189 mg, 1.0 mmol). After purification by column chromatography

SiO_2 (2% EtOAc in hexanes), the title compound was isolated as a colorless oil (40 mg, 55% yield). Spectral data matched with previously reported literature.[1]

^{1}H NMR (600 MHz, $CDCl_3$): δ .71 (d, *J* = 3.3 Hz, 1 H), 7.58-7.44 (m, 1 H), 7.44-7.24 (m, 6 H), 7.12 (dd, *J* = 5.0, 3.9 Hz, 1 H), 5.38 (dd, *J* = 9.5, 8.4 Hz, 1 H), 4.80 (dd, *J* = 9.9, 8.4, Hz, 1 H), 4.28 (t, *J* = 8.3 Hz, 1 H);^{13}C NMR (150 MHz, $CDCl_3$): δ 160.4, 142.0, 130.7, 130.1, 130.0, 128.7, 127.6, 126.7, 75.2, 70.2.

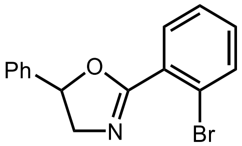

2-(2-Bromophenyl)-5-phenyl-4,5-dihydrooxazole (8): This compound was prepared according to the General Procedure with slight modification, using 4-iodoanisole (12 mg, 0.05 mmol) as a catalyst, styrene (28 μL, 0.25 mmol) and 2-bromobenzamide (200 mg, 1.0 mmol). After purification by column chromatography SiO_2 (10% to 15% EtOAc in hexanes), the title compound was isolated as a colorless oil (35 mg, 47% yield).

^{1}H NMR (600 MHz, $CDCl_3$): δ 7.86-7.77 (m, 1 H), 7.69 (d, *J* = 8.1 Hz, 1 H), 7.44-7.39 (m, 4 H), 7.39-7.34 (m, 2 H), 7.34-7.29 (m, 1 H), 5.69 (dd, *J* = 10.1, 8.6 Hz, 1 H), 4.56 (dd, *J* = 14.9, 10.5 Hz, 1 H), 4.07 (dd, *J* = 14.7, 8.4 Hz, 1 H); ^{13}C NMR (150 MHz, $CDCl_3$): δ 163.1, 140.6, 134.0, 131.7, 131.4, 129.3, 128.8, 128.4, 127.1, 125.9, 121.9, 81.3, 63.4; IR (neat): 2965, 1731, 1640, 1339, 1188, 1112, 1059, 1020, 899, 671.540 cm^{-1}; HRMS (ESI) *m/z* calcd for $C_{18}H_{20}NO_2S$ $[(M+H)^+]$ 302.0102, found 302.0112.

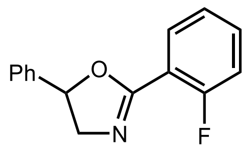

2-(2-Fluorophenyl)-5-phenyl-4,5-dihydrooxazole (9): This compound was prepared according to the General Procedure, using styrene (28 μL, 0.25 mmol) and 2-fluorobenzamide (139 mg, 1.0 mmol). After purification by column chromatography SiO_2 (10% to 15% EtOAc in hexanes), the title compound was isolated as a colorless oil (38 mg, 63% yield).

^1H NMR (600 MHz, $CDCl_3$): δ 7.95 (dt, *J* = 7.5, 1.7 Hz, 1 H), 7.52-7.43 (m, 1 H), 7.43-7.32 (m, 5 H), 7.24-7.17 (m, 2 H), 5.66 (dd, *J* = 9.9, 8.1 Hz, 1 H), 4.55 (dd, *J* = 15.0, 10.3 Hz, 1 H), 4.06 (dd, *J* = 15.0, 8.1 Hz, 1 H); ^{13}C NMR (150 MHz, $CDCl_3$): δ 162.2, 160.6, 160.5, 160.5, 140.8, 133.0, 132.9, 131.1, 128.8, 128.3, 125.7, 124.0, 124.0, 116.8, 116.6, 115.9, 115.9, 80.4, 63.5; ^{19}F NMR (375 MHz, $CDCl_3$): δ -109.6 (m, 1 F); IR (neat): 2932, 1649, 1490, 1337, 1059, 1024, 945, 850, 688. 542 cm^{-1}; HRMS (ESI) *m/z* calcd for $C_{18}H_{20}NO_2S$ $[(M+H)^+]$ 242.0903, found 242.0902.

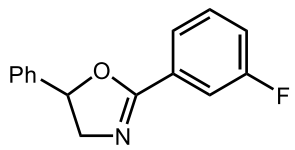

2-(3-Fluorophenyl)-5-phenyl-4,5-dihydrooxazole (10): This compound was prepared according to the General Procedure with slight modification, using 4-iodoanisole (12 mg, 0.05 mmol) as a catalyst, styrene (28 μL, 0.25 mmol) and 3-fluorobenzamide (139 mg, 1.0 mmol). After purification by column chromatography SiO_2 (10% to 15% EtOAc in hexanes), the title compound was isolated as a colorless oil (31.5 mg, 52% yield).

^1H NMR (600 MHz, $CDCl_3$): δ 7.82 (d, *J* = 7.3 Hz, 1 H), 7.72 (d, *J* = 9.2 Hz, 1 H), 7.47-7.30 (m, 6 H), 7.24-7.16 (m, 1 H), 5.74-5.63 (m, 1 H), 4.50 (dd, *J* = 15.0, 10.3 Hz, 1 H), 4.02 (dd, *J* = 14.7,

8.1 Hz, 1 H); ^{13}C NMR (150 MHz, $CDCl_3$): δ 163.3, 163.0, 161.7, 140.7, 130.1, 130.0, 129.7, 129.6, 128.9, 128.4, 125.7, 124.0, 124.0, 118.5, 118.4, 115.4, 115.2, 81.3, 63.1; ^{19}F NMR (375 MHz, $CDCl_3$): δ -112.9 (m, 1 F); IR (neat): 2938, 1642, 1493, 1327, 1055, 1027, 948, 854, 689. 544 cm^{-1}; HRMS (ESI) *m/z* calcd for $C_{15}H_{13}FNO$ $[(M+H)]^+$ 242.0903, found 242.0905.

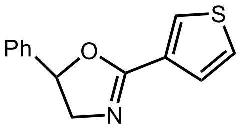

5-Phenyl-2-(thiophen-3-yl)-4,5-dihydrooxazole (11): This compound was prepared according to the General Procedure with slight modification, using 4-iodoanisole (12 mg, 0.05 mmol) as a catalyst, styrene (28 μL, 0.25 mmol) and thiophene-3-carboxamide (127 mg, 1.0 mmol). After purification by column chromatography SiO_2 (10% to 15% EtOAc in hexanes), the title compound was isolated as a colorless oil (31 mg, 54% yield). Spectral data matched with previously reported literature.[1]

^1H NMR (600 MHz, $CDCl_3$): δ 7.67 (d, J = 2.9 Hz, 1 H), 7.51-7.48 (m, 1 H), 7.43-7.38 (m, 3 H), 7.38-7.31 (m, 4 H), 7.11 (dd, J = 4.8, 3.7 Hz, 1 H), 5.67 (dd, J = 9.9, 8.1 Hz, 1 H), 4.47 (dd, J = 14.7, 10.3 Hz, 1 H), 3.99 (dd, J = 14.7, 8.1 Hz, 1 H); ^{13}C NMR (150 MHz, $CDCl_3$): 159.8, 140.6, 130.4, 130.2, 130.0, 128.8, 128.4, 127.6, 125.8, 81.6, 63.1.

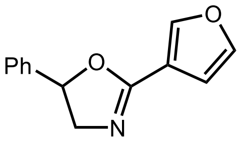

2-(Furan-3-yl)-5-phenyl-4,5-dihydrooxazole (12): This compound was prepared according to the General Procedure, using styrene (28 μL, 0.25 mmol) and furan-3-carboxamide (111 mg,

1.0 mmol). After purification by column chromatography SiO_2 (10% to 15% EtOAc in hexanes), the title compound was isolated as a colorless oil (29 mg, 55% yield). Spectral data matched with previously reported literature.[2]

^{1}H NMR (600 MHz, $CDCl_3$): δ 7.58 (s, 1 H), 7.60 (s, 1 H), 7.41-7.34 (m, 2 H), 7.34-7.28 (m, 3 H), 7.15 (br. s., 1 H), 6.63-6.46 (m, 1 H), 5.42 (t, *J* = 9.0 Hz, 1 H), 4.82 (t, *J* = 9.4 Hz, 1 H), 4.31 (t, *J* = 8.3 Hz, 1 H); ^{13}C NMR (150 MHz, $CDCl_3$): 163.4, 144.9, 131.6, 128.4, 128.3, 127.8, 127.2, 122.9, 109.7, 76.2, 62.8.

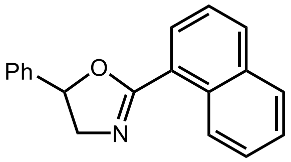

2-(Naphthalen-1-yl)-5-phenyl-4,5-dihydrooxazole (13): This compound was prepared according to the General Procedure with slight modification, using 4-iodoanisole (12 mg, 0.05 mmol) as a catalyst, styrene (28 μL, 0.25 mmol) and 1-naphthamide (171 mg, 1.0 mmol). After purification by column chromatography SiO_2 (10% to 15% EtOAc in hexanes), the title compound was isolated as a colorless oil (32 mg, 47% yield). Spectral data matched with previously reported literature.[3]

^{1}H NMR (600 MHz, $CDCl_3$): δ 9.09-8.99 (m, 1 H), 8.10-8.06 (m, 1 H), 7.84-7.76 (m, 1 H), 7.75-7.60 (m, 1 H), 7.53-7.44 (m, 1 H), 7.37 (d, *J* = 5.9 Hz, 1 H), 7.40 (d, *J* = 7.0 Hz, 1 H), 7.21-7.16 (m, 1 H), 7.15-7.11 (m, 1 H), 5.62-5.48 (m, 1 H), 4.55-4.48 (m, 1 H), 4.10-3.97 (m, 1 H); ^{13}C NMR (150 MHz, $CDCl_3$): δ 163.8, 141.2, 133.7, 132.1, 131.2, 129.2, 128.8, 128.5, 128.3, 127.4, 126.4, 126.1, 125.8, 124.7, 124.2, 79.9, 63.9.

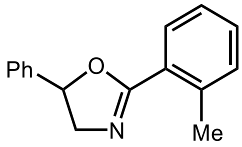

5-Phenyl-2-(*o*-tolyl)-4,5-dihydrooxazole (14): This compound was prepared according to the General Procedure, using styrene (28 μL, 0.25 mmol) and *o*-toluamide (135 mg, 1.0 mmol). After purification by column chromatography SiO_2 (10% to 15% EtOAc in hexanes), the title compound was isolated as a colorless oil (40 mg, 68% yield). Spectral data matched with previously reported literature.[3]

^{1}H NMR (600 MHz, $CDCl_3$): δ 7.94 (d, *J* = 7.6 Hz, 1 H), 7.52-7.34 (m, 7 H), 7.34-7.16 (m, 3 H), 5.65 (dd, *J* = 9.9, 8.2 Hz, 1 H), 4.56 (dd, *J* = 14.9, 10.3 Hz, 1 H), 4.07 (dd, *J* = 14.8, 7.9 Hz, 1 H), 2.68 (s, 3 H);^{13}C NMR (150 MHz, $CDCl_3$): δ 164.4, 141.2, 139.0, 131.3, 130.6, 130.0, 128.8, 128.2, 127.0, 125.7, 125.6, 80.2, 63.6, 22.0.

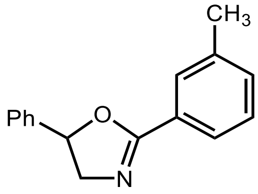

5-Phenyl-2-(*m*-tolyl)-4,5-dihydrooxazole (15): This compound was prepared according to the General Procedure, using styrene (28 μL, 0.25 mmol) and *m*-toluamide (135 mg, 1.0 mmol). After purification by column chromatography SiO_2 (10% to 15% EtOAc in hexanes), the title compound was isolated as a yellow oil (42 mg, 71% yield). Spectral data matched with previously reported literature.[3]

^{1}H NMR (600 MHz, $CDCl_3$): δ 8.01-7.81 (m, 2 H), 7.51-7.32 (m, 7 H), 5.82-5.62 (m, 1 H), 4.49 (dd, *J* = 14.8, 10.1 Hz, 1 H), 4.00 (dd, *J* = 14.8, 7.9, Hz, 1 H), 2.59-2.39 (m, 3 H);^{13}C NMR (150 MHz, $CDCl_3$): 164.4, 141.2, 139.0, 131.3, 130.6, 130.0, 128.8, 128.2, 126.9, 125.7, 125.6, 80.2, 63.6, 22.0.

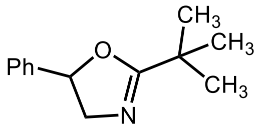

2-(*tert*-Butyl)-5-phenyl-4,5-dihydrooxazole (16): This compound was prepared according to the General Procedure, using styrene (28 μL, 0.25 mmol) and pivalamide (101 mg, 1.0 mmol). After purification by column chromatography SiO_2 (10% to 15% EtOAc in hexanes), the title compound was isolated as a colorless oil (24 mg, 47% yield). Spectral data matched with previously reported literature.[1]

^{1}H NMR (600 MHz, $CDCl_3$): 7.38 (t, *J* = 7.5 Hz, 2 H), 7.32 (t, *J* = 7.2 Hz, 1 H), 7.28 (s, 2 H), 5.56-5.37 (m, 1 H), 4.27 (dd, *J* = 13.9, 10.3, Hz, 1 H), 3.76 (dd, *J* = 13.9, 7.7, Hz, 1 H), 1.34-1.29 (m, 9 H);^{13}C NMR (150 MHz, $CDCl_3$): 174.1, 141.6, 128.7, 128.0, 125.5, 62.9, 33.3, 27.7.

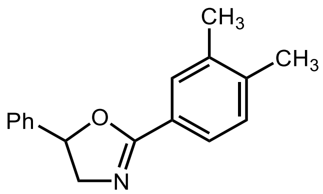

2-(3,4-Dimethylphenyl)-5-phenyl-4,5-dihydrooxazole (17): This compound was prepared according to the General Procedure, using styrene (28 μL, 0.25 mmol) and 3,4-dimethylbenzamide (149 mg, 1.0 mmol). After purification by column chromatography SiO_2 (10% to 15% EtOAc in hexanes), the title compound was isolated as a white solid (44 mg, 70% yield).

^{1}H NMR (600 MHz, $CDCl_3$): 7.82 (br. s., 1 H), 7.75 (d, *J* = 7.6 Hz, 1 H), 7.43-7.32 (m, 5 H), 7.21 (d, *J* = 7.6 Hz, 1 H), 5.65 (t, *J* = 8.8 Hz, 1 H), 4.48 (dd, *J* = 14.2, 10.5 Hz, 1 H), 3.99 (dd, *J* = 14.5, 7.9 Hz, 1 H), 2.32 (d, *J* = 7.3 Hz, 7 H); ^{13}C NMR (150 MHz, $CDCl_3$): 164.3, 141.3, 140.6, 136.8, 129.7, 129.4, 128.8, 128.3, 125.8, 125.8, 125.1, 80.9, 63.2, 19.9, 19.7 IR (neat): 2932, 1645, 1491, 1320, 1059, 1022, 948, 857, 690. 554 cm^{-1}; HRMS (ESI) *m/z* calcd for $C_{18}H_{20}NO_2S$ [$(M+H)^+$] 252.1310, found 252.1311.

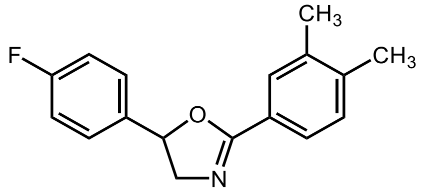

2-(3,4-Dimethylphenyl)-5-(4-fluorophenyl)-4,5-dihydrooxazole (18): This compound was prepared according to the General Procedure, using 1-fluoro-4-vinylbenzene (30 μL, 0.25 mmol) and 3,4-dimethylbenzamide (149 mg, 1.0 mmol). After purification by column chromatography SiO_2 (10% to 15% EtOAc in hexanes), the title compound was isolated as a colorless oil (51 mg, 76% yield).

^{1}H NMR (600 MHz, $CDCl_3$): δ 7.80 (s, 1 H), 7.73 (d, *J* = 7.7 Hz, 1 H), 7.33 (dd, *J* = 8.6, 5.3, Hz, 2 H), 7.21 (d, *J* = 7.7 Hz, 1 H), 7.07 (t, *J* = 8.8 Hz, 2 H), 5.63 (dd, *J* = 9.9, 7.7, Hz, 1 H), 4.46 (dd, *J* = 14.7, 9.9 Hz, 1 H), 3.95 (dd, *J* = 14.7 , 7.7 Hz, 1 H), 2.32 (d, *J* = 7.7 Hz, 7 H);^{13}C NMR (150 MHz, $CDCl_3$):δ 164.2, 141.2, 140.5, 136.7, 129.7, 129.3, 128.8, 128.2, 125.7, 125.1, 80.9, , 63.2, 19.9, 19.6; ^{19}F NMR (375 MHz, $CDCl_3$): δ -108.4 (m, 1 F); IR (neat): 2938, 1642, 1493, 1327, 1055, 1027, 948, 854, 689. 544 cm^{-1}; HRMS (ESI) *m/z* calcd for $C_{18}H_{20}NO_2S$ [$(M+H)^+$] 270.1216, found 270.1215.

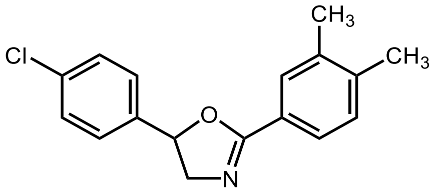

5-(4-Chlorophenyl)-2-(3,4-dimethylphenyl)-4,5-dihydrooxazole (19): This compound was prepared according to the General Procedure, using 1-chloro-4-vinylbenzene (32 μL, 0.25 mmol) and 3,4-dimethylbenzamide (149 mg, 1.0 mmol). After purification by column chromatography SiO_2 (10% to 15% EtOAc in hexanes), the title compound was isolated as a colorless oil (48 mg, 67% yield).

^{1}H NMR (600 MHz, $CDCl_3$): δ 7.80 (s, 1 H), 7.73 (d, *J* = 7.8 Hz, 1 H), 7.36 (d, *J* = 8.3 Hz, 3 H), 7.29 (br. s., 2 H), 7.21 (d, *J* = 7.8 Hz, 1 H), 5.67-5.55 (m, 1 H), 4.47 (dd, *J* = 14.6, 10.3 Hz, 1 H), 3.93 (dd, *J* = 14.6, 7.6 Hz, 1 H), 2.32 (m, 6 H); ^{13}C NMR (150 MHz, $CDCl_3$): δ 164.2, 141.2, 140.5, 136.7, 129.7, 129.3, 128.8, 128.2, 125.8, 125.7, 125.1, 80.9, 63.2, 19.9, 19.6. IR (neat): 2935, 1652, 1483, 1327, 1056, 1029, 944, 857, 681. 549 cm^{-1}; HRMS (ESI) *m/z* calcd for $C_{18}H_{20}NO_2S$ [$(M+H)^+$] 286.0920, found 286.0921.

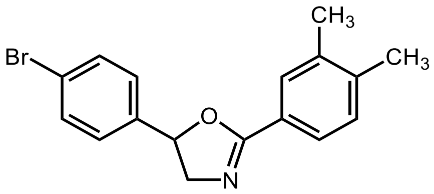

5-(4-Bromophenyl)-2-(3,4-dimethylphenyl)-4,5-dihydrooxazole (20): This compound was prepared according to the General Procedure, using 1-bromo-2-vinylbenzene (32 μL, 0.25 mmol) and 3,4-dimethylbenzamide (149 mg, 1.0 mmol). After purification by column chromatography SiO_2 (10% to 15% EtOAc in hexanes), the title compound was isolated as a colorless oil (41 mg, 50% yield).

^{1}H NMR (600 MHz, $CDCl_3$): δ 7.81 (s, 1 H), 7.75 (d, *J* = 7.7 Hz, 1 H), 7.37 (d, *J* = 8.4 Hz, 3 H), 7.30 (d, *J* = 8.4 Hz, 2 H), 7.22 (d, *J* = 8.1 Hz, 1 H), 5.68 - 5.55 (m, 1 H), 4.48 (dd, *J* = 10.3, 14.7 Hz, 1 H), 3.95 (dd, *J* = 7.7, 14.7 Hz, 1 H); ^{13}C NMR (150 MHz, $CDCl_3$): δ 164.2, 140.7, 139.8, 136.8, 134.1, 129.8, 129.4, 129.0, 128.7, 127.1, 125.8, 124.9, 80.1, 63.2, 29.7, 19.9, 19.7 IR (neat): 2938, 1642, 1493, 1327, 1055, 1027, 948, 854, 689, 544 cm^{-1}; HRMS (ESI) *m/z* calcd for $C_{18}H_{20}NO_2S$ [$(M+H)^+$] 330.0415, found 330.0411.

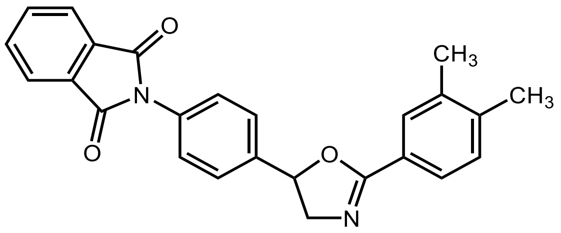

2-(2-(3,4-Dimethylphenyl)-4,5-dihydrooxazol-5-yl)-5-phenylisoindoline-1,3-dione (21): This compound was prepared according to the General Procedure, using 2-(4-vinylphenyl)isoindoline-1,3-dione (62 mg, 0.25 mmol) and 3,4-dimethylbenzamide (149 mg, 1.0 mmol). After purification by column chromatography SiO_2 (10% to 15% EtOAc in hexanes), the title compound was isolated as a colorless oil (74 mg, 75% yield).

^{1}H NMR (600 MHz, $CDCl_3$): δ 7.96 (dd, *J* = 5.5,2.9 Hz, 2 H), 7.85-7.77 (m, 3 H), 7.75 (d, *J* = 7.7 Hz, 1 H), 7.53-7.45 (m, 4 H), 7.21 (d, *J* = 7.7 Hz, 1 H), 5.70 (dd, *J* = 7.9, 9.7 Hz, 1 H), 4.50 (dd, *J* = 10.3, 14.7 Hz, 1 H), 4.02 (dd, *J* = 7.7, 14.7 Hz, 1 H), 2.32 (d, *J* = 4.8 Hz, 6 H);^{13}C NMR (150 MHz, $CDCl_3$): δ 167.1, 164.2, 141.1, 140.6, 136.8, 134.4, 131.6, 131.5, 129.7, 129.3, 126.8, 126.5, 125.8, 124.9, 123.8, 80.3, 63.0, 19.9, 19.6; IR (neat): 2922, 1711, 1651 1609, 1516, 1379,1329, 1215, 1082, 884, 715, 529 cm^{-1}; HRMS (ESI) *m/z* calcd for $C_{18}H_{20}NO_2S$ [$(M+H)^+$] 397.1474, found 397.1552.

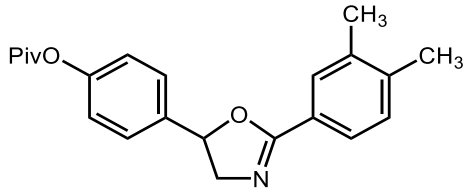

4-(2-(3,4-Dimethylphenyl)-4,5-dihydrooxazol-5-yl)phenyl pivalate (22): This compound was prepared according to the General Procedure, using 4-vinylphenyl pivalate (52 mg, 0.25 mmol) and 3,4-dimethylbenzamide (149 mg, 1.0 mmol). After purification by column chromatography SiO_2 (10% to 15% EtOAc in hexanes), the title compound was isolated as a colorless oil (56 mg, 64% yield).

^{1}H NMR (600 MHz, $CDCl_3$): δ 7.80 (s, 1 H), 7.74 (d, *J* = 7.7 Hz, 1 H), 7.43-7.33 (m, 2 H), 7.21 (d, *J* = 7.7 Hz, 1 H), 7.11-7.02 (m, 2 H), 5.64 (dd, *J* = 9.9, 7.7 Hz, 1 H), 4.46 (dd, *J* = 14.7, 10.3 Hz, 1 H), 3.97 (dd, *J* = 14.7, 7.7 Hz, 1 H), 2.32 (d, *J* = 7.0 Hz, 6 H), 1.36 (s, 9 H);^{13}C NMR (150 MHz, $CDCl_3$): δ 177.0, 164.2, 151.0, 140.6, 138.5, 136.8, 129.7, 129.4, 126.9, 125.8, 125.0, 121.9, 80.4, 63.1, 39.1, 27.1, 19.9, 19.7; IR (neat): 3038, 2927, 1651, 1609, 1513, 1255, 837, 763, 700 cm^{-1}; HRMS (ESI) *m/z* calcd for $C_{18}H_{20}NO_2S$ [$(M+H)^+$] 352.1834, found 352.1833.

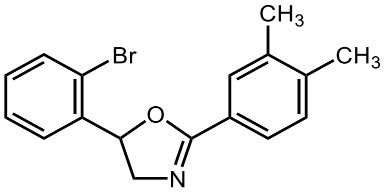

5-(2-Bromophenyl)-2-(3,4-dimethylphenyl)-4,5-dihydrooxazole (23): This compound was prepared according to the General Procedure, using 1-bromo-3-vinylbenzene (32 μL, 0.25 mmol) and 3,4-dimethylbenzamide (149 mg, 1.0 mmol). After purification by column chromatography SiO_2 (10% to 15% EtOAc in hexanes), the title compound was isolated as a colorless oil (42 mg, 51% yield).

^{1}H NMR (600 MHz, $CDCl_3$): δ 7.82 (s, 1 H), 7.76 (d, *J* = 7.7 Hz, 1 H), 7.51 (s, 1 H), 7.48 (d, *J* = 7.3 Hz, 1 H), 7.30-7.27 (m, 3 H), 7.27-7.25 (m, 1 H), 7.23 (d, *J* = 8.1 Hz, 1 H), 5.62 (dd, *J* = 9.9, 8.1 Hz, 1 H), 4.49 (dd, *J* = 14.7, 10.3 Hz, 1 H), 3.96 (dd, *J* = 14.7, 7.7 Hz, 1 H), 2.34 (d, *J* = 6.2 Hz, 6 H);^{13}C NMR (150 MHz, $CDCl_3$): δ 164.2, 143.6, 140.8, 136.9, 131.3, 130.4, 129.8, 129.4, 128.7, 125.8, 124.8, 124.3, 122.9, 79.9, 63.2, 19.9, 19.7. IR (neat): 2937, 1644, 1492, 1327, 1055, 1026, 944, 851, 689, 542 cm^{-1}; HRMS (ESI) *m/z* calcd for $C_{18}H_{20}NO_2S$ [$(M+H)^+$] 330.0415, found 330.0416.

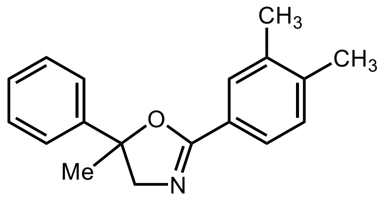

2-(3,4-Dimethylphenyl)-5-methyl-5-phenyl-4,5-dihydrooxazole (24): This compound was prepared according to the General Procedure with slight modification, using 4-iodoanisole (12 mg, 0.05 mmol), prop-1-en-2-ylbenzene ((32.5 μL, 0.25 mmol) and 3,4-dimethylbenzamide (149 mg, 1.0 mmol) in acetonitrile (2ml). After purification by column chromatography SiO_2 (10% to 20% EtOAc in hexanes), the title compound was isolated as a brown oil (37 mg, 56% yield).

^{1}H NMR (600 MHz, $CDCl_3$): 7.83 (s, 1 H), 7.78 (d, *J* = 7.7 Hz, 1 H), 7.46 - 7.40 (m, 2 H), 7.37 (t, *J* = 7.7 Hz, 2 H), 7.33 - 7.28 (m, 1 H), 7.22 (d, *J* = 7.7 Hz, 1 H), 4.13 (s, 2 H), 2.33 (s, 6 H), 1.80 (s,3H);^{13}CNMR(150MHz,$CDCl_3$):163.3, 145.6, 140.4, 136.7, 129.7, 129.2, 128.5, 127.3, 125.7, 125.5, 124.3, 86.5, 69.0, 28.1, 19.9,19.7 IR (neat): 2923, 1647, 1497, 1446, 1349, 1265, 1120, 1079, 1063, 1012, 917, 869, 761, 699, 563 cm^{-1}; HRMS (ESI) *m/z* calcd for $C_{18}H_{20}NO_2S$ [$(M+H)^+$] 266.1467, found 266.1545.

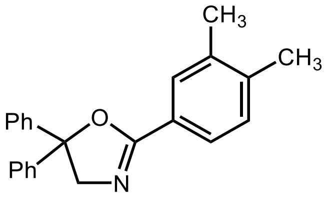

2-(3,4-Dimethylphenyl)-5,5-diphenyl-4,5-dihydrooxazole (25): This compound was prepared according to the General Procedure with slight modification, using 4-iodoanisole (12 mg, 0.05 mmol), ethene-1,1-diyldibenzene (45 mg, 0.25 mmol) and 3,4-dimethylbenzamide (149 mg, 1.0 mmol) in acetonitrile (2 mL). After purification by column chromatography SiO_2 (10% to 20% EtOAc in hexanes), the title compound was isolated as a colorless oil (53 mg, 65% yield).

^{1}H NMR (600 MHz, $CDCl_3$): 7.88 (s, 1 H), 7.83 (d, *J* = 7.7 Hz, 1 H), 7.43 (d, *J* = 7.7 Hz, 4 H), 7.36 (t, *J* = 7.5 Hz, 4 H), 7.32-7.28 (m, 2 H), 7.22 (d, *J* = 8.1 Hz, 1 H), 4.66 (s, 2 H), 2.33 (s, 6 H); ^{13}C NMR (150 MHz, $CDCl_3$): δ 163.2, 144.3, 140.6, 136.8, 129.7, 129.3, 128.4, 127.7, 125.9, 125.8, 90.0, 69.1, 19.9, 19.7 IR (neat): 2926, 1651, 1492, 1450. 1079, 752, 696, 537 cm^{-1}; HRMS (ESI) *m/z* calcd for $C_{18}H_{20}NO_2S$ [$(M+H)^+$] 328.1623, found 328.1701.

4. Kinetic studies

4.1 Procedure for gas chromatography time courses: To an 8 mL vial equipped with a stir bar was added catalyst (0.10 mmol), benzamide (242 mg, 2.0 mmol), $LiBF_4$ (94mg, 1.0 mmol) and F-TEDA-BF_4 (264 mg, 0.75 mmol). Then, $MeNO_2$ (4.0 mL) was added via syringe, followed by styrene (57.2 µl, 0.5 mmol) and 1,3-dibromobenzene (60 µl, 0.5 mmol, internal standard). Time courses were monitored using GC by taking aliquots. The aliquots were quenched by filtering through a plug of sodium thiosulfate. The initial rates of the reactions were obtained from the reaction time courses.

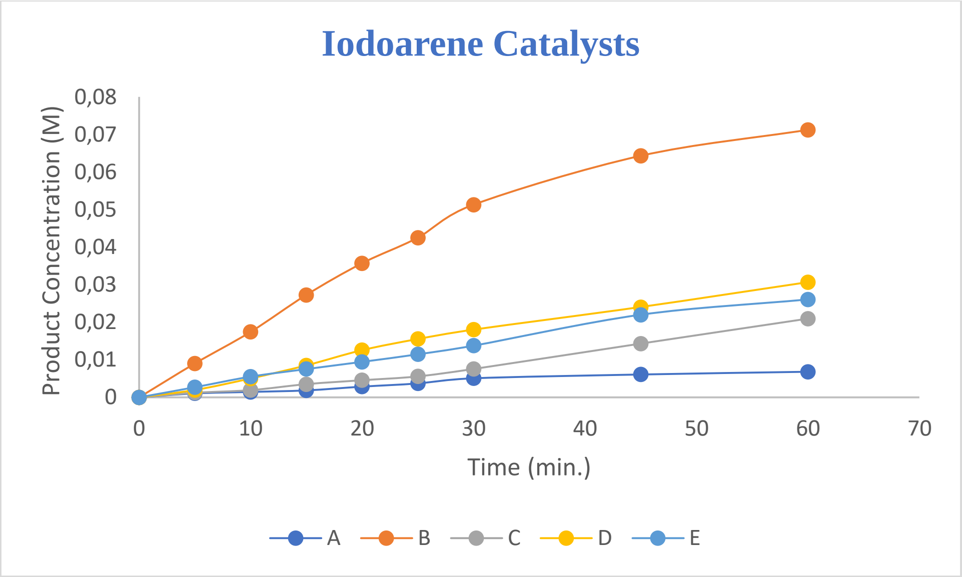

Figure S1: Initial rate study of standard reaction using different iodoarene catalysts where A = *p*-trifluoromethyl-iodobenzene, B = *p*-iodoanisole, C = *p*-bromoiodobenzene, D = *p*-iodotoluene, E = iodobenzene.

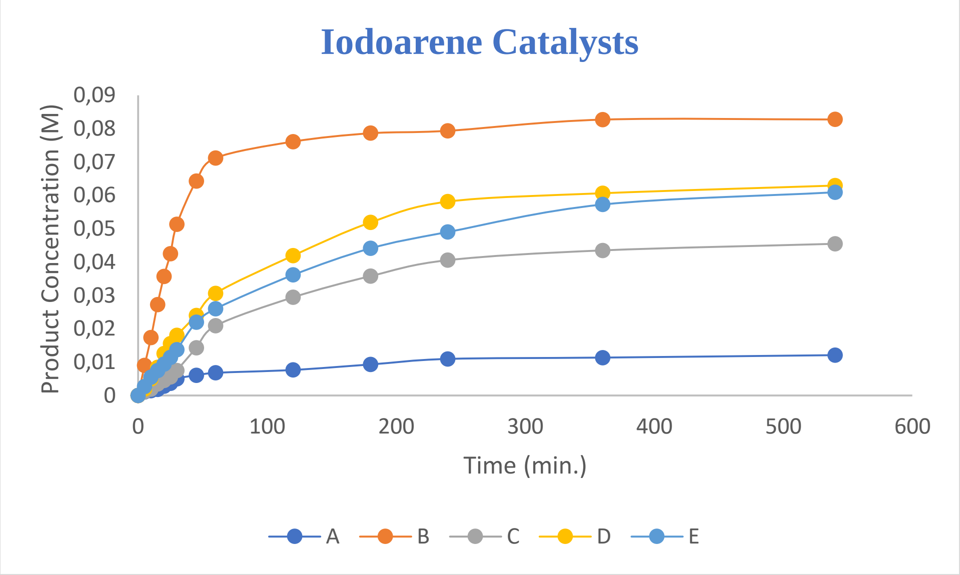

Figure S2: Overall rate study of standard reaction using different iodoarene catalysts where A = *p*-(trifluoromethyl)iodobenzene, B = *p*-iodoanisole, C = *p*-bromoiodobenzene, D = *p*-iodotoluene, E = iodobenzene

		Iodoarene Catalysts				
S.No	Time	A	B	C	D	E
	min	M	M	M	M	M
1	0	0	0	0	0	0
2	5	0.001	0.009	0.0012	0.002	0.0027
3	10	0.0014	0.0174	0.0019	0.005	0.0055
4	15	0.0018	0.0273	0.0035	0.0085	0.0075
5	20	0.0028	0.0357	0.0045	0.0126	0.0094
6	25	0.0037	0.0425	0.0056	0.0155	0.0114
7	30	0.0051	0.0513	0.0075	0.018	0.0137
8	45	0.0061	0.0643	0.0143	0.0241	0.022
9	60	0.0068	0.0712	0.0209	0.0307	0.026
10	120	0.0077	0.0761	0.0294	0.0419	0.0361
11	180	0.0093	0.0786	0.0358	0.0519	0.0441
12	240	0.011	0.0794	0.0406	0.0581	0.0491
13	360	0.0114	0.0827	0.0435	0.0606	0.0573
14	540	0.0121	0.0828	0.0455	0.0629	0.061

Table S1. Raw data for gas chromatography time courses. A = *p*-(trifluoromethyl)iodobenzene, B = *p*-iodoanisole, C = *p*-bromoiodobenzene, D = *p*-iodotoluene, E = iodobenzene.

4.2 Procedure for gas chromatography time courses: To an 8 mL vial equipped with a stir bar was added iodoanisole (23.4 mg, 0.10 mmol), benzamide (242 mg, 2.0 mmol), Li salt (1.0 mmol) and F-TEDA-BF_4 (264 mg, 0.75 mmol). Then, $MeNO_2$ (4.0 mL) was added via syringe, followed by styrene (57.2 µL, 0.5 mmol) and 1,3-dibromobenzene (60 µL, 0.5 mmol, internal standard). Time courses were monitored using GC by taking aliquots. The aliquots were quenched by filtering through a plug of sodium thiosulfate. The initial rates of the reactions were obtained from the reaction time courses.

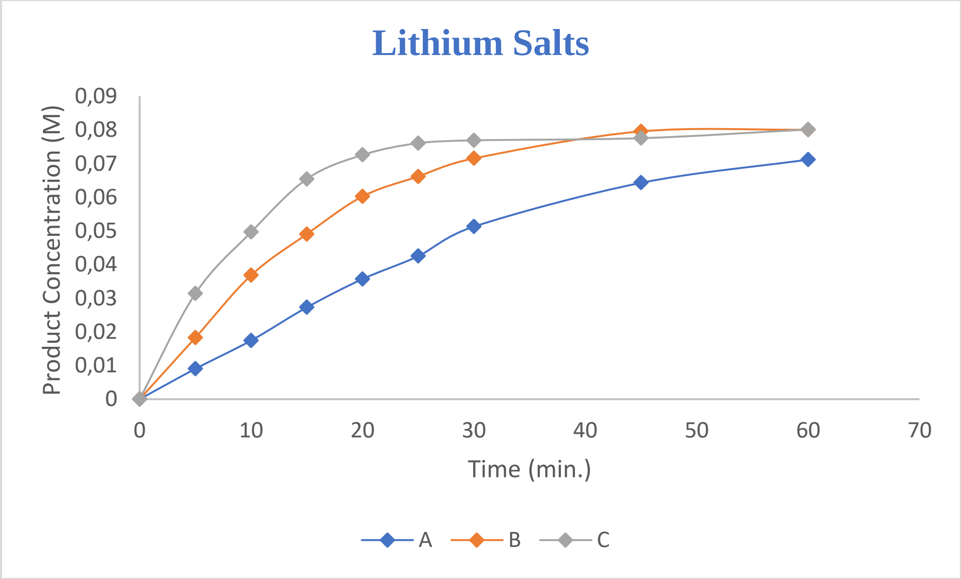

Figure S3: Initial rate study of standard reaction using different lithium salts where A = lithium tetrafluoroborate, B = lithium hexafluorophosphate, C = lithium hexafluoroarsenate.

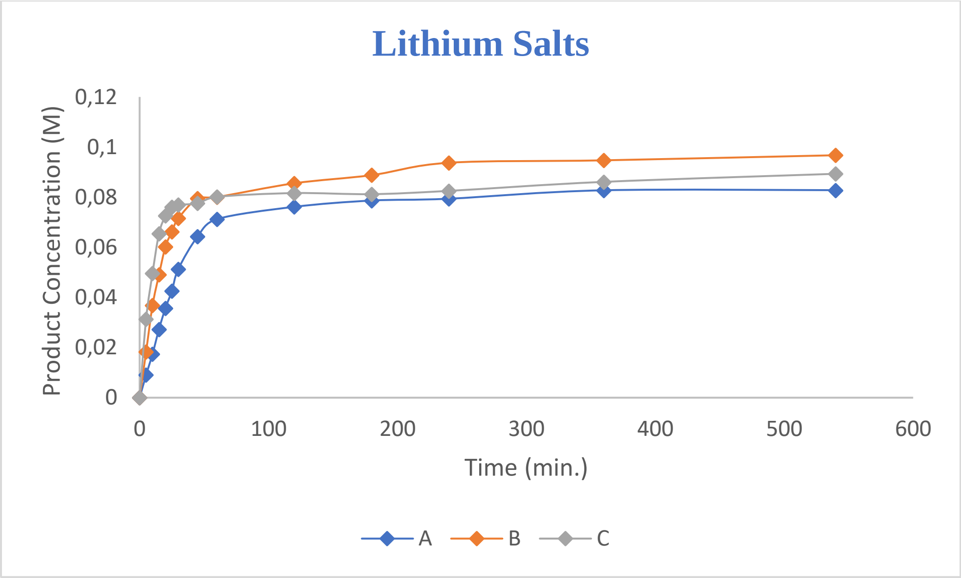

Figure S4: Overall rate study of standard reaction using different lithium salts where A = lithium tetrafluoroborate, B = lithium hexafluorophosphate, C = lithium hexafluoroarsenate.

	Lithium Salts			
S.No	Time	A	B	C
	min	M	M	M
1	0	0	0	0
2	5	0.009	0.0183	0.0314
3	10	0.0174	0.0368	0.0496
4	15	0.0273	0.049	0.0654
5	20	0.0357	0.0602	0.0726
6	25	0.0425	0.0662	0.0761
7	30	0.0513	0.0715	0.0769
8	45	0.0643	0.0796	0.0776
9	60	0.0712	0.0801	0.0801
10	120	0.0761	0.0856	0.0816
11	180	0.0786	0.0888	0.0812
12	240	0.0794	0.0937	0.0825
13	360	0.0827	0.0947	0.0861
14	540	0.0828	0.0967	0.0893

Table S2. Raw data for gas chromatography time courses. A = lithium tetrafluoroborate, B = lithium hexafluorophosphate, C = lithium hexafluoroarsenate.

4.3 Procedure for gas chromatography time courses: To an 8 mL vial equipped with a stir bar was added iodotoluene (22 mg, 0.10 mmol), benzamide (242 mg, 2.0 mmol), $LiBF_4$ (94 mg, 1.0 mmol) and F-TEDA-BF_4 (264 mg, 0.75 mmol). Then, $MeNO_2$ (4.0 mL) was added via syringe, followed by alkene (0.5 mmol) and 1,3-dibromobenzene (60 µL, 0.5 mmol, internal standard). Time courses were monitored using GC by taking aliquots. The aliquots were quenched by filtering through a plug of sodium thiosulfate. The initial rates of the reactions were obtained from the reaction time courses.

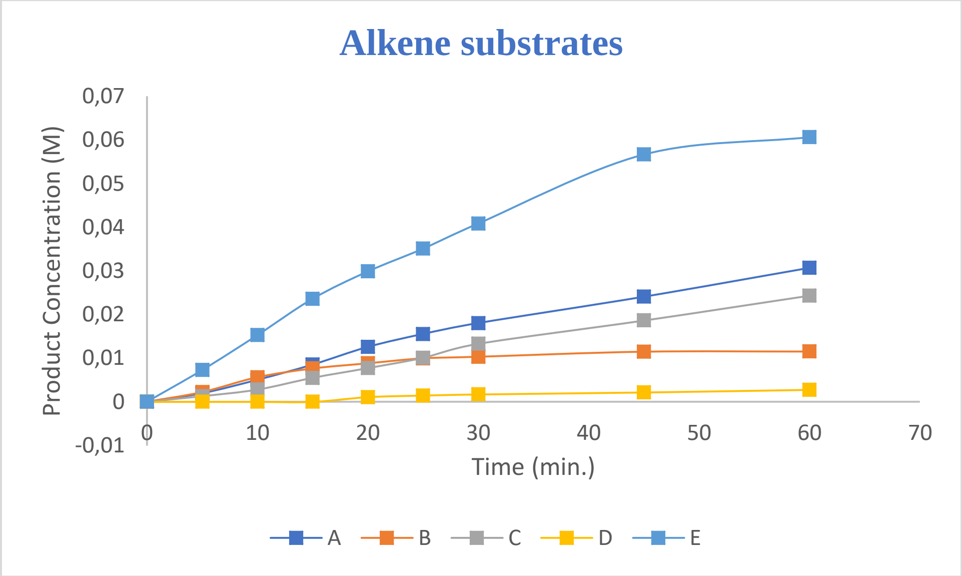

Figure S5: Initial rate study of standard reaction using different alkene substrates where A = styrene, B = *p*-methoxystyrene, C = *p*-bromostyrene, D = *p*-(trifluoromethyl)styrene, E = 4-vinylphenyl pivalate.

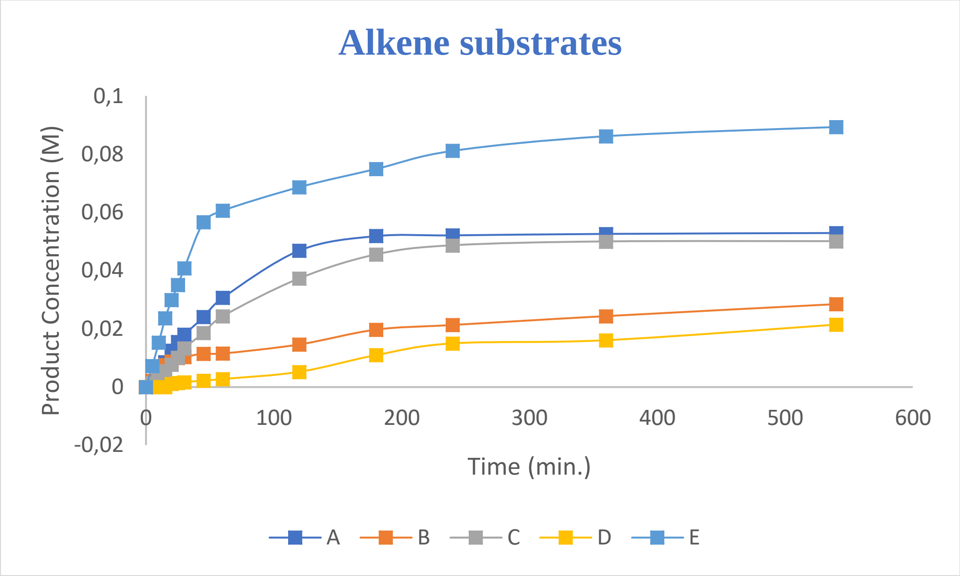

Figure S6: Overall rate study of standard reaction using different alkene substrates where A = styrene, B = *p*-methoxystyrene, C = *p*-bromostyrene, D = *p*-(trifluoromethyl)styrene, E = 4-vinylphenyl pivalate.

		Alkene substrates				
S.No.	Time	A	B	C	D	E
	min	M	M	M	M	M
1	0	0	0	0	0	0
2	5	0.0019	0.0022	0.0013	0	0.0073
3	10	0.005	0.0056	0.0028	0	0.0153
4	15	0.0085	0.0076	0.0055	0	0.0236
5	20	0.0125	0.0088	0.0077	0.0011	0.0299
6	25	0.0155	0.0099	0.0101	0.0014	0.0351
7	30	0.018	0.0103	0.0133	0.0017	0.0408
8	45	0.024	0.0114	0.0186	0.0021	0.0567
9	60	0.0306	0.0115	0.0243	0.0027	0.0606
10	120	0.0469	0.0146	0.0373	0.0052	0.0687
11	180	0.0518	0.0196	0.0455	0.0109	0.0749
12	240	0.0521	0.0212	0.0487	0.015	0.0812
13	360	0.0526	0.0243	0.05	0.0161	0.0862
14	540	0.0529	0.0284	0.0501	0.0214	0.0894

Table S3. Raw data for gas chromatography time courses. A = styrene, B = *p*-methoxystyrene, C = *p*-bromostyrene, D = *p*-(trifluoromethyl)styrene, E = 4-vinylphenyl pivalate.

4.4 Procedure for gas chromatography time courses: To an 8 mL vial equipped with a stir bar was added iodotoluene (22 mg, 0.10 mmol), benzamide (2.0 mmol), $LiBF_4$ (94 mg, 1.0 mmol) and F-TEDA-BF_4 (264 mg, 0.75 mmol). Then, $MeNO_2$ (4.0 mL) was added via syringe, followed by styrene (57.2 µL, 0.5 mmol) and 1,3-dibromobenzene (60 µL, 0.5 mmol, internal standard). Time courses were monitored using GC by taking aliquots. The aliquots were quenched by filtering through a plug of sodium thiosulfate. The initial rates of the reactions were obtained from the reaction time courses.

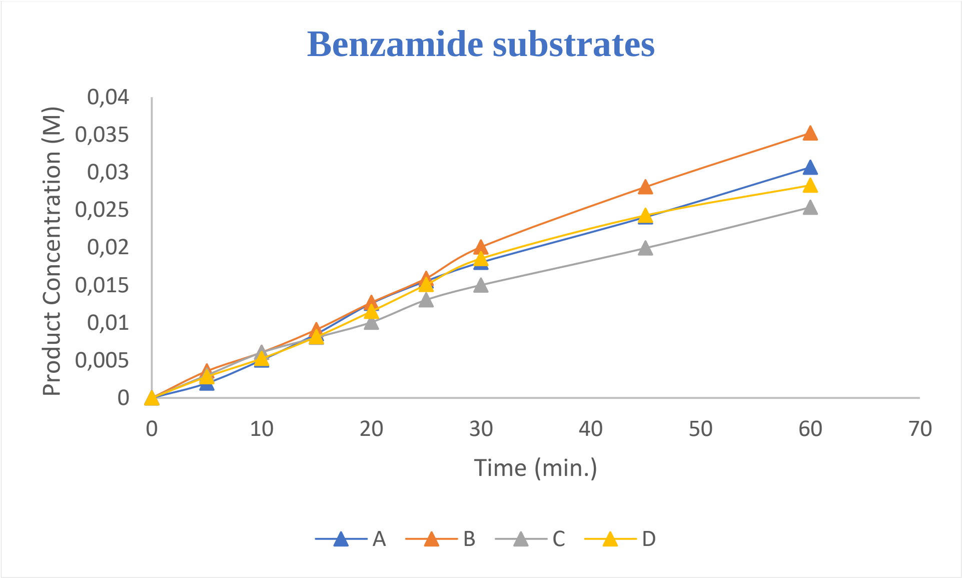

Figure S7: Initial rate study of standard reaction using different benzamide substrates where A = benzamide, B = *p*-methoxybenzamide, C = *p*-(trifluoromethyl)benzamide, D = *p*-bromobenzamide.

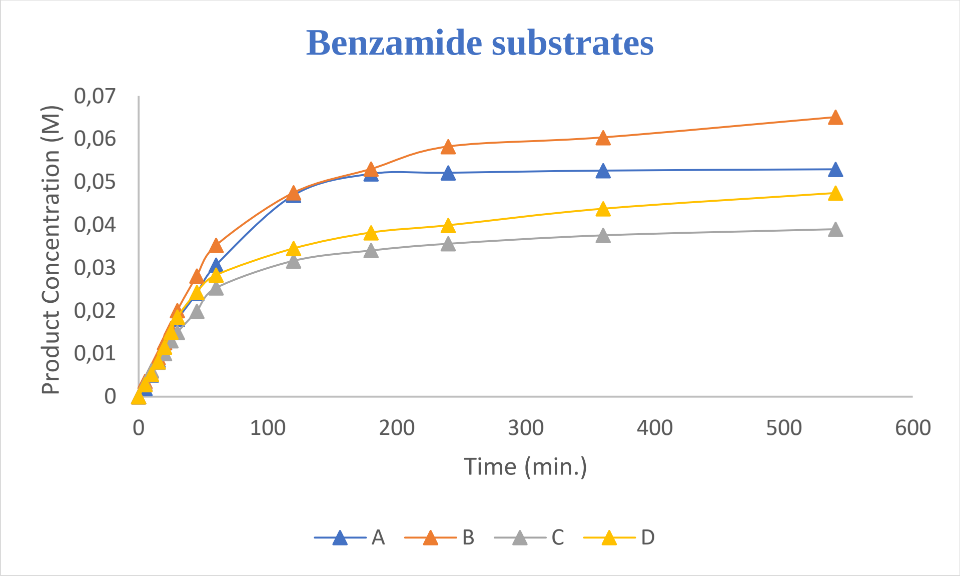

Figure S8: Overall rate study of standard reaction using different benzamide substrates where A = benzamide, B = *p*-methoxybenzamide, C = *p*-(trifluoromethyl)benzamide, D = *p*-bromobenzamide.

	Benzamide substrates				
S.No	Time	A	B	C	D
	min	M	M	M	M
1	0	0	0	0	0
2	5	0.002	0.0036	0.003	0.0028
3	10	0.005	0.0061	0.006	0.0052
4	15	0.0085	0.0091	0.008	0.0081
5	20	0.0126	0.0127	0.0101	0.0115
6	25	0.0155	0.0159	0.013	0.0151
7	30	0.018	0.0201	0.015	0.0186
8	45	0.0241	0.0281	0.0199	0.0243
9	60	0.0307	0.0352	0.0253	0.0283
10	120	0.0469	0.0475	0.0316	0.0345
11	180	0.0519	0.053	0.034	0.0382
12	240	0.0521	0.0582	0.0356	0.0399
13	360	0.0526	0.0604	0.0376	0.0437
14	540	0.0529	0.0651	0.039	0.0474

Table S4. Raw data for gas chromatography time courses. A = benzamide, B = *p*-methoxybenzamide, C = *p*-(trifluoromethyl)benzamide, D = *p*-bromobenzamide.

5. References:

1. Wu, F., Kaur, N., Alom, N. E.; Li, W. *J. Am. Chem. Soc. Au*, **2021**, *1*, 734-741.

2. Zhong, C. L., Tang, B. Yin, P., Chen, Y.; He, L. *J. Org. Chem*. **2012**, *77*, 4271-4277.

3. Wang, Y. Z., Liang, P. Y., Liu, H. C., Lin, W. J., Zhou, P. P.; Yu, W. *Org. Lett*, **2022**, *24*, 6037-6042.

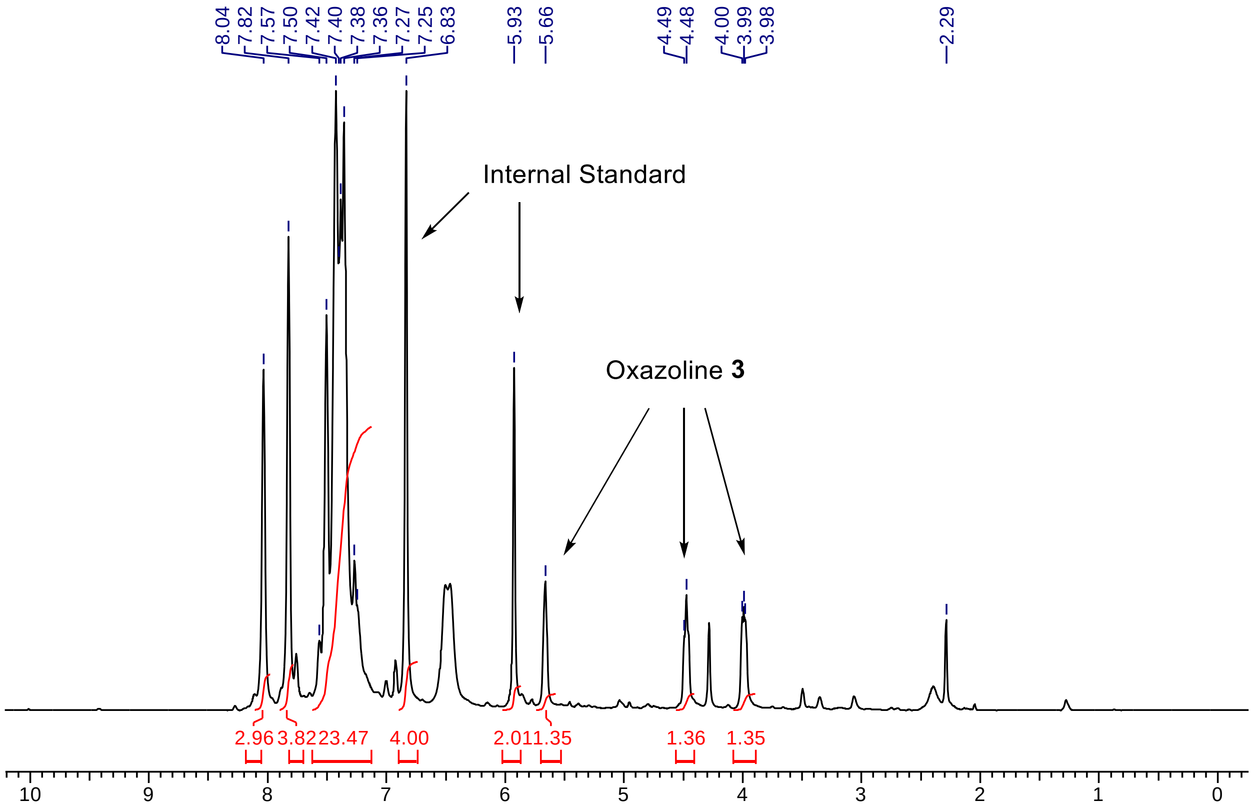

Figure S9: Crude reaction NMR for entry 7 of Table 1 of the main text. 1,3-benzodioxole (14.8 mg) was used as an internal standard to calculate NMR yields. Oxazoline **3** and the internal standard are labeled with arrows.

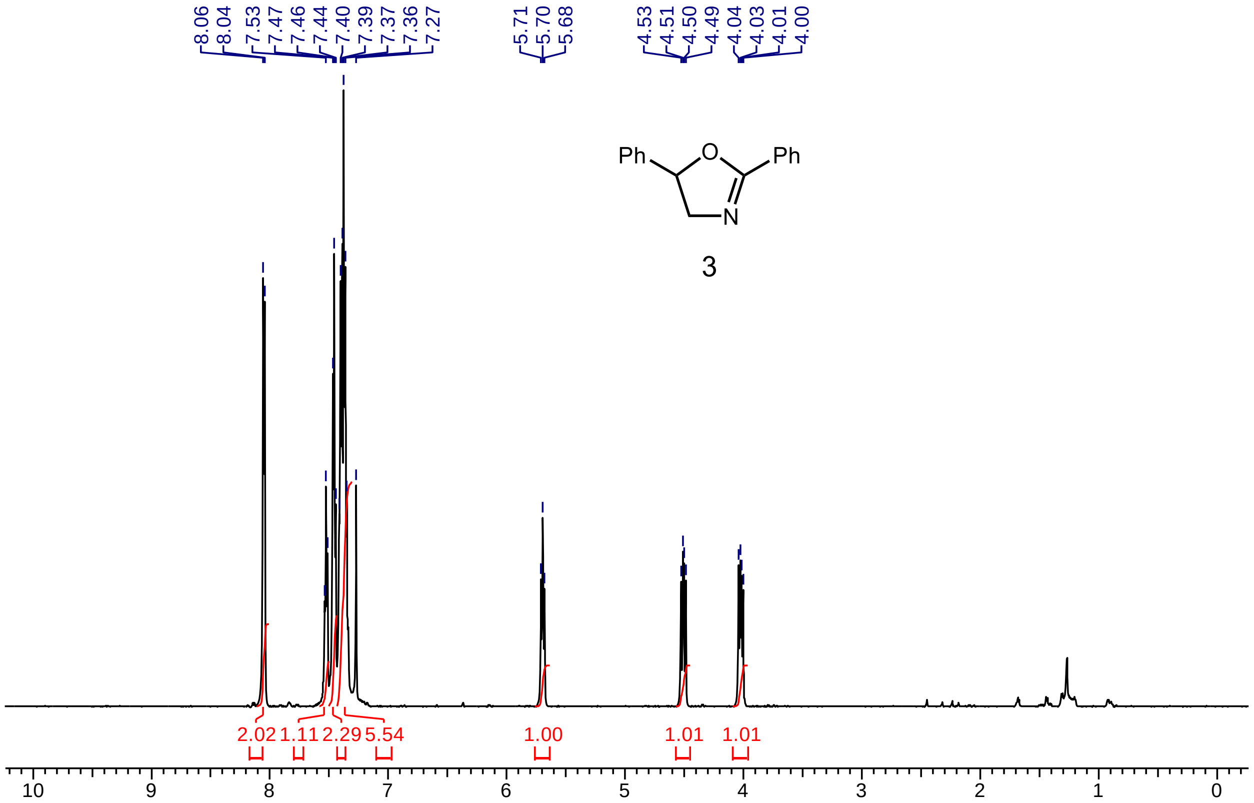
8.06
8.04
7.53
7.47
7.46
7.44
7.40
7.39
7.37
7.36
7.27
5.71
5.70
5.68
4.53
4.51
4.50
4.49
4.04
4.03
4.01
4.00
Ph
O
Ph
N
3
2.02
1.11
2.29
5.54
1.00
1.01
1.01
10
9
8
7
6
5
4
3
2
1
0

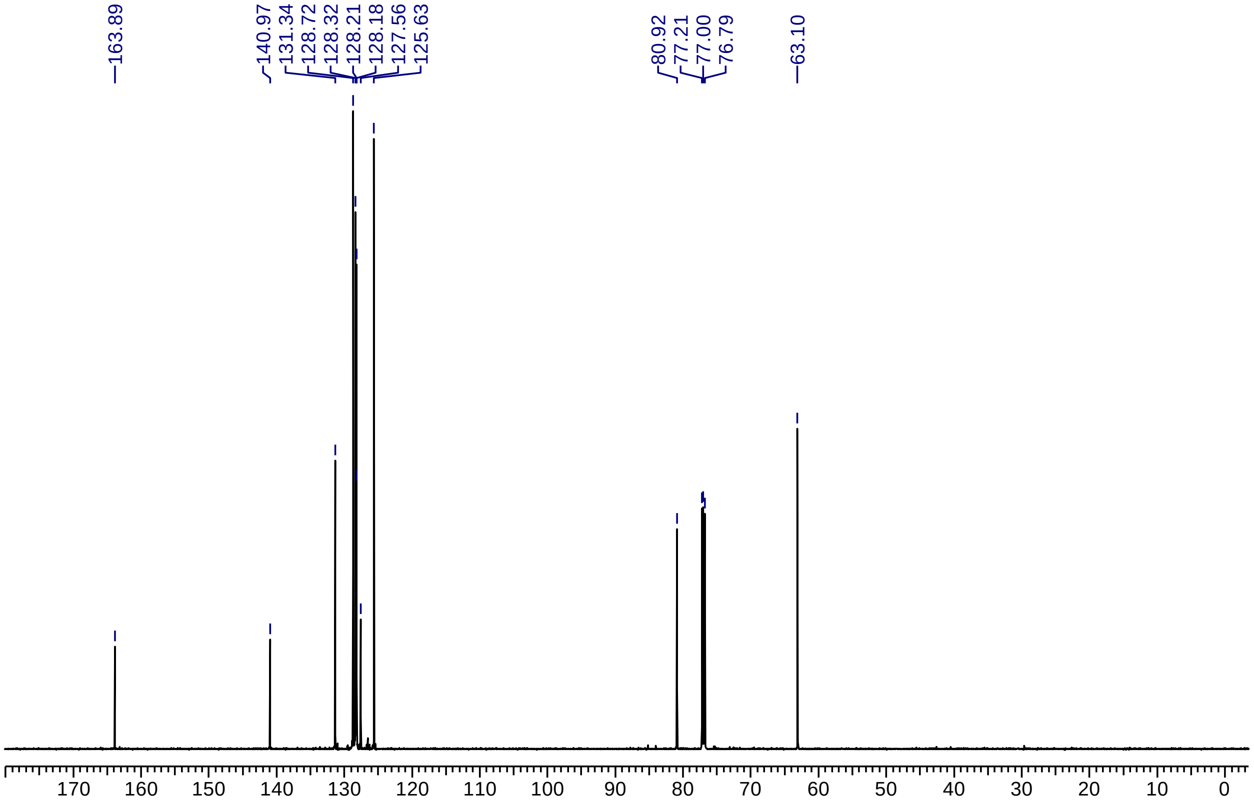
163.89
140.97
131.34
128.72
128.32
128.21
128.18
127.56
125.63
80.92
77.21
77.00
76.79
63.10
170
160
150
140
130
120
110
100
90
80
70
60
50
40
30
20
10
0

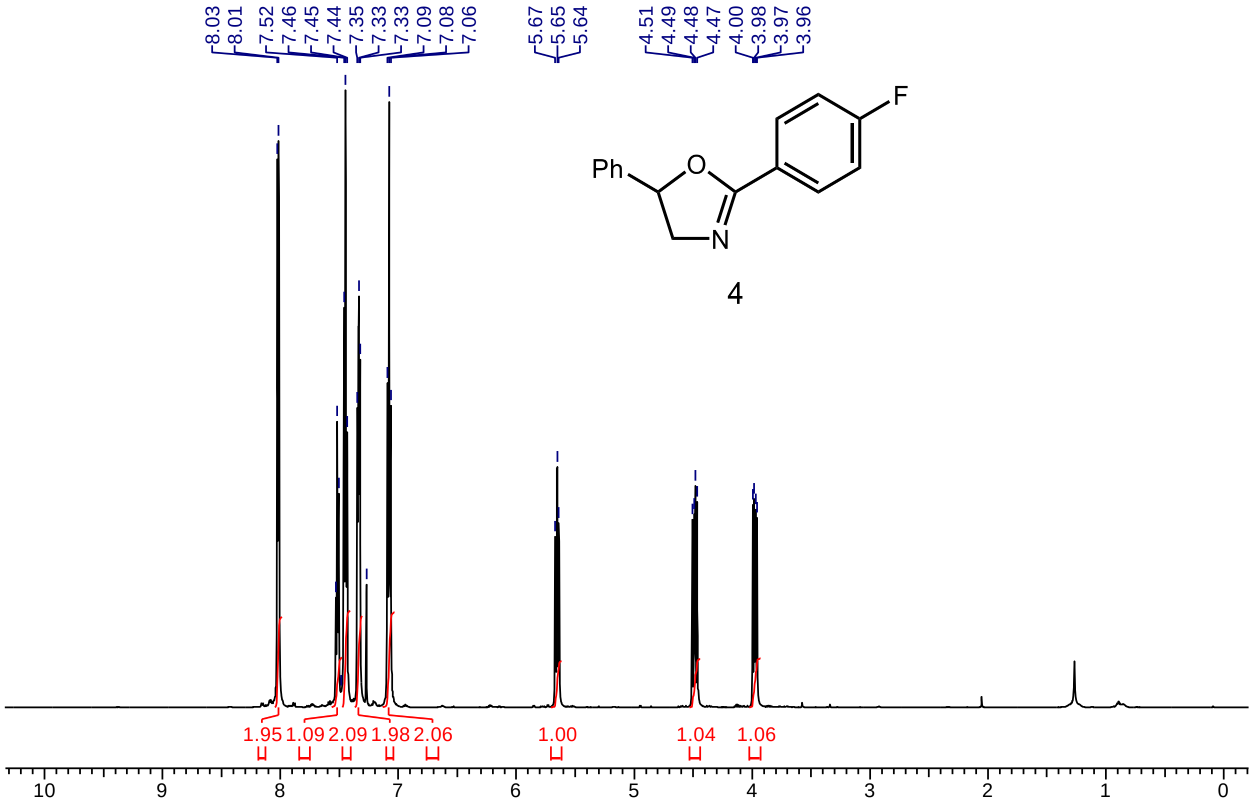
8.03
8.01
7.52
7.46
7.45
7.44
7.35
7.33
7.33
7.09
7.08
7.06
5.67
5.65
5.64
4.51
4.49
4.48
4.47
4.00
3.98
3.97
3.96
Ph
O
N
F
4
1.95
1.09
2.09
1.98
2.06
1.00
1.04
1.06
10
9
8
7
6
5
4
3
2
1
0

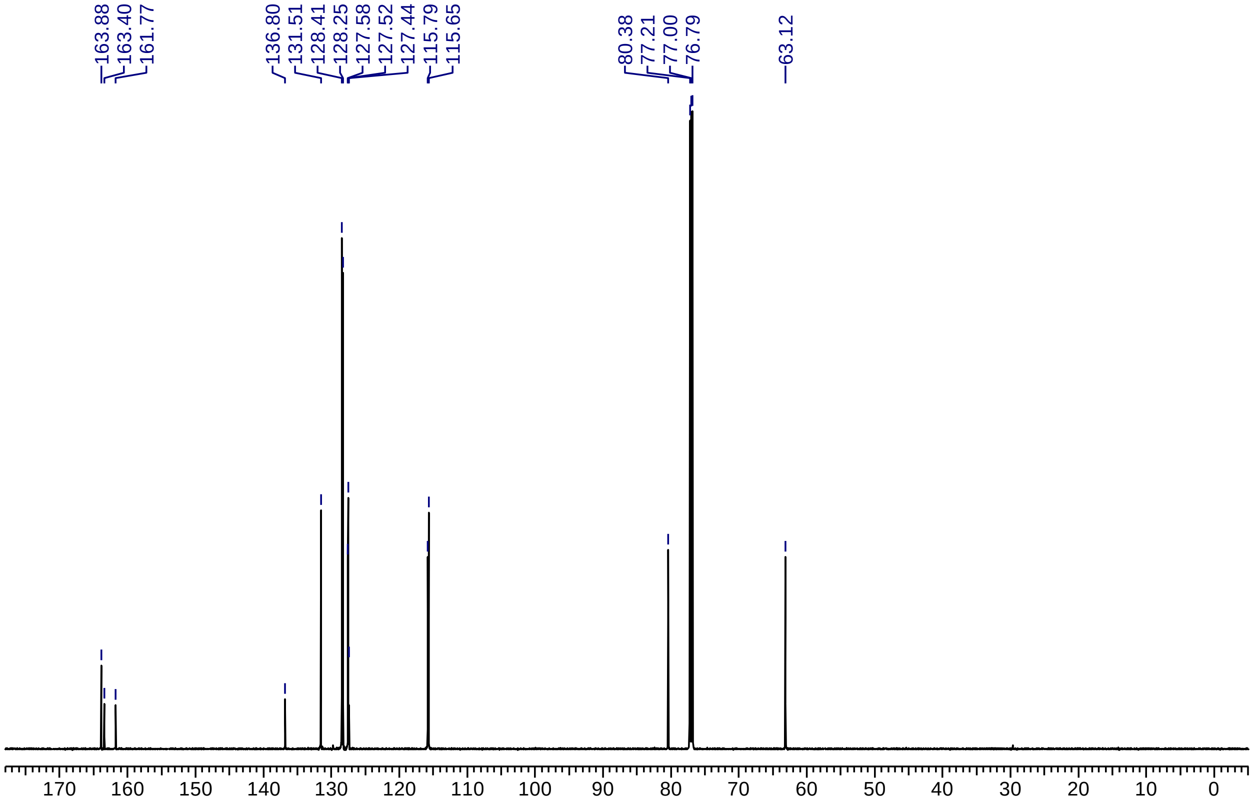
163.88
163.40
161.77
136.80
131.51
128.41
128.25
127.58
127.52
127.44
115.79
115.65
80.38
77.21
77.00
76.79
63.12
170
160
150
140
130
120
110
100
90
80
70
60
50
40
30
20
10
0

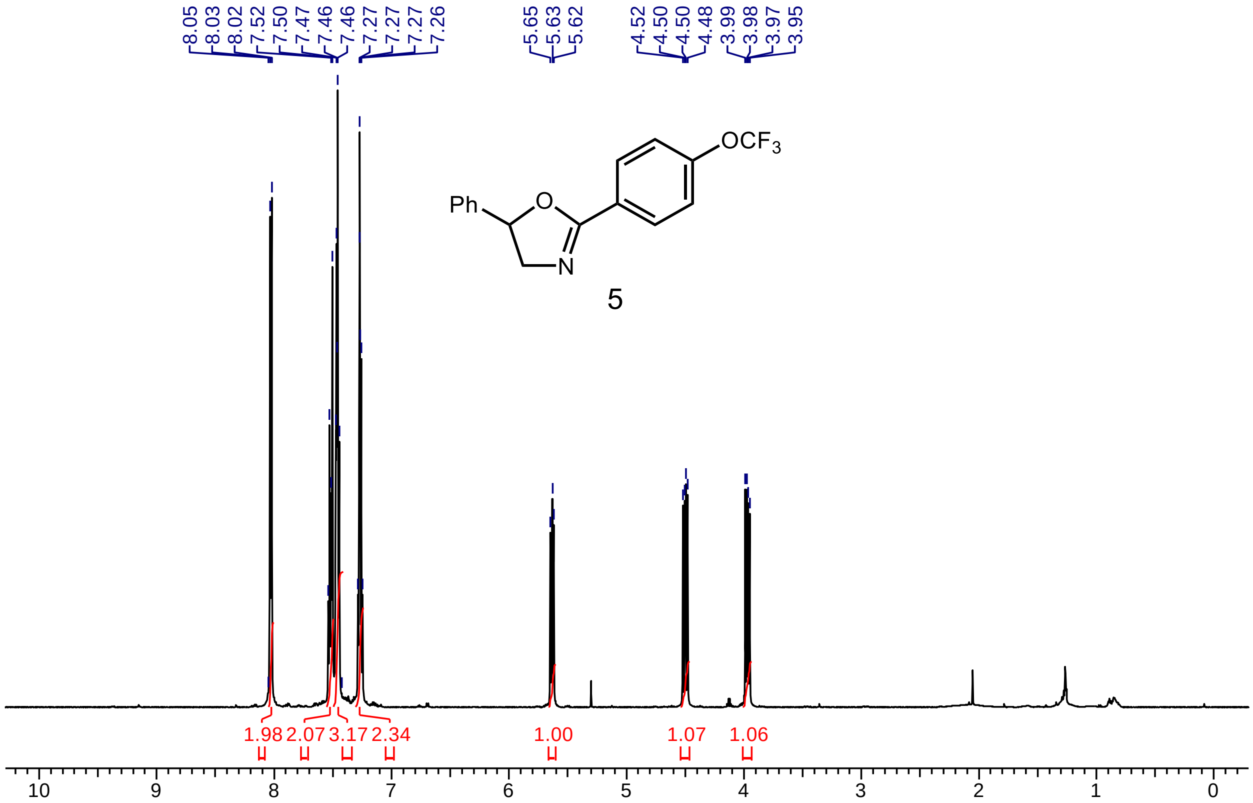
8.05
8.03
8.02
7.52
7.50
7.47
7.46
7.46
7.27
7.27
7.27
7.26
5.65
5.63
5.62
4.52
4.50
4.50
4.48
3.99
3.98
3.97
3.95
OCF3
Ph
O
N
5
1.98
2.07
3.17
2.34
1.00
1.07
1.06

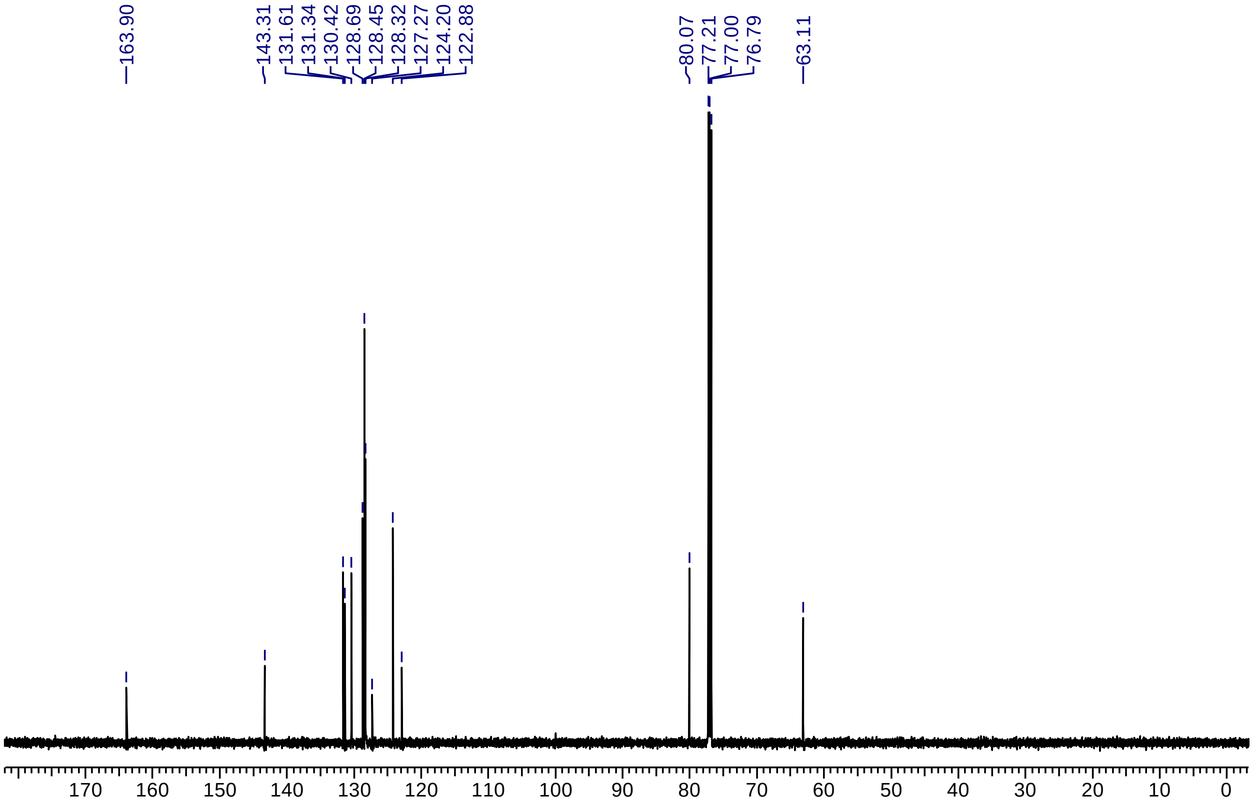
163.90
143.31
131.61
131.34
130.42
128.69
128.45
128.32
127.27
124.20
122.88
80.07
77.21
77.00
76.79
63.11

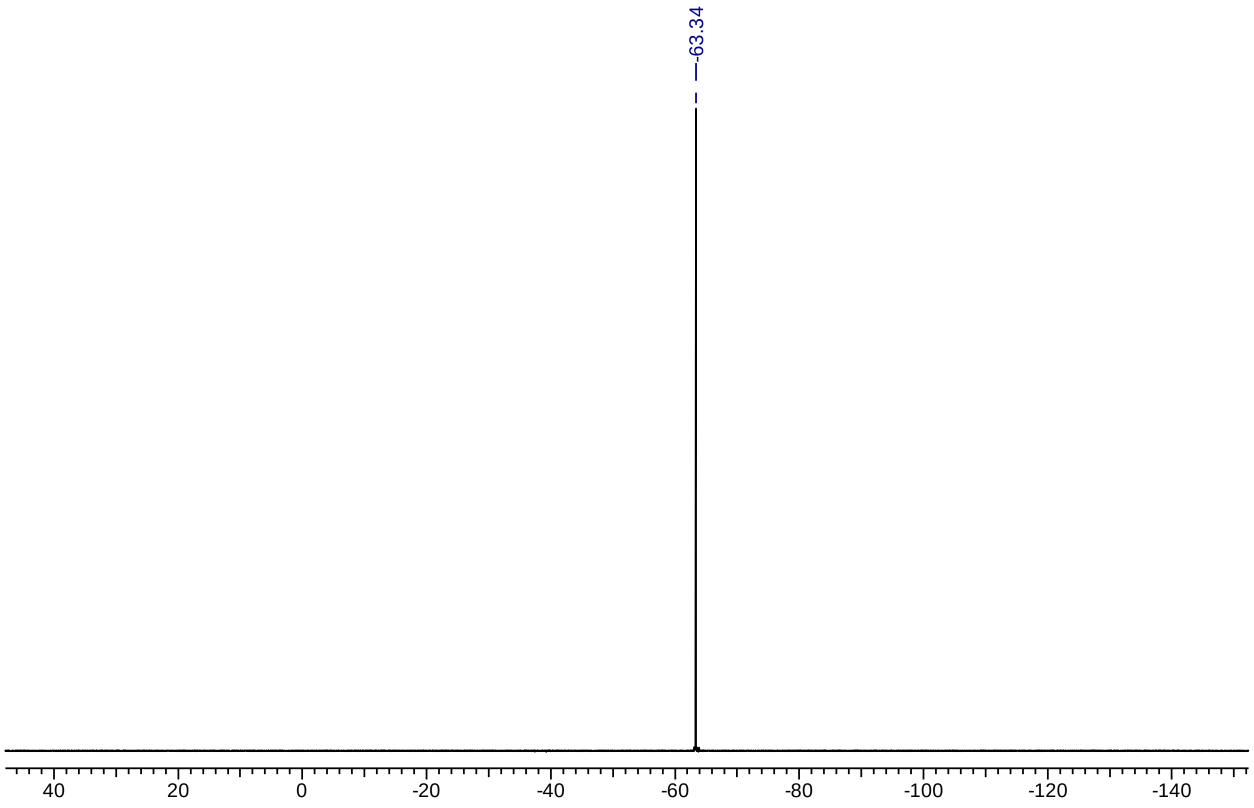
-63.34
40
20
0
-20
-40
-60
-80
-100
-120
-140

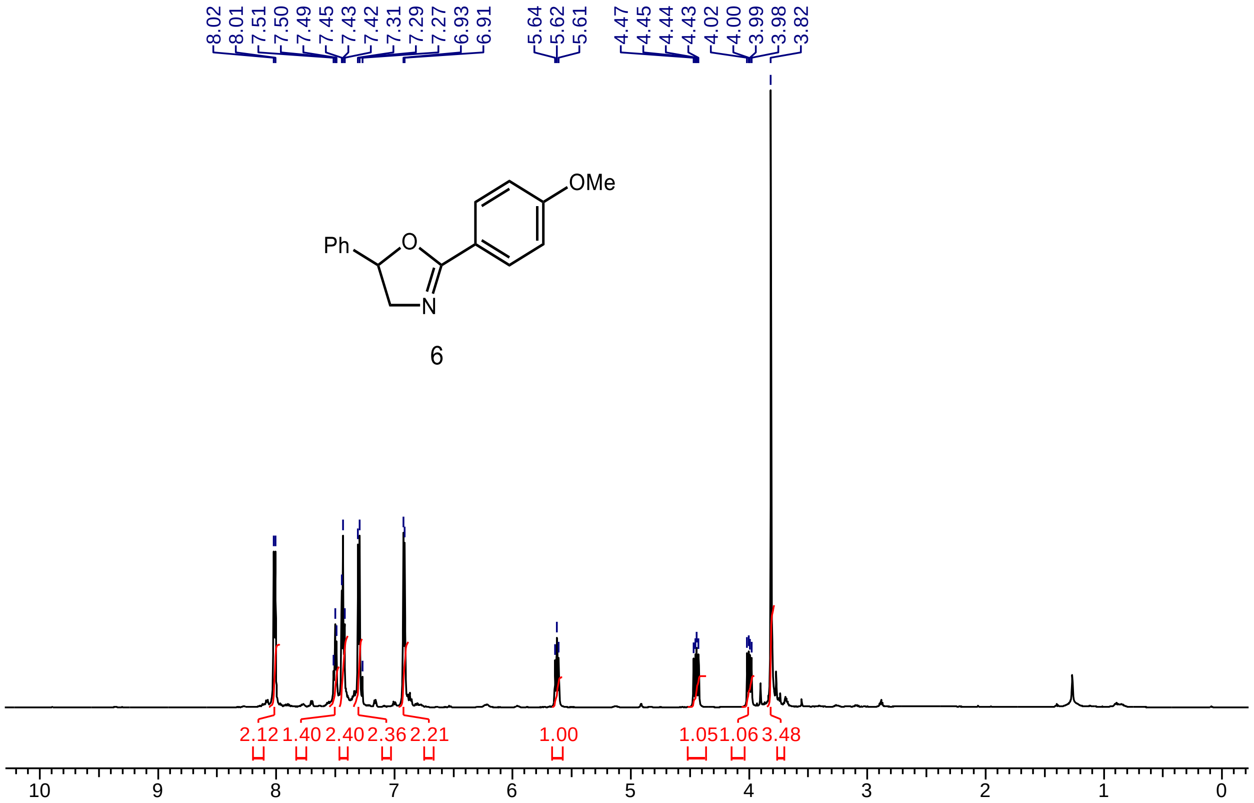
8.02
8.01
7.51
7.50
7.49
7.45
7.43
7.42
7.31
7.29
7.27
6.93
6.91
5.64
5.62
5.61
4.47
4.45
4.44
4.43
4.02
4.00
3.99
3.98
3.82
OMe
Ph
O
N
6
2.12
1.40
2.40
2.36
2.21
1.00
1.05
1.06
3.48
10 9 8 7 6 5 4 3 2 1 0

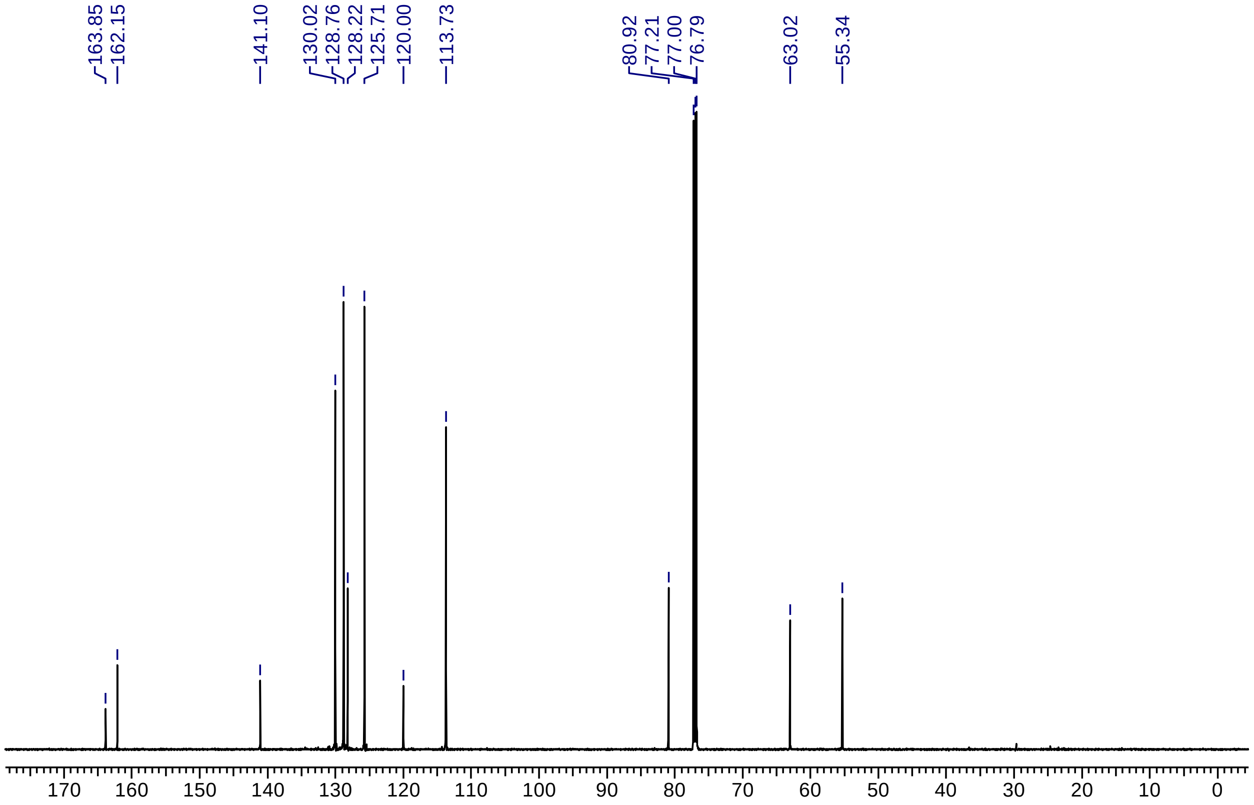
163.85
162.15
141.10
130.02
128.76
128.22
125.71
120.00
113.73
80.92
77.21
77.00
76.79
63.02
55.34
170 160 150 140 130 120 110 100 90 80 70 60 50 40 30 20 10 0

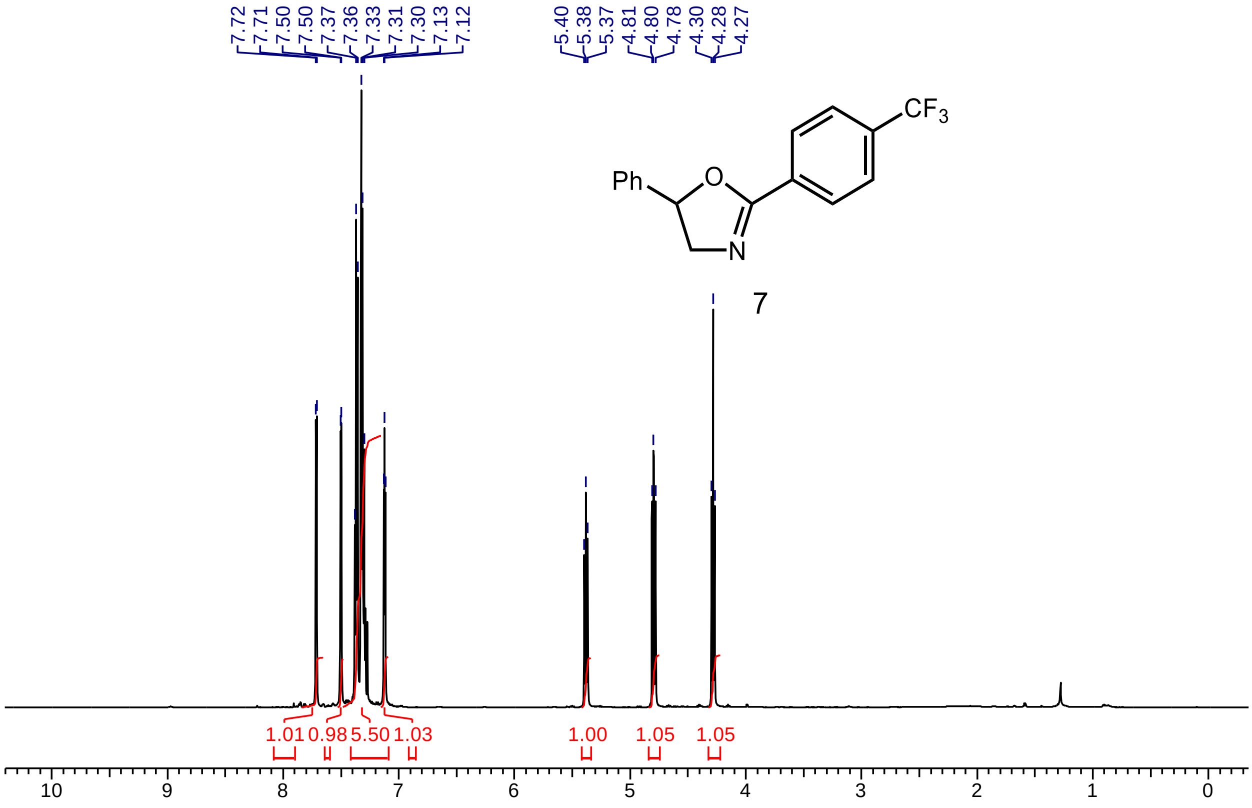
7.72
7.71
7.50
7.50
7.37
7.36
7.33
7.31
7.30
7.13
7.12
5.40
5.38
5.37
4.81
4.80
4.78
4.30
4.28
4.27
Ph
O
N
CF3
7
1.01
0.98
5.50
1.03
1.00
1.05
1.05
10
9
8
7
6
5
4
3
2
1
0

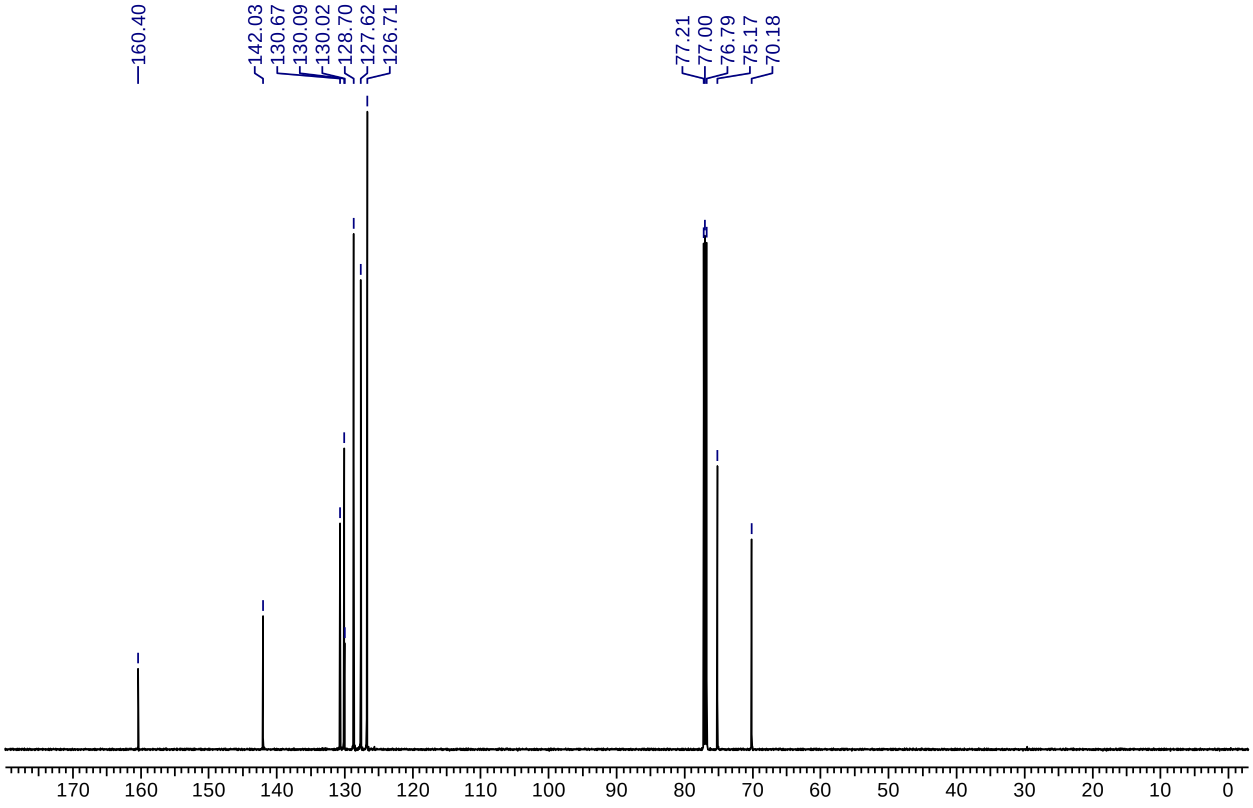
160.40
142.03
130.67
130.09
130.02
128.70
127.62
126.71
77.21
77.00
76.79
75.17
70.18
170
160
150
140
130
120
110
100
90
80
70
60
50
40
30
20
10
0

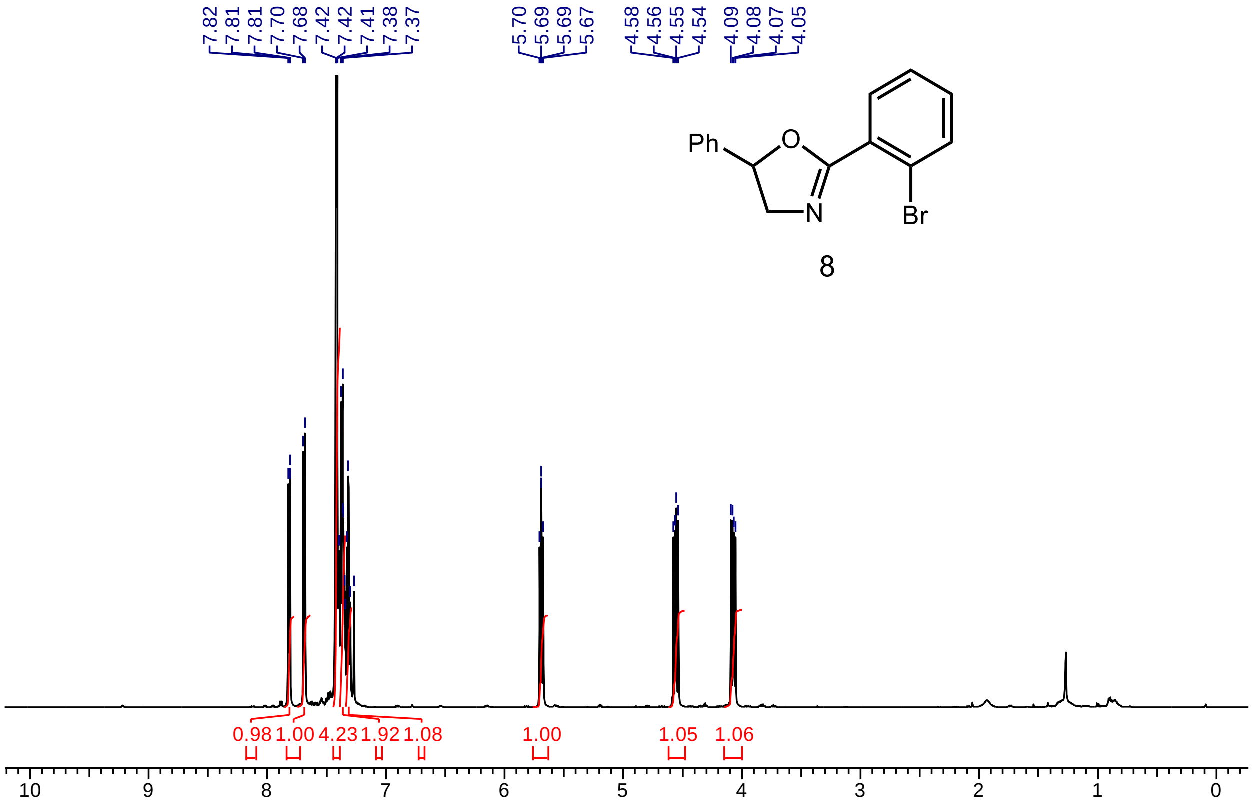
7.82
7.81
7.81
7.70
7.68
7.42
7.42
7.41
7.38
7.37
5.70
5.69
5.69
5.67
4.58
4.56
4.55
4.54
4.09
4.08
4.07
4.05
Ph
O
N
Br
8
0.98
1.00
4.23
1.92
1.08
1.00
1.05
1.06
10
9
8
7
6
5
4
3
2
1
0

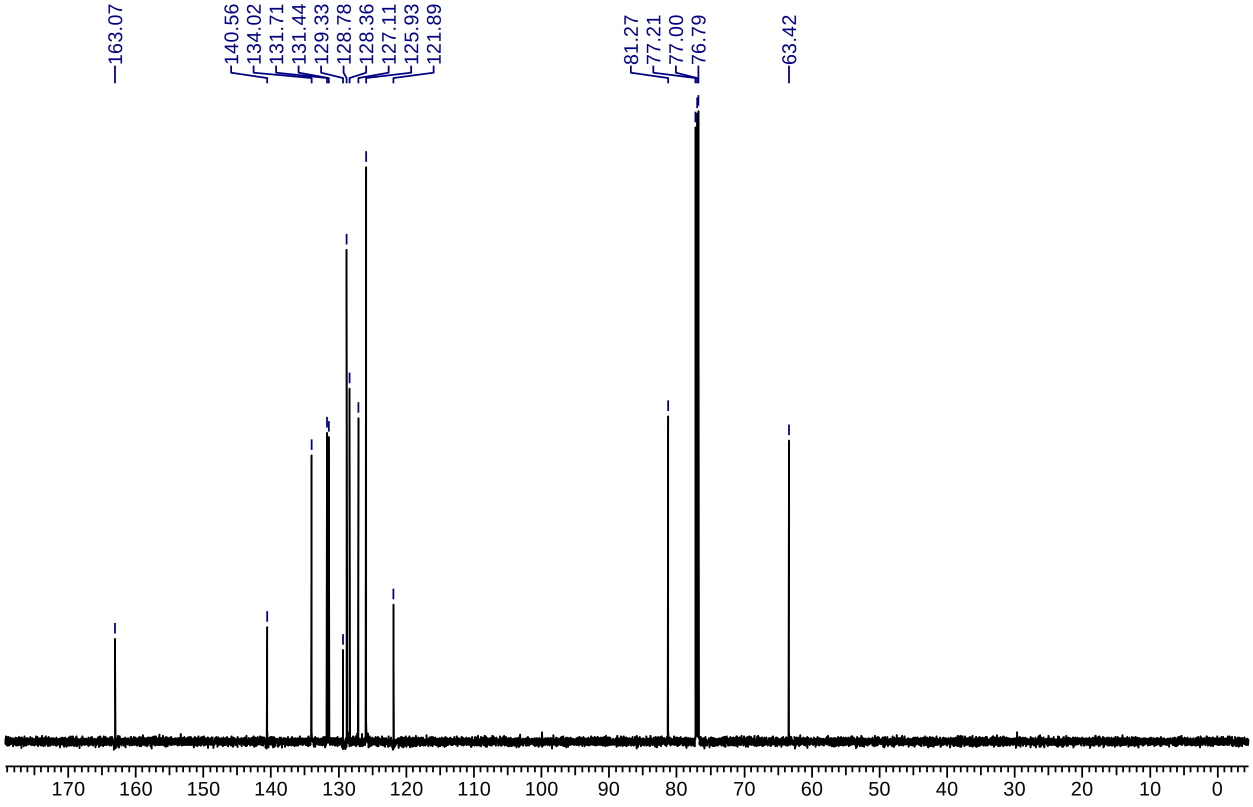
163.07
140.56
134.02
131.71
131.44
129.33
128.78
128.36
127.11
125.93
121.89
81.27
77.21
77.00
76.79
63.42
170
160
150
140
130
120
110
100
90
80
70
60
50
40
30
20
10
0

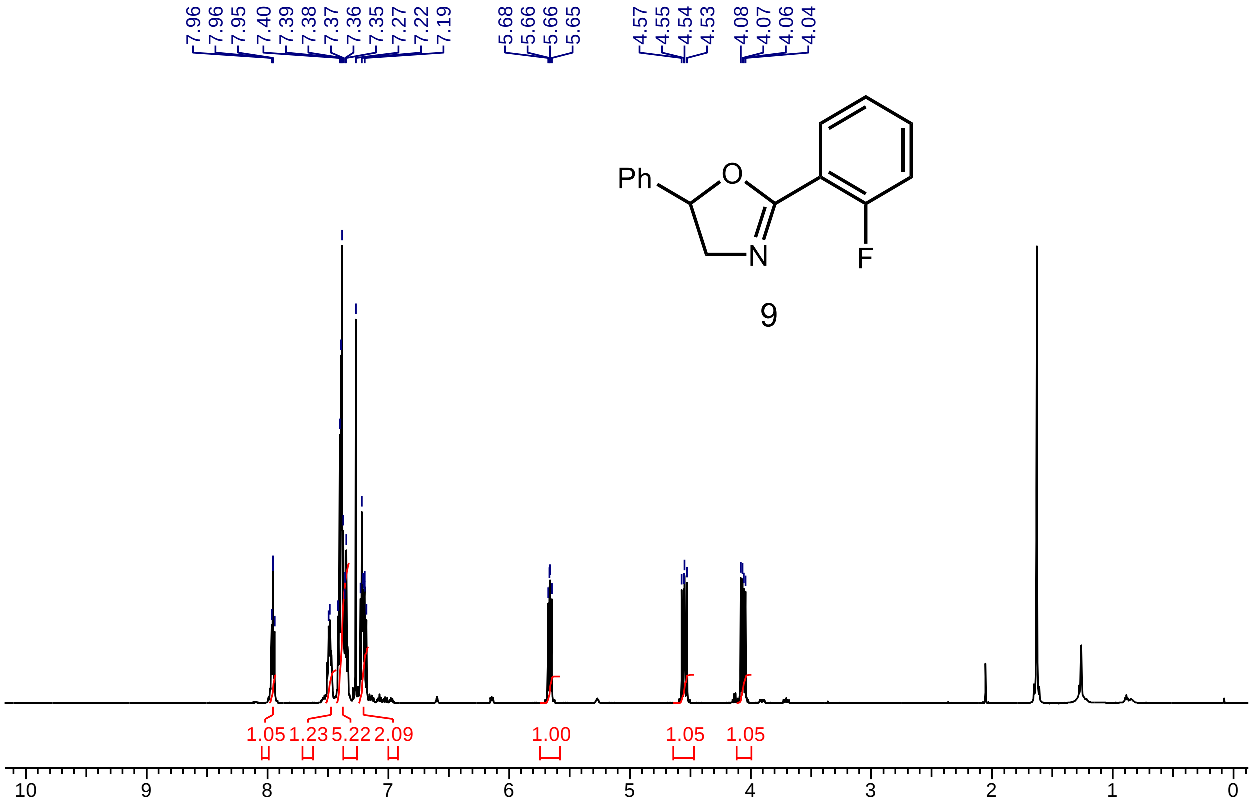
7.96
7.96
7.95
7.40
7.39
7.38
7.37
7.36
7.35
7.27
7.22
7.19
5.68
5.66
5.66
5.65
4.57
4.55
4.54
4.53
4.08
4.07
4.06
4.04
Ph
O
N
F
9
1.05
1.23
5.22
2.09
1.00
1.05
1.05
10
9
8
7
6
5
4
3
2
1
0

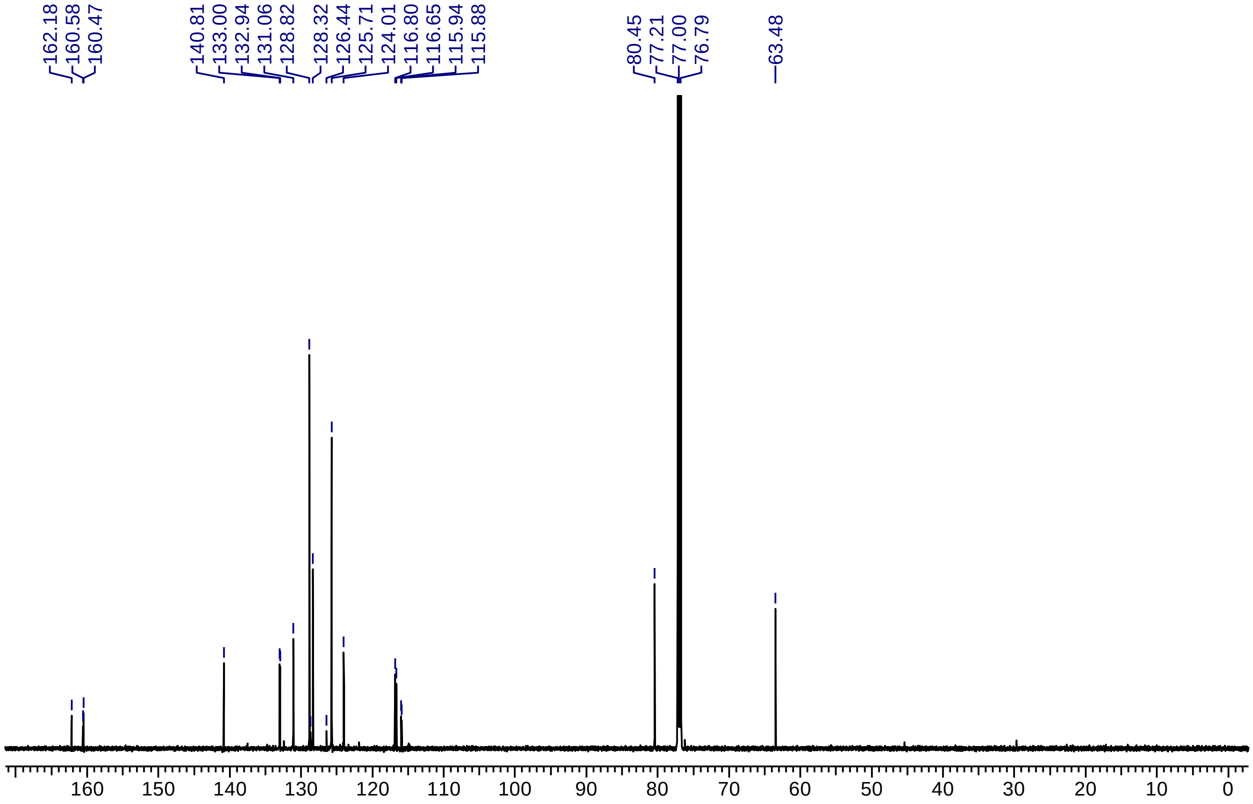
162.18
160.58
160.47
140.81
133.00
132.94
131.06
128.82
128.32
126.44
125.71
124.01
116.80
116.65
115.94
115.88
80.45
77.21
77.00
76.79
63.48
160
150
140
130
120
110
100
90
80
70
60
50
40
30
20
10
0

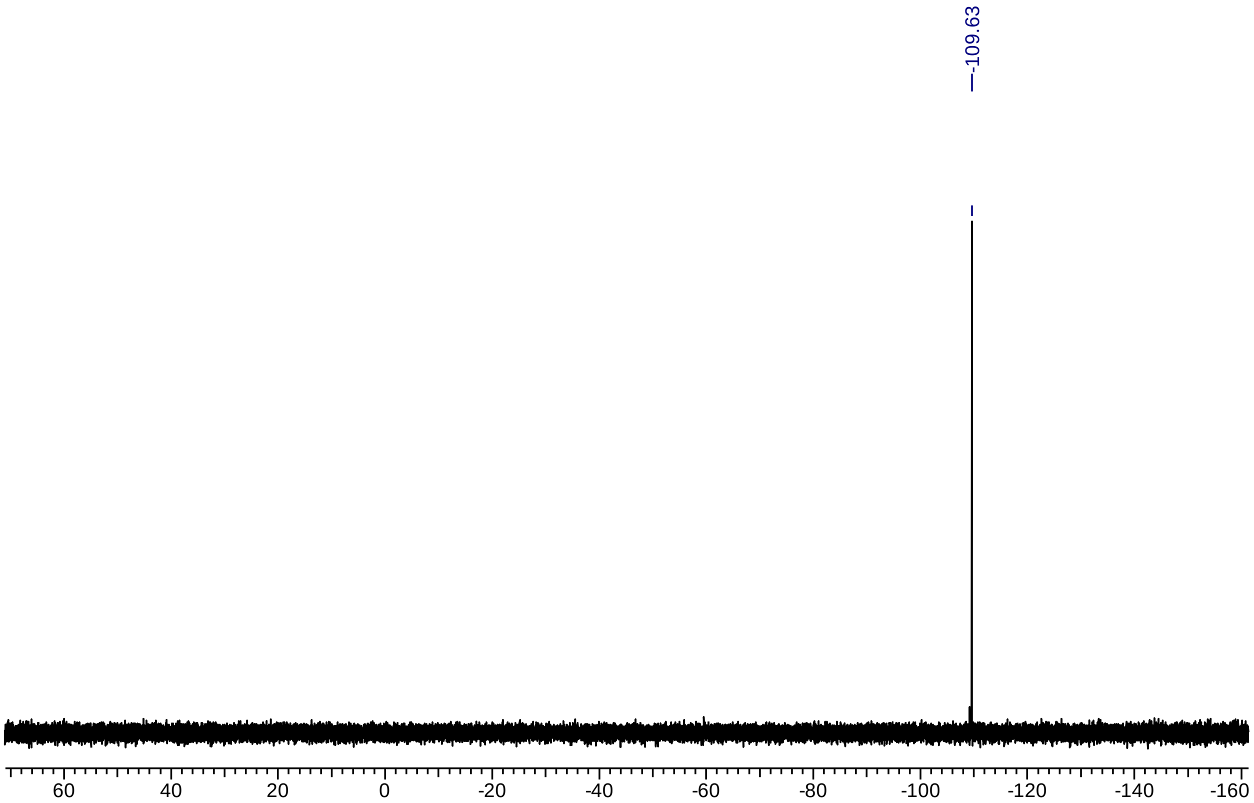

-109.63
60
40
20
0
-20
-40
-60
-80
-100
-120
-140
-160

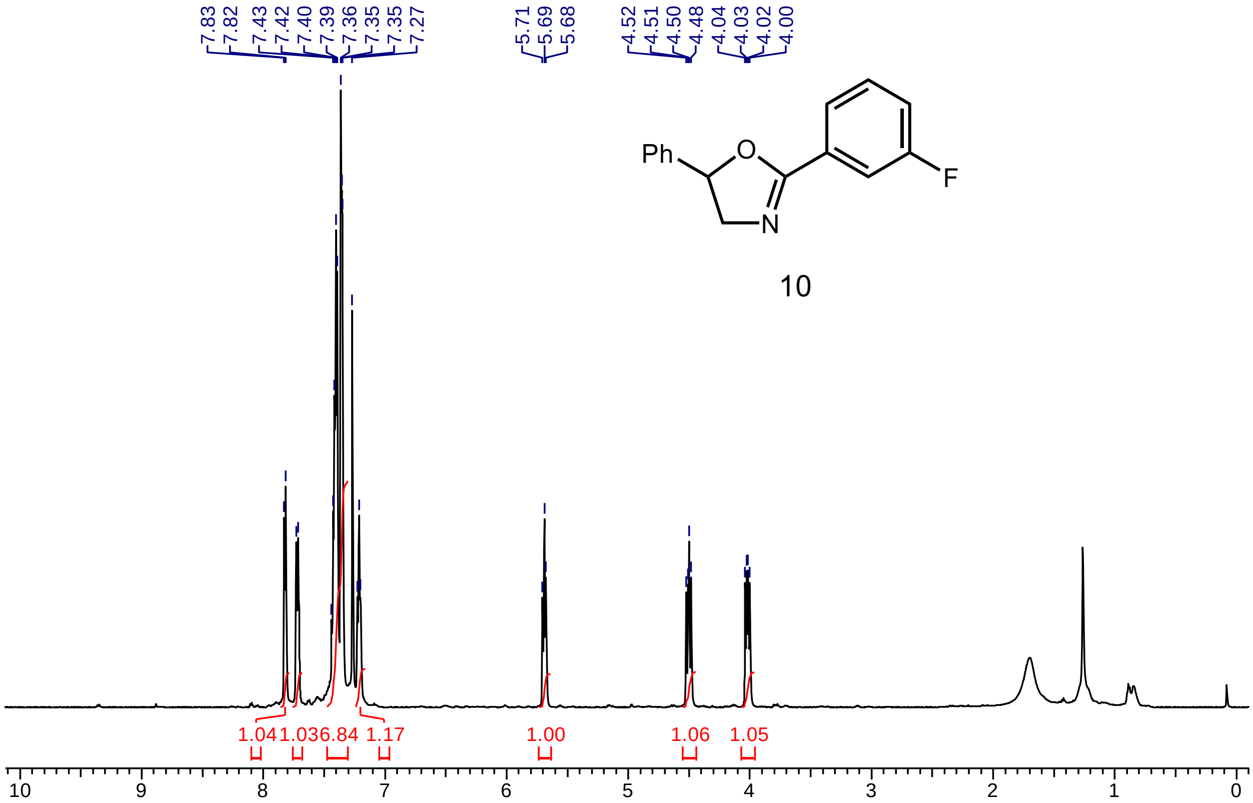
7.83
7.82
7.43
7.42
7.40
7.39
7.36
7.35
7.35
7.27
5.71
5.69
5.68
4.52
4.51
4.50
4.48
4.04
4.03
4.02
4.00
Ph
O
N
F
1.04
1.03
6.84
1.17
1.00
1.06
1.05
10 9 8 7 6 5 4 3 2 1 0

10

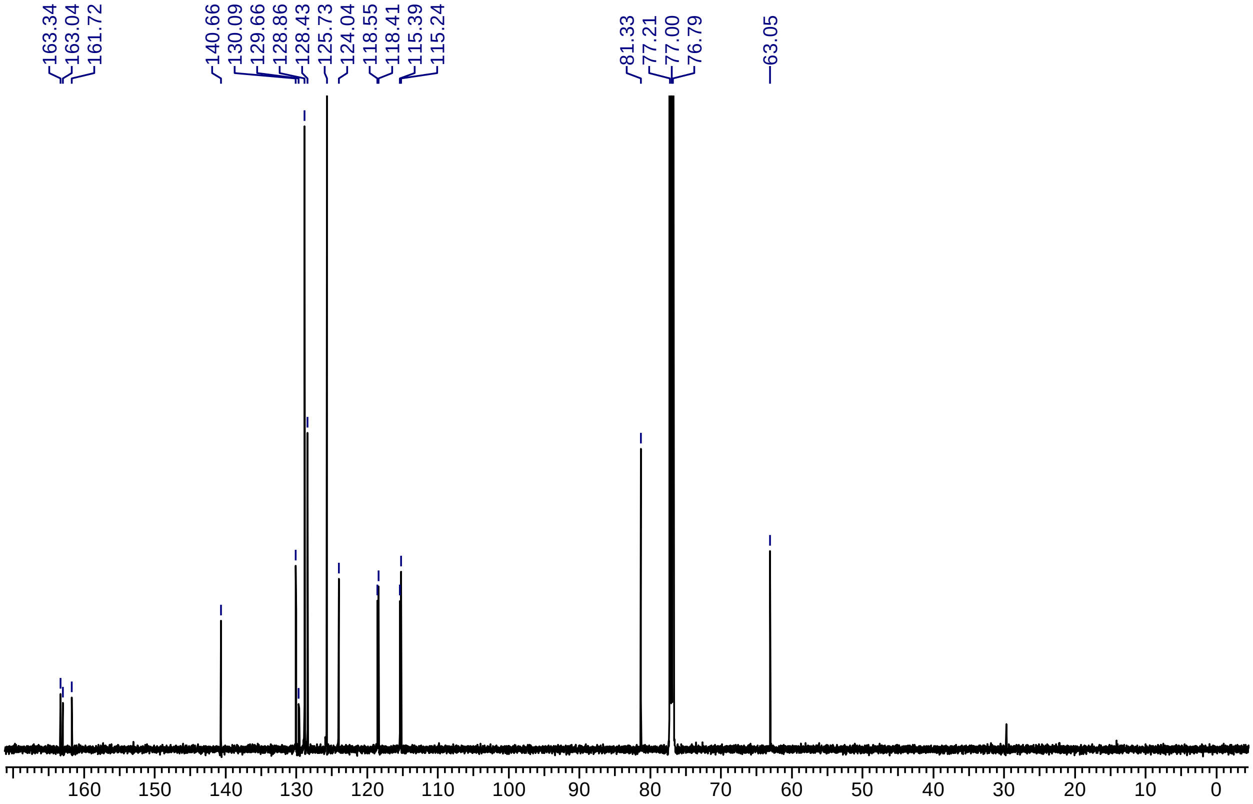
163.34
163.04
161.72
140.66
130.09
129.66
128.86
128.43
125.73
124.04
118.55
118.41
115.39
115.24
81.33
77.21
77.00
76.79
63.05
160 150 140 130 120 110 100 90 80 70 60 50 40 30 20 10 0

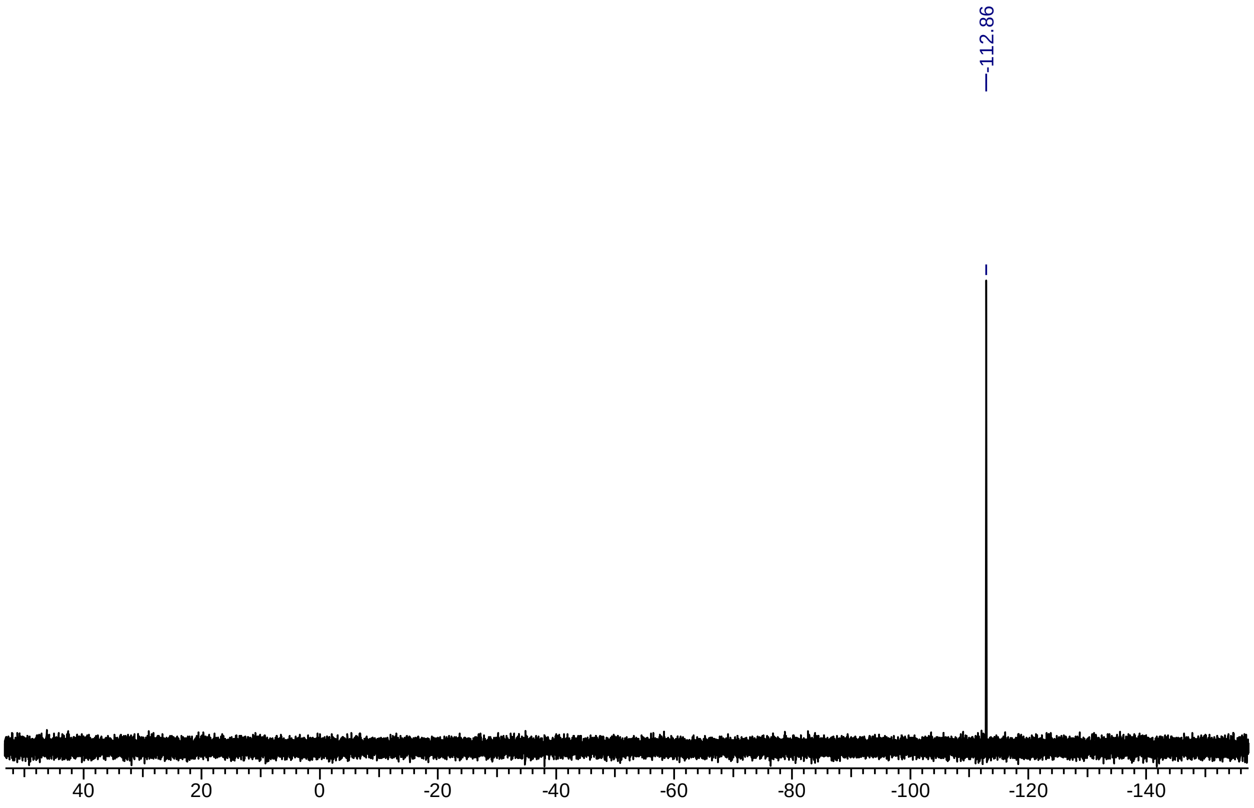
-112.86
40
20
0
-20
-40
-60
-80
-100
-120
-140

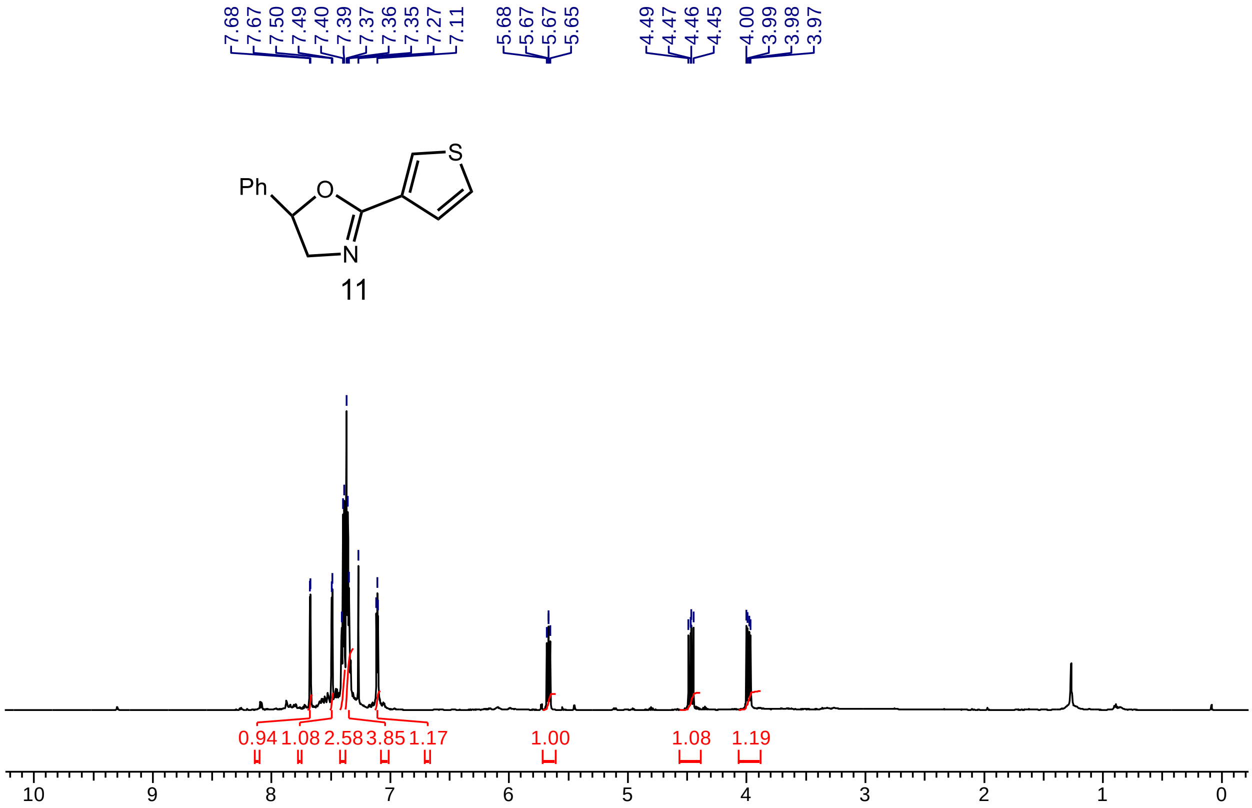
7.68
7.67
7.50
7.49
7.40
7.39
7.37
7.36
7.35
7.27
7.11
5.68
5.67
5.67
5.65
4.49
4.47
4.46
4.45
4.00
3.99
3.98
3.97
Ph
O
N
S
11
0.94
1.08
2.58
3.85
1.17
1.00
1.08
1.19
10
9
8
7
6
5
4
3
2
1
0

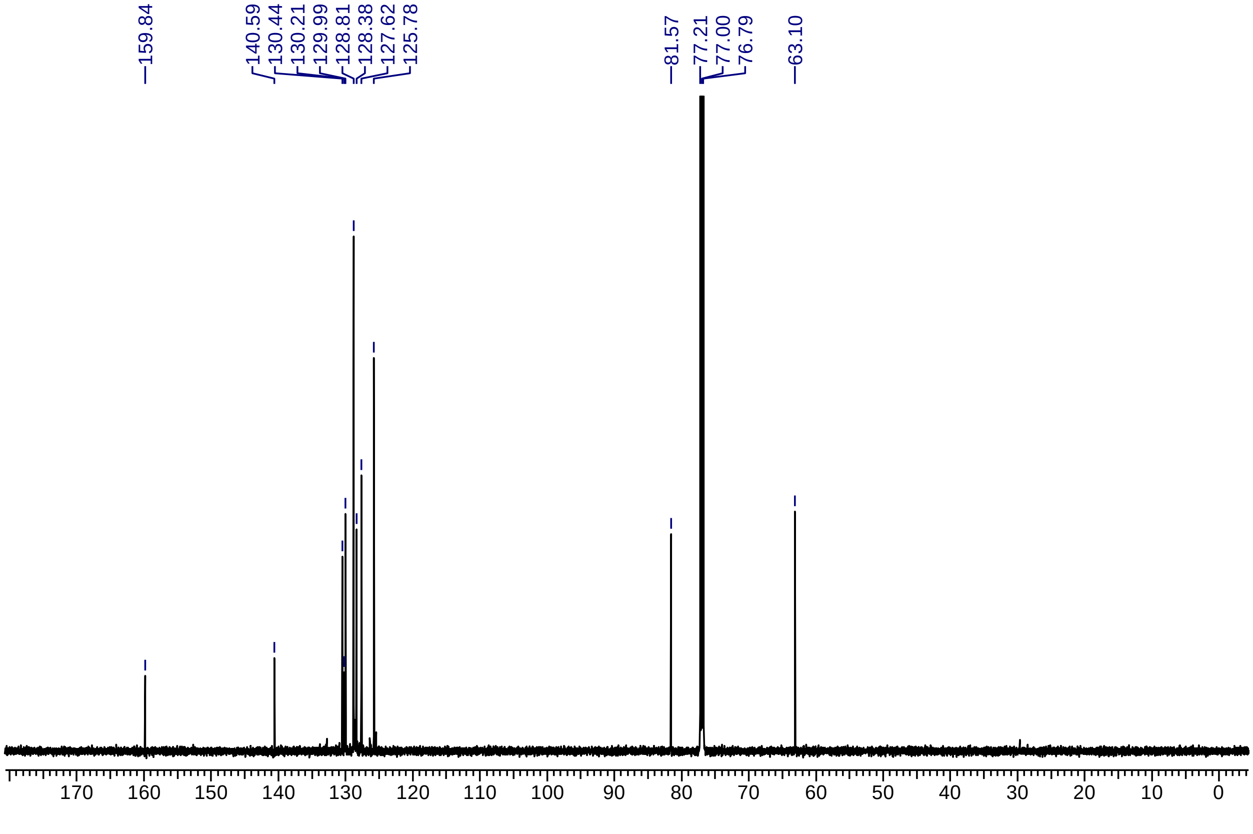
159.84
140.59
130.44
130.21
129.99
128.81
128.38
127.62
125.78
81.57
77.21
77.00
76.79
63.10
170
160
150
140
130
120
110
100
90
80
70
60
50
40
30
20
10
0

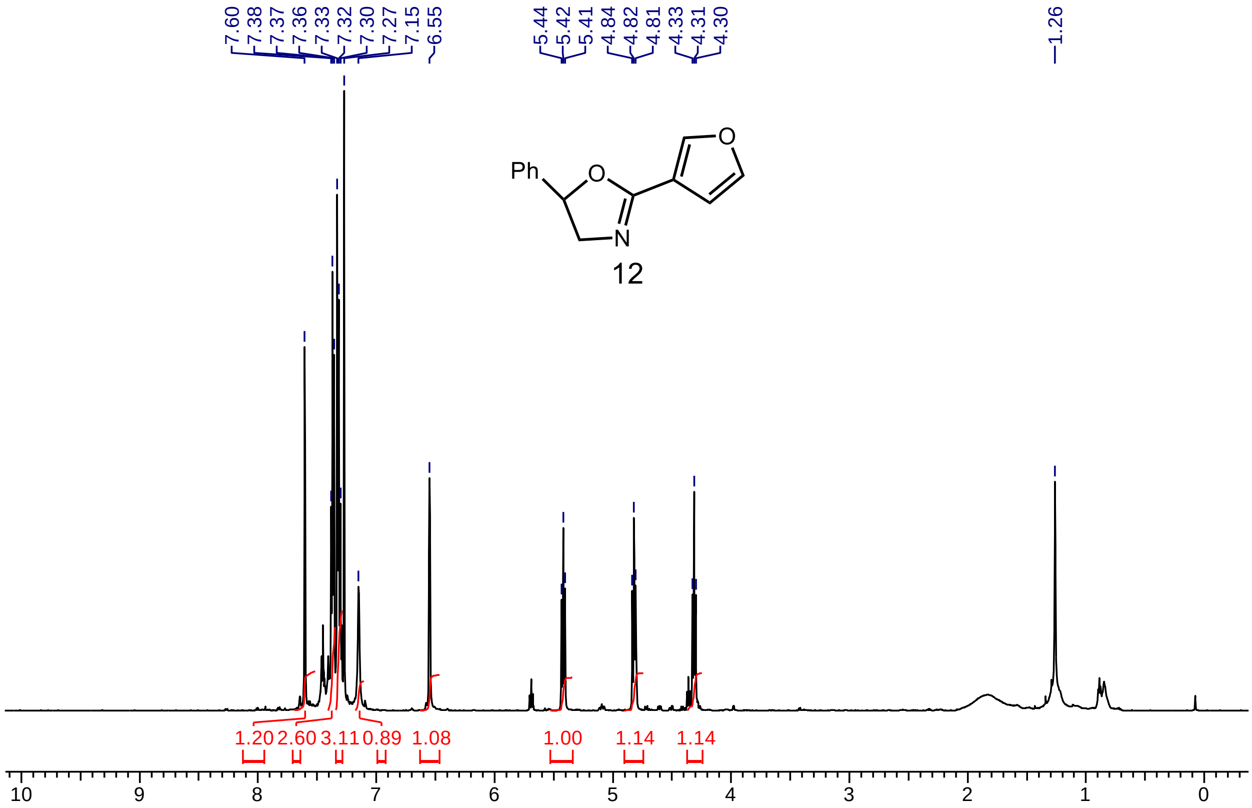
7.60
7.38
7.37
7.36
7.33
7.32
7.30
7.27
7.15
6.55
5.44
5.42
5.41
4.84
4.82
4.81
4.33
4.31
4.30
1.26
Ph
O
N
O
12
1.20
2.60
3.11
0.89
1.08
1.00
1.14
1.14
10
9
8
7
6
5
4
3
2
1
0

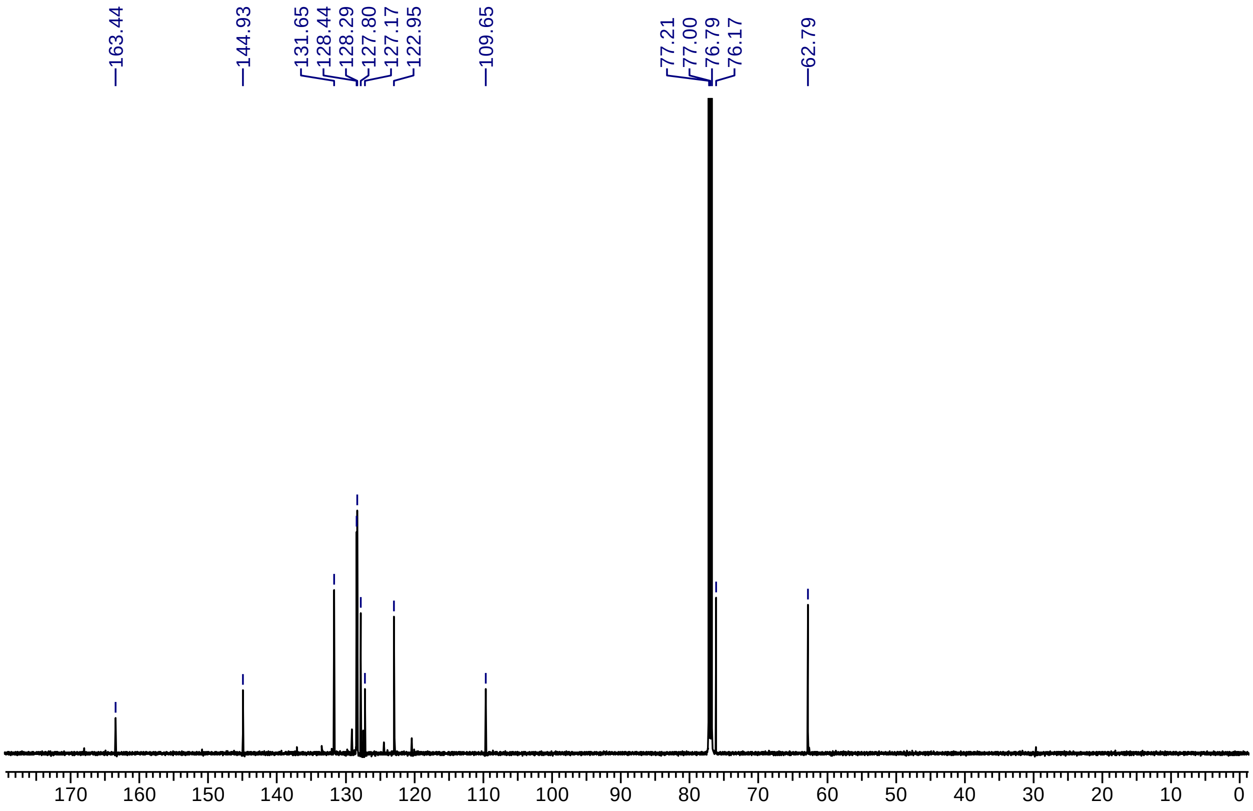
163.44
144.93
131.65
128.44
128.29
127.80
127.17
122.95
109.65
77.21
77.00
76.79
76.17
62.79
170
160
150
140
130
120
110
100
90
80
70
60
50
40
30
20
10
0

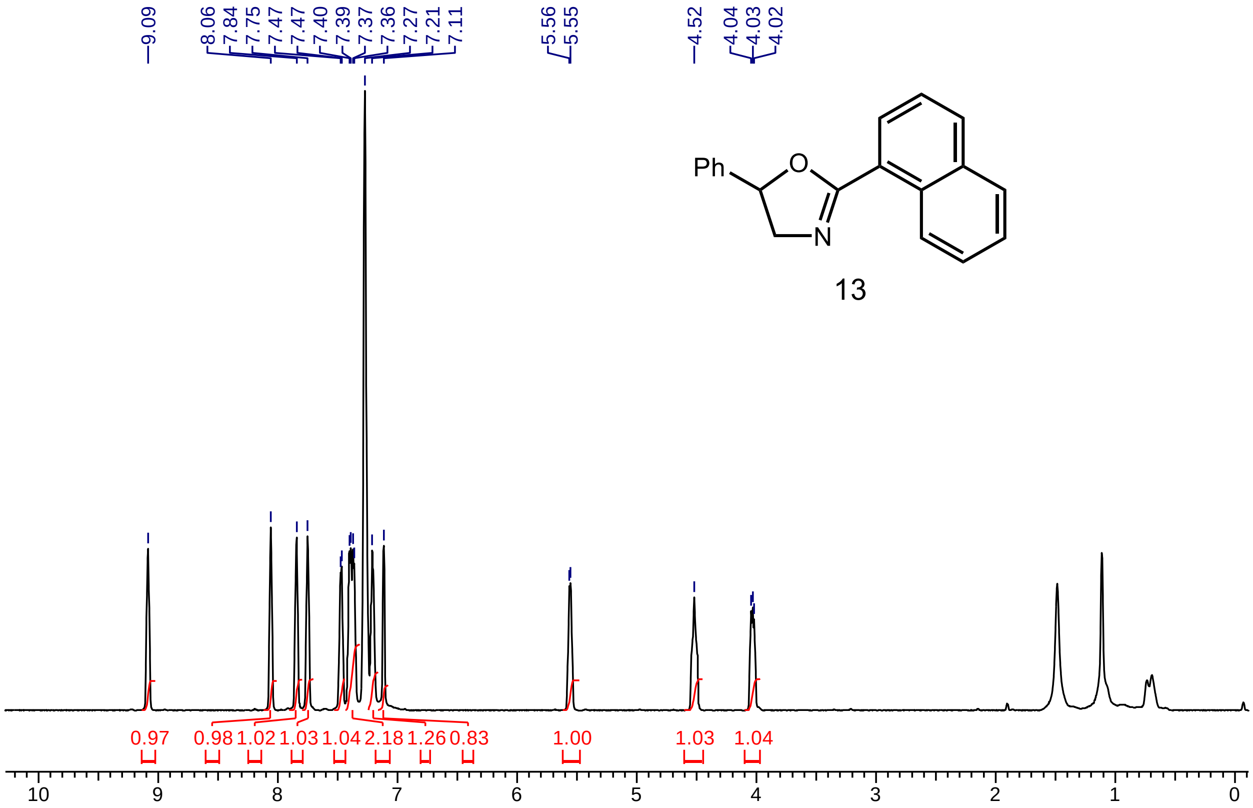
9.09
8.06
7.84
7.75
7.47
7.47
7.40
7.39
7.37
7.36
7.27
7.21
7.11
5.56
5.55
4.52
4.04
4.03
4.02
0.97
0.98
1.02
1.03
1.04
2.18
1.26
0.83
1.00
1.03
1.04
10
9
8
7
6
5
4
3
2
1
0
Ph
O
N

13

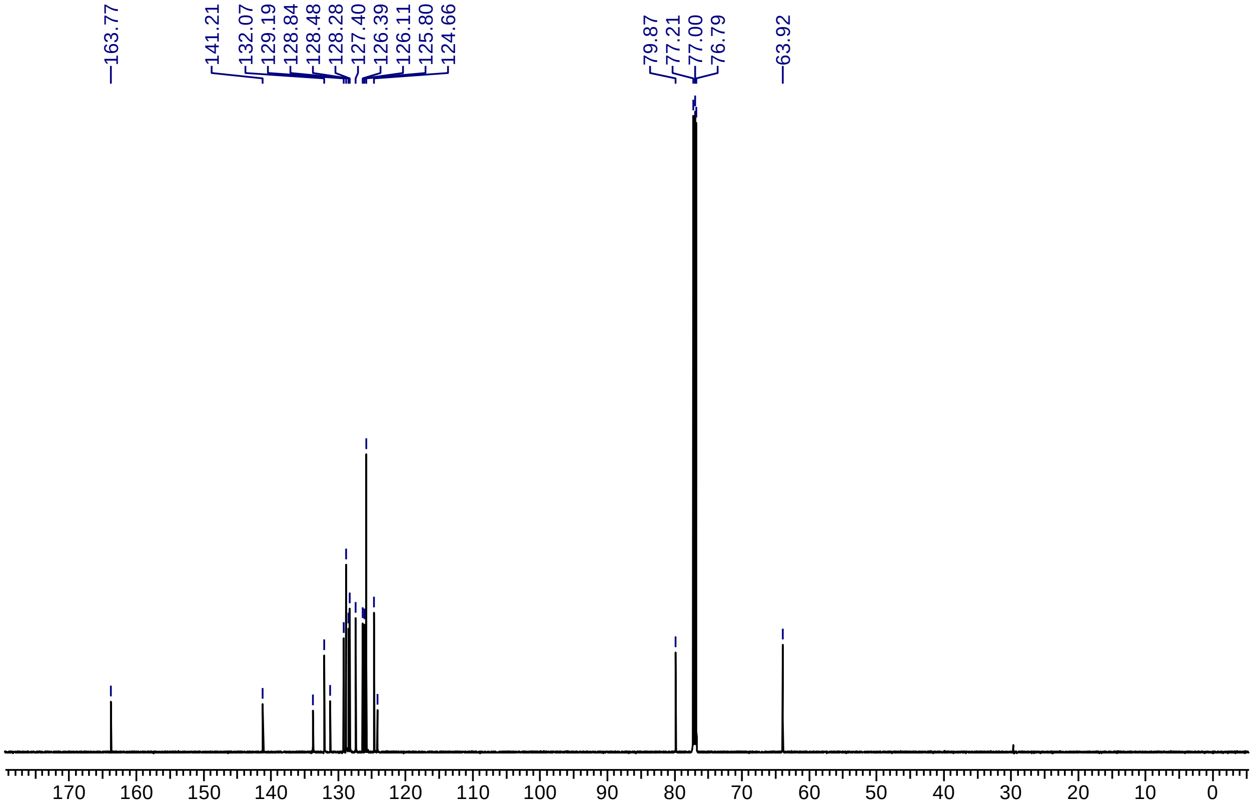
163.77
141.21
132.07
129.19
128.84
128.48
128.28
127.40
126.39
126.11
125.80
124.66
79.87
77.21
77.00
76.79
63.92
170
160
150
140
130
120
110
100
90
80
70
60
50
40
30
20
10
0

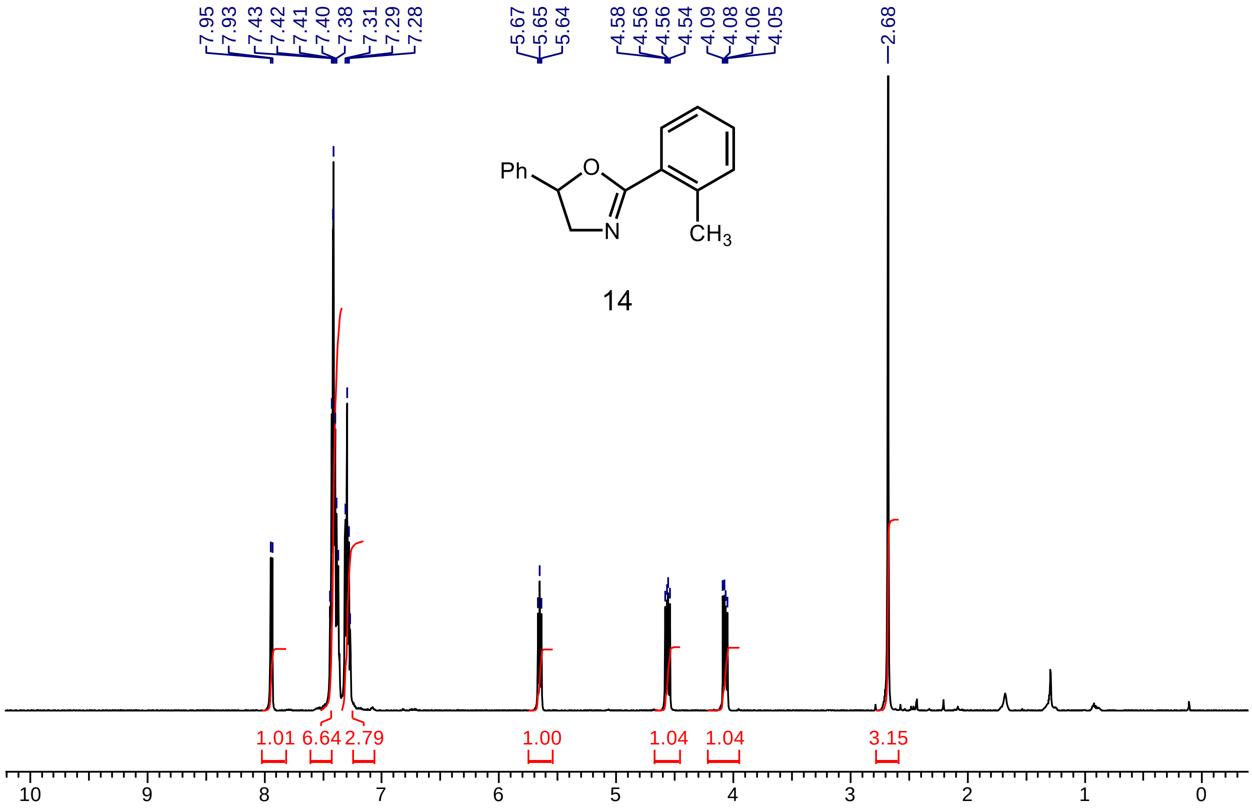
7.95
7.93
7.43
7.42
7.41
7.40
7.38
7.31
7.29
7.28
5.67
5.65
5.64
4.58
4.56
4.56
4.54
4.09
4.08
4.06
4.05
2.68
Ph
O
N
CH3
14
1.01
6.64
2.79
1.00
1.04
1.04
3.15

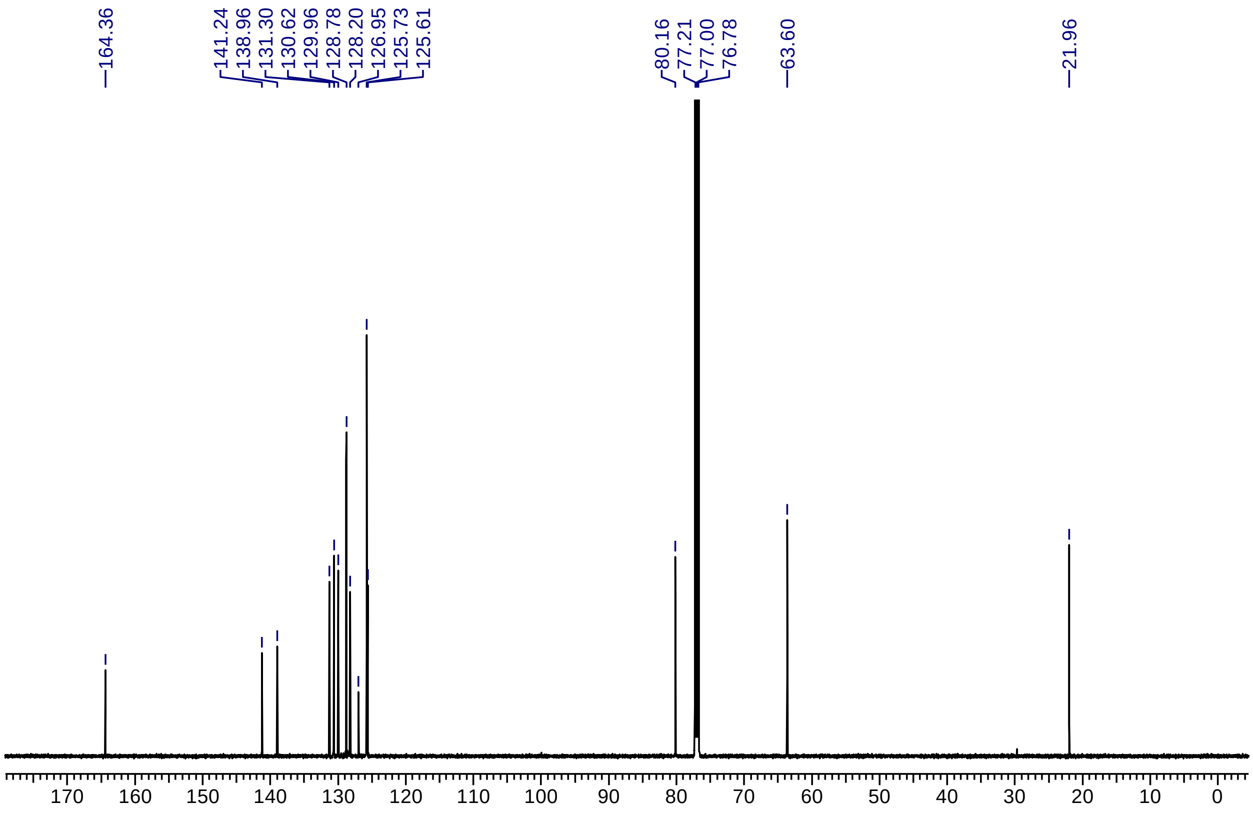
164.36
141.24
138.96
131.30
130.62
129.96
128.78
128.20
126.95
125.73
125.61
80.16
77.21
77.00
76.78
63.60
21.96

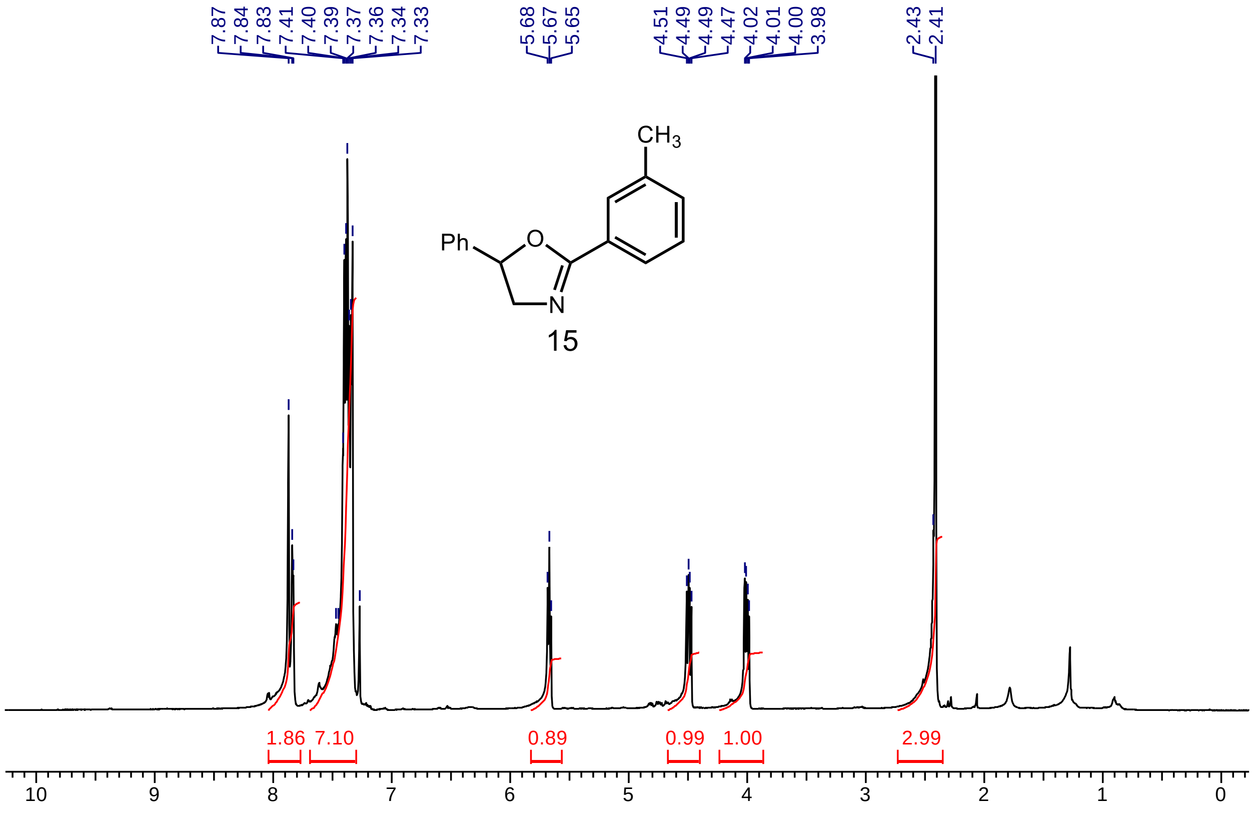
7.87
7.84
7.83
7.41
7.40
7.39
7.37
7.36
7.34
7.33
5.68
5.67
5.65
4.51
4.49
4.49
4.47
4.02
4.01
4.00
3.98
2.43
2.41
CH3
Ph
O
N
15
1.86
7.10
0.89
0.99
1.00
2.99
10
9
8
7
6
5
4
3
2
1
0

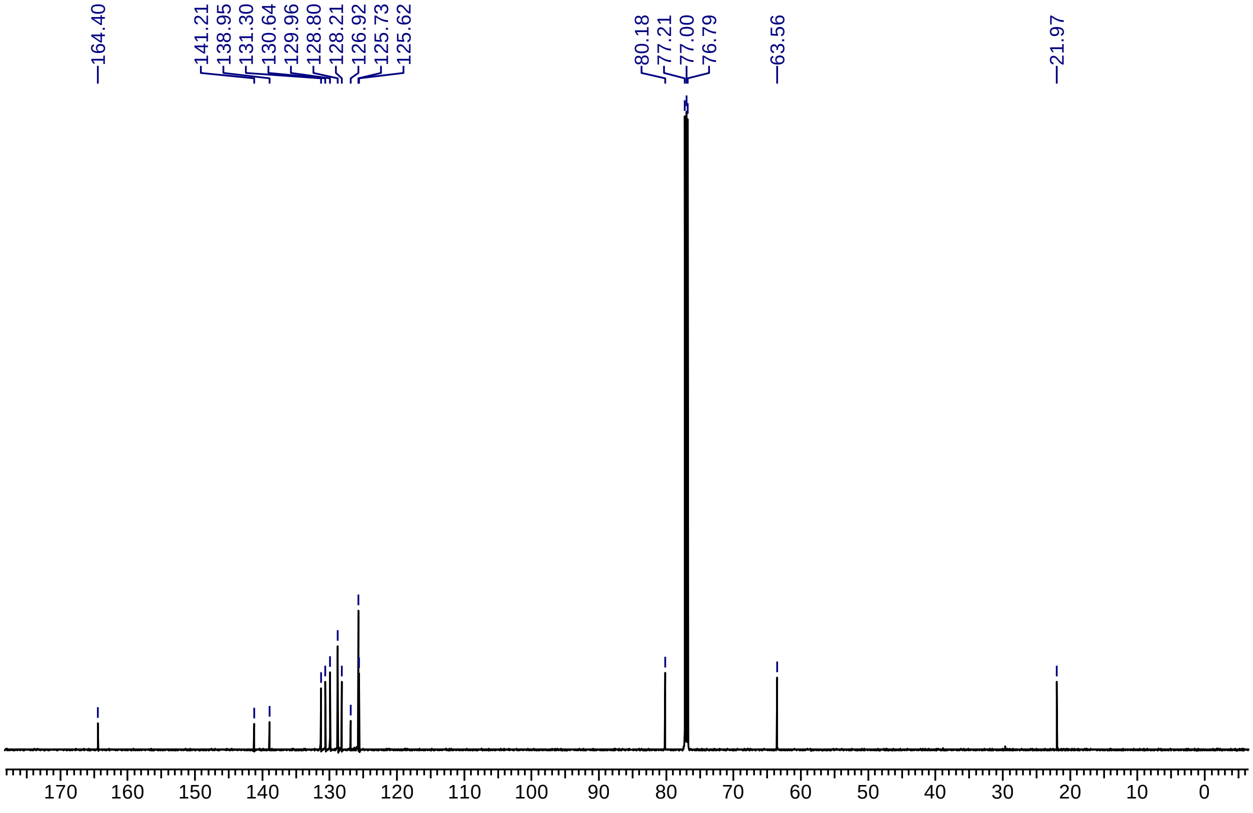
164.40
141.21
138.95
131.30
130.64
129.96
128.80
128.21
126.92
125.73
125.62
80.18
77.21
77.00
76.79
63.56
21.97
170
160
150
140
130
120
110
100
90
80
70
60
50
40
30
20
10
0

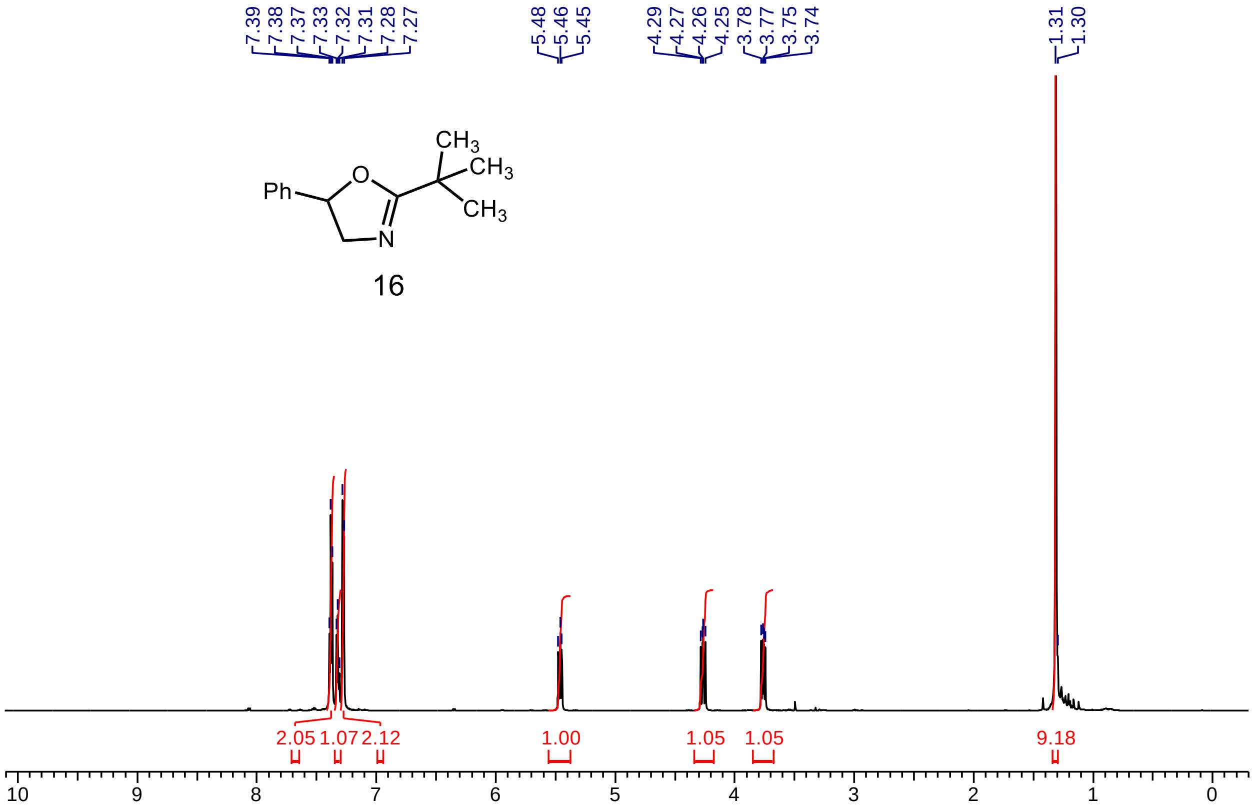
7.39 7.38 7.37 7.33 7.32 7.31 7.28 7.27
5.48 5.46 5.45
4.29 4.27 4.26 4.25 3.78 3.77 3.75 3.74
1.31 1.30
Ph
O
N
CH3
CH3
CH3
16
2.05 1.07 2.12
1.00
1.05
1.05
9.18
10 9 8 7 6 5 4 3 2 1 0

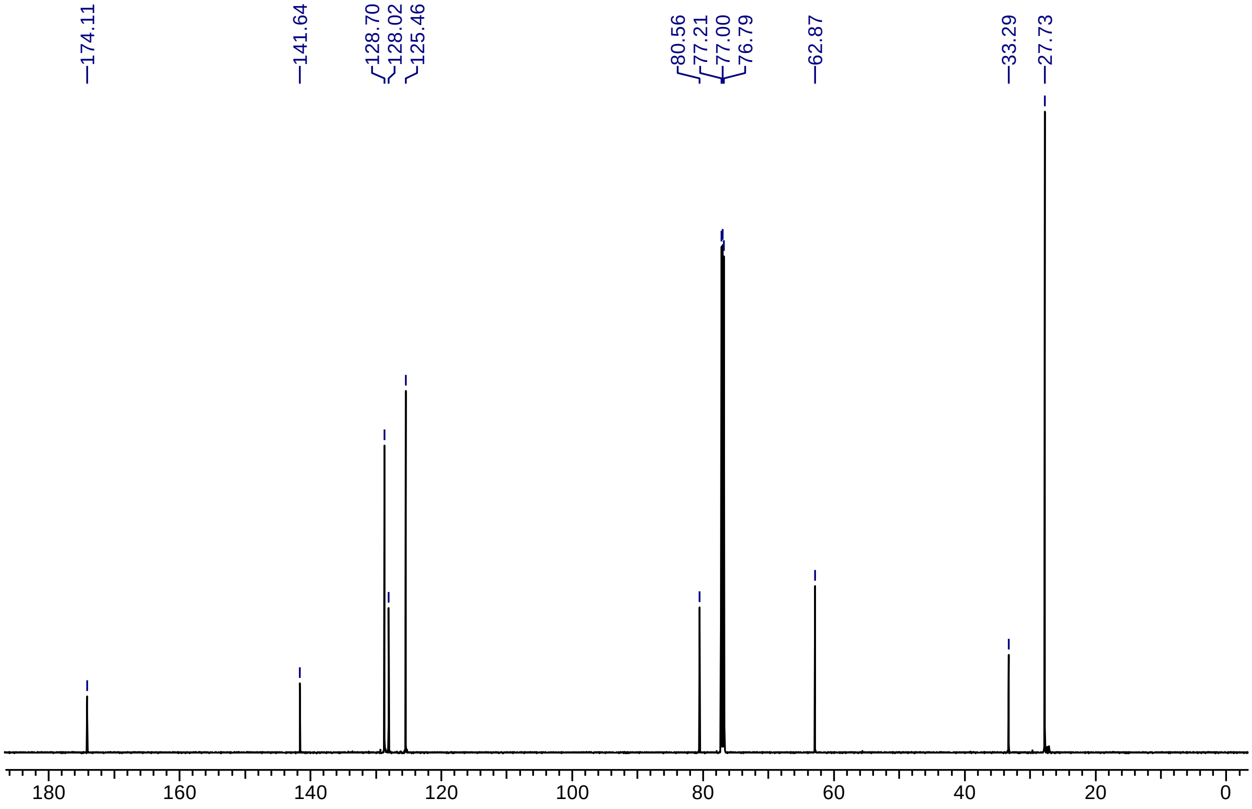
174.11
141.64
128.70 128.02 125.46
80.56 77.21 77.00 76.79
62.87
33.29 27.73
180 160 140 120 100 80 60 40 20 0

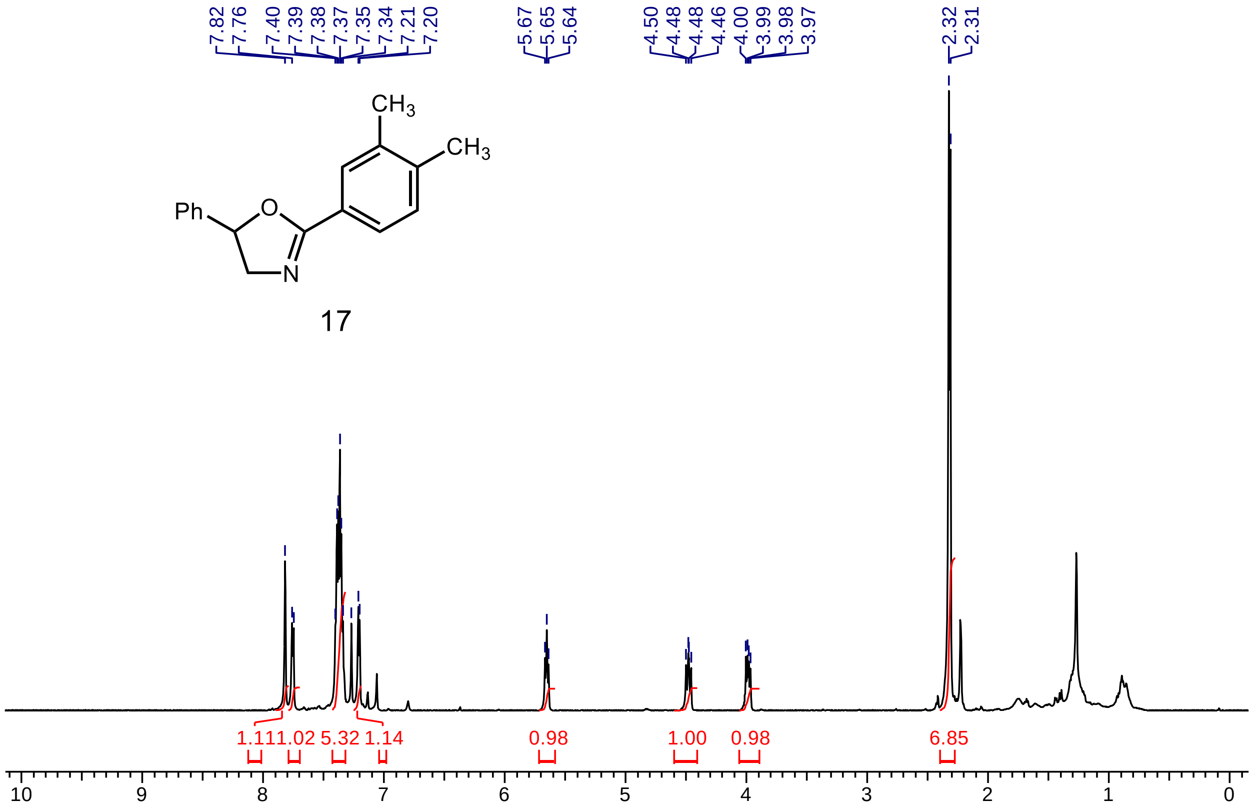

CH3
CH3
Ph
O
N
7.82
7.76
7.40
7.39
7.38
7.37
7.35
7.34
7.21
7.20
5.67
5.65
5.64
4.50
4.48
4.48
4.46
4.00
3.99
3.98
3.97
2.32
2.31
1.11
1.02
5.32
1.14
0.98
1.00
0.98
6.85
10
9
8
7
6
5
4
3
2
1
0

17

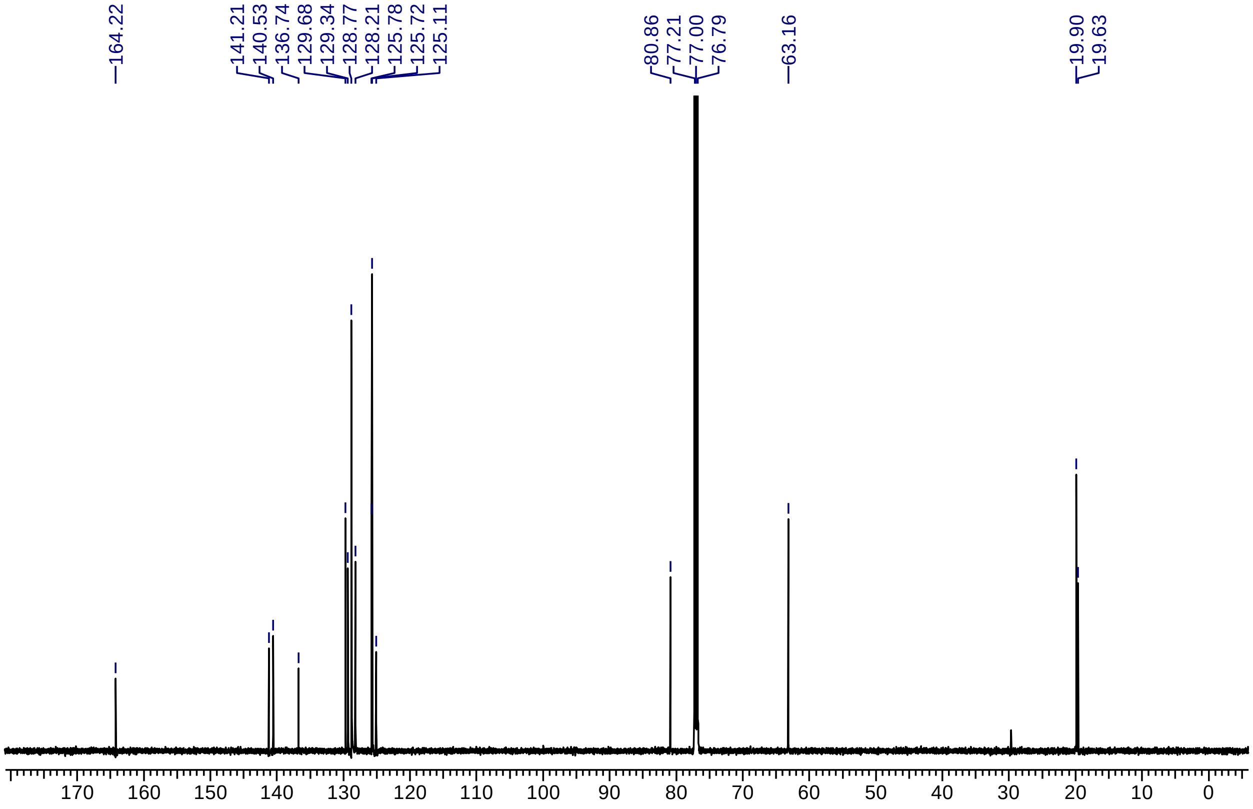

164.22
141.21
140.53
136.74
129.68
129.34
128.77
128.21
125.78
125.72
125.11
80.86
77.21
77.00
76.79
63.16
19.90
19.63
170
160
150
140
130
120
110
100
90
80
70
60
50
40
30
20
10
0

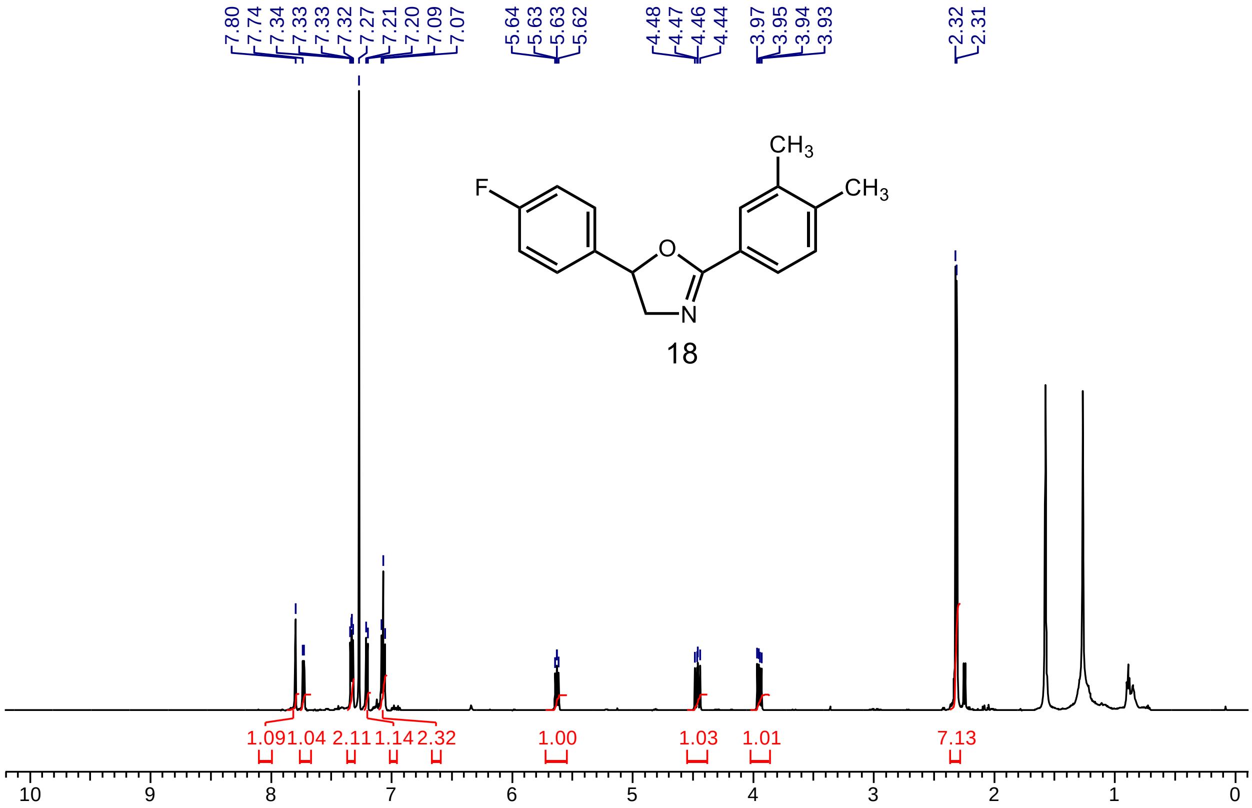

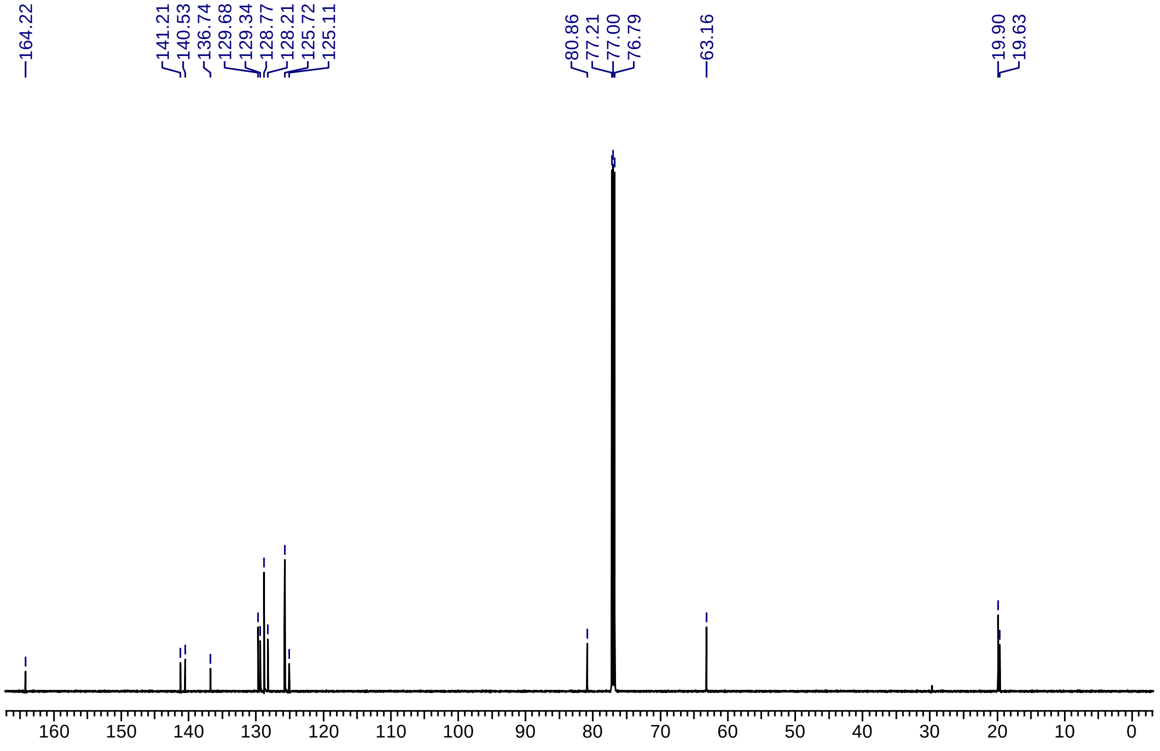

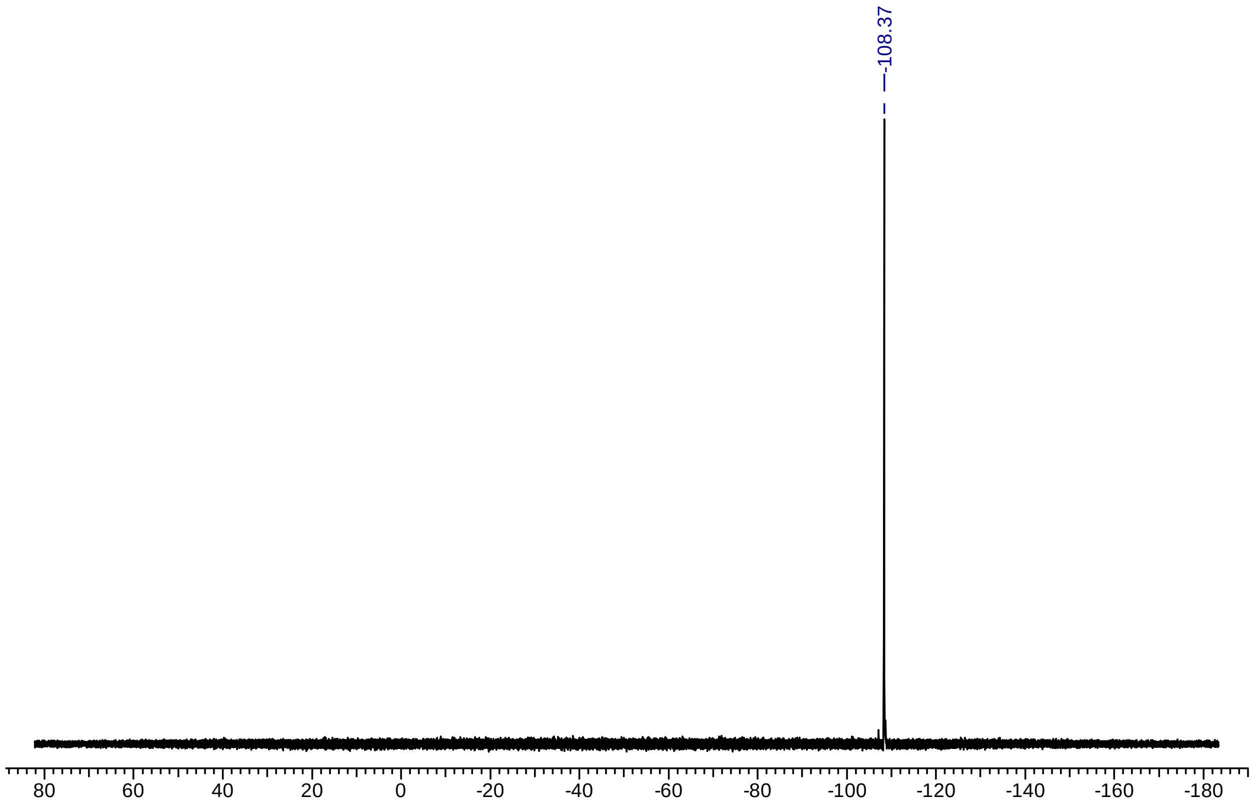
-108.37
80
60
40
20
0
-20
-40
-60
-80
-100
-120
-140
-160
-180

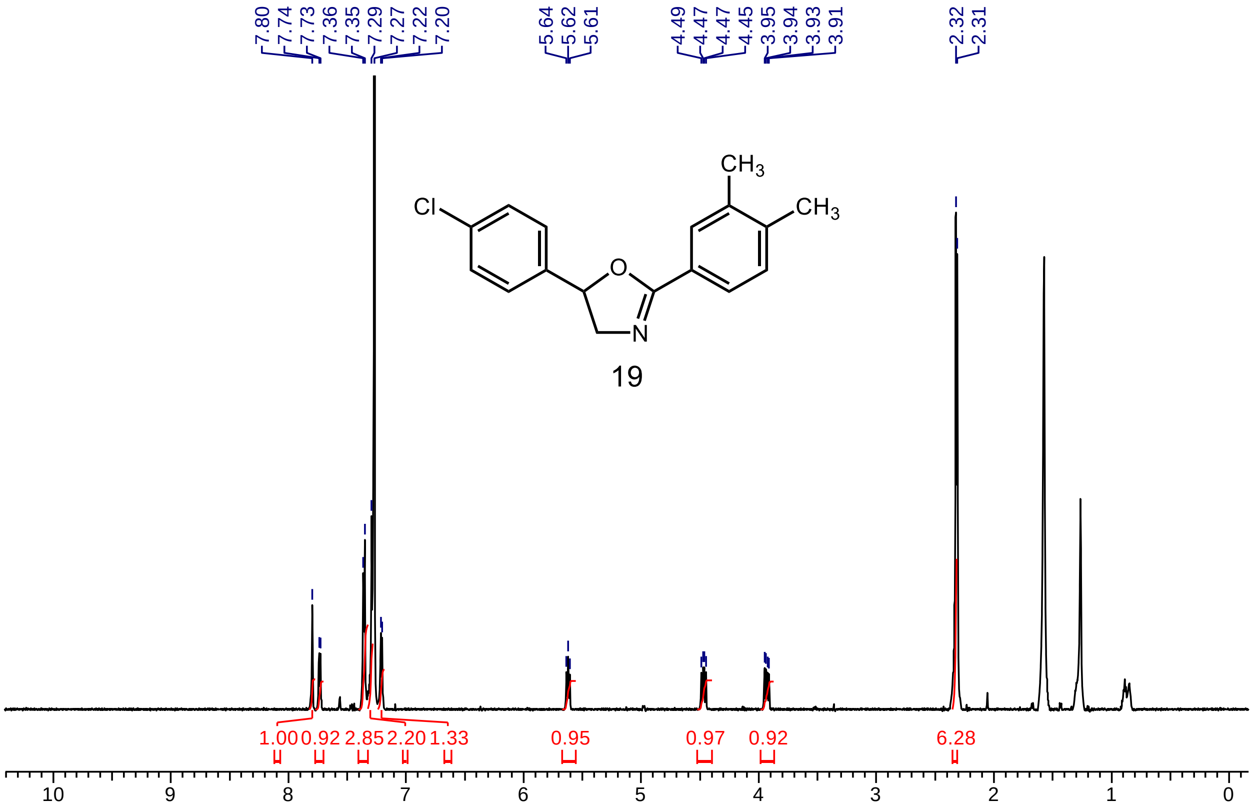
7.80
7.74
7.73
7.36
7.35
7.29
7.27
7.22
7.20
5.64
5.62
5.61
4.49
4.47
4.47
4.45
3.95
3.94
3.93
3.91
2.32
2.31
CH3
Cl
CH3
O
N
19
1.00
0.92
2.85
2.20
1.33
0.95
0.97
0.92
6.28
10
9
8
7
6
5
4
3
2
1
0

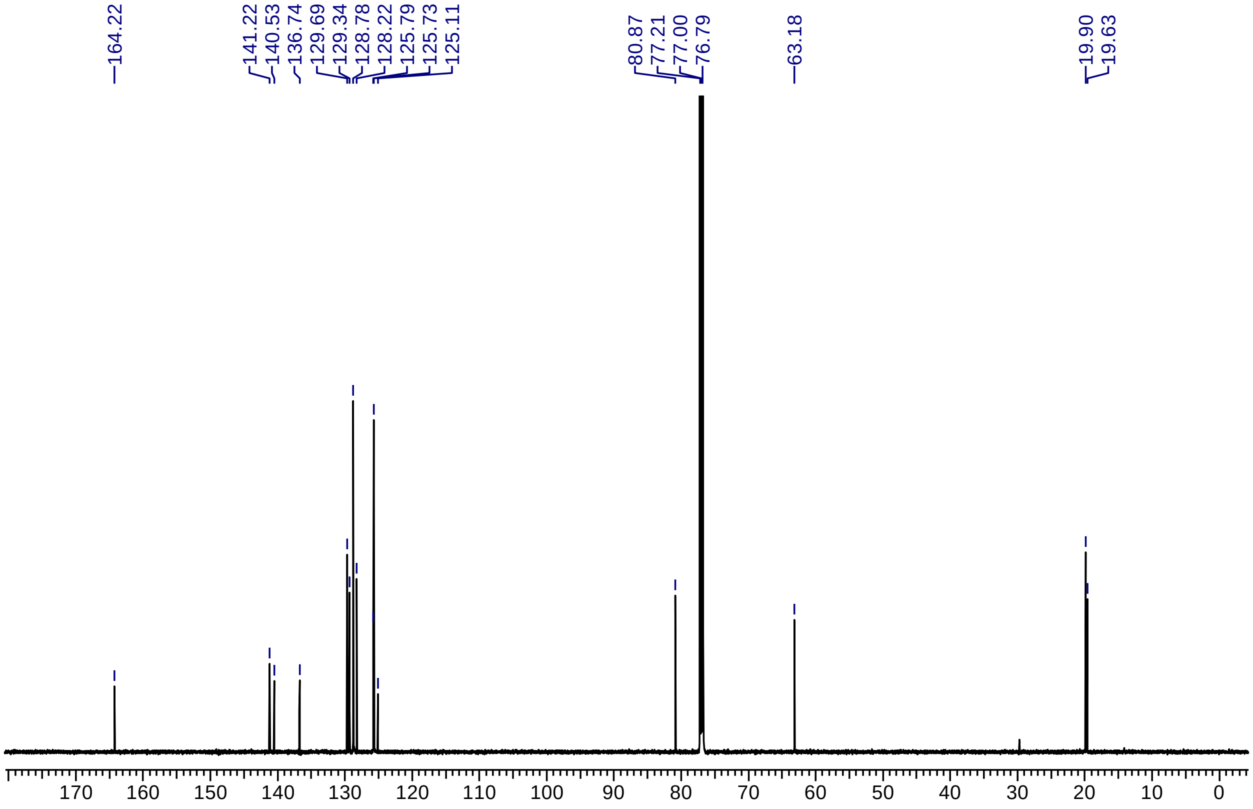
164.22
141.22
140.53
136.74
129.69
129.34
128.78
128.22
125.79
125.73
125.11
80.87
77.21
77.00
76.79
63.18
19.90
19.63
170
160
150
140
130
120
110
100
90
80
70
60
50
40
30
20
10
0

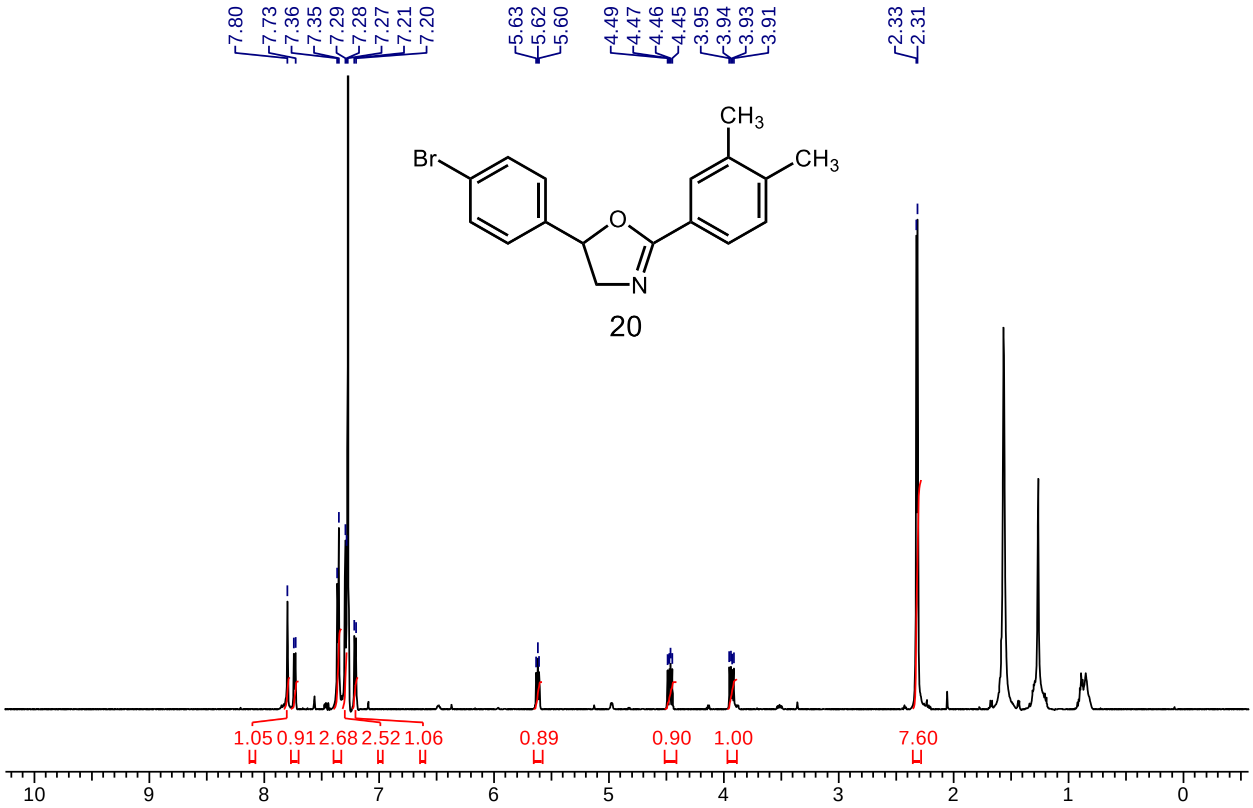
7.80 7.73 7.36 7.35 7.29 7.28 7.27 7.21 7.20
5.63 5.62 5.60
4.49 4.47 4.46 4.45 3.95 3.94 3.93 3.91
2.33 2.31
Br
O
N
CH_3
CH_3
20
1.05 0.91 2.68 2.52 1.06
0.89
0.90 1.00
7.60
10 9 8 7 6 5 4 3 2 1 0

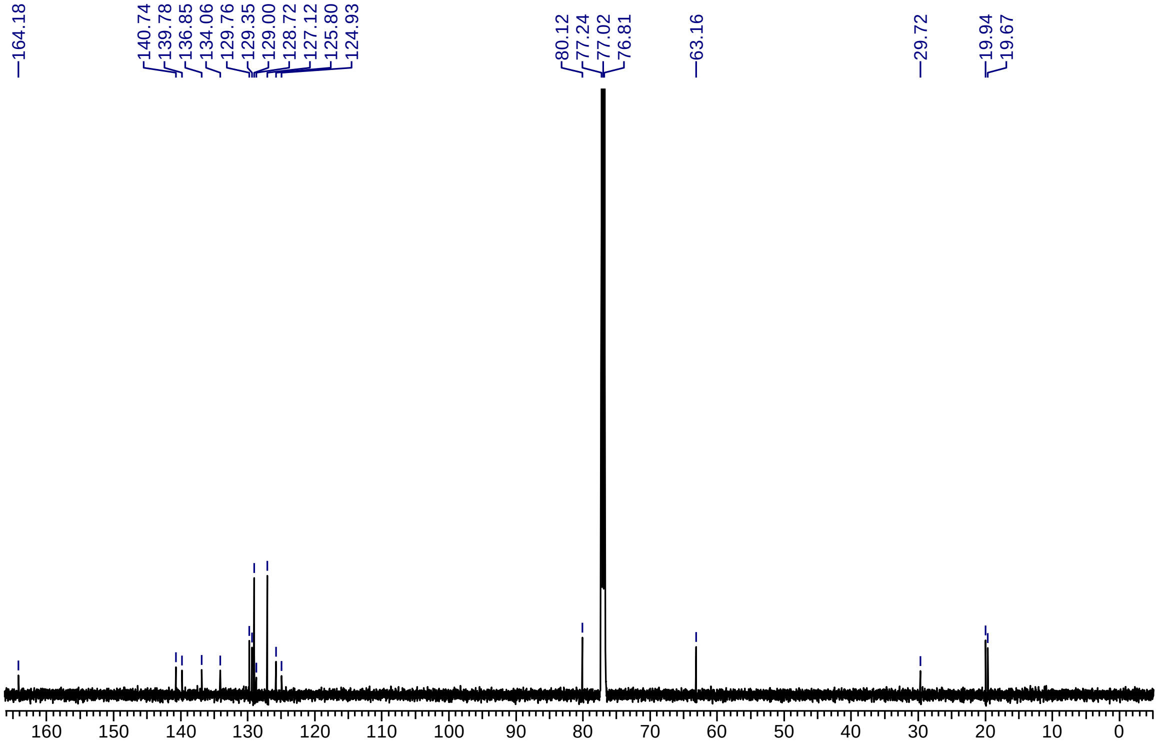
164.18
140.74 139.78 136.85 134.06 129.76 129.35 129.00 128.72 127.12 125.80 124.93
80.12 77.24 77.02 76.81
63.16
29.72
19.94 19.67
160 150 140 130 120 110 100 90 80 70 60 50 40 30 20 10 0

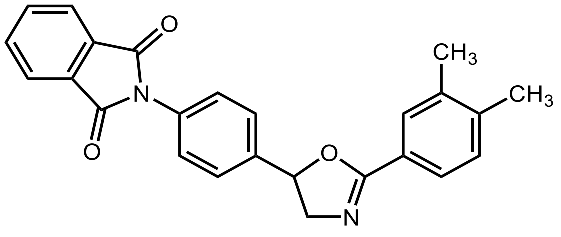

21

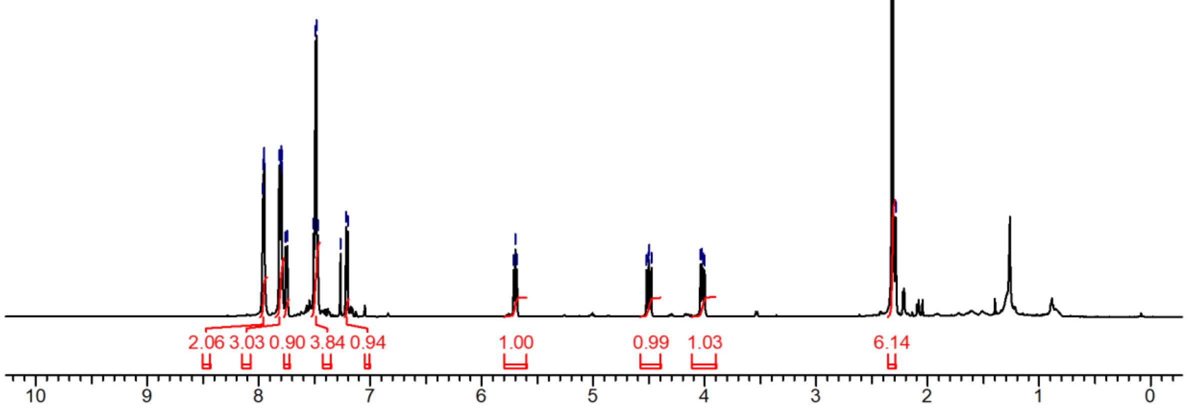

7.96
7.96
7.95
7.82
7.81
7.80
7.80
7.79
7.49
7.48
7.22
5.72
5.70
5.69
4.52
4.51
4.50
4.48
4.04
4.03
4.01
4.00
2.32
2.31
2.29
2.06
3.03
0.90
3.84
0.94
1.00
0.99
1.03
6.14
10
9
8
7
6
5
4
3
2
1
0

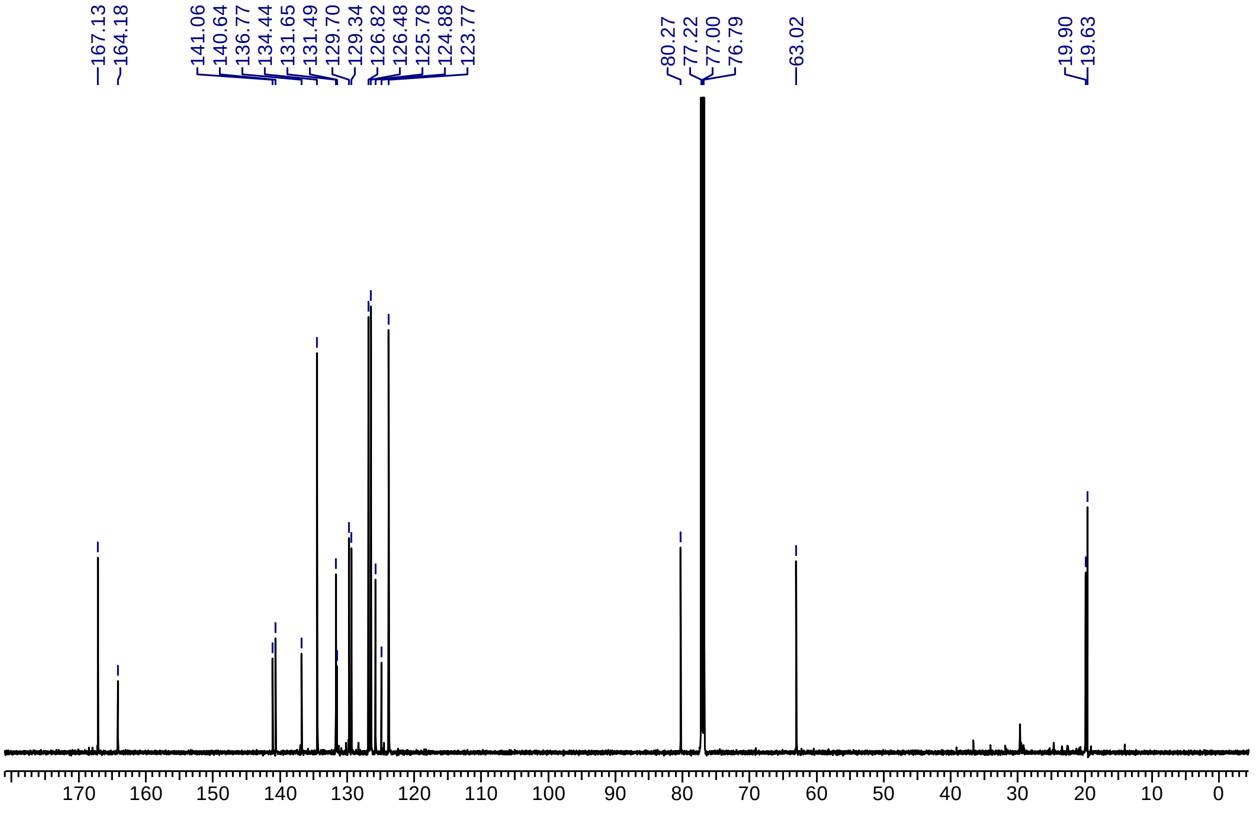

167.13
164.18
141.06
140.64
136.77
134.44
131.65
131.49
129.70
129.34
126.82
126.48
125.78
124.88
123.77
80.27
77.22
77.00
76.79
63.02
19.90
19.63
170
160
150
140
130
120
110
100
90
80
70
60
50
40
30
20
10
0

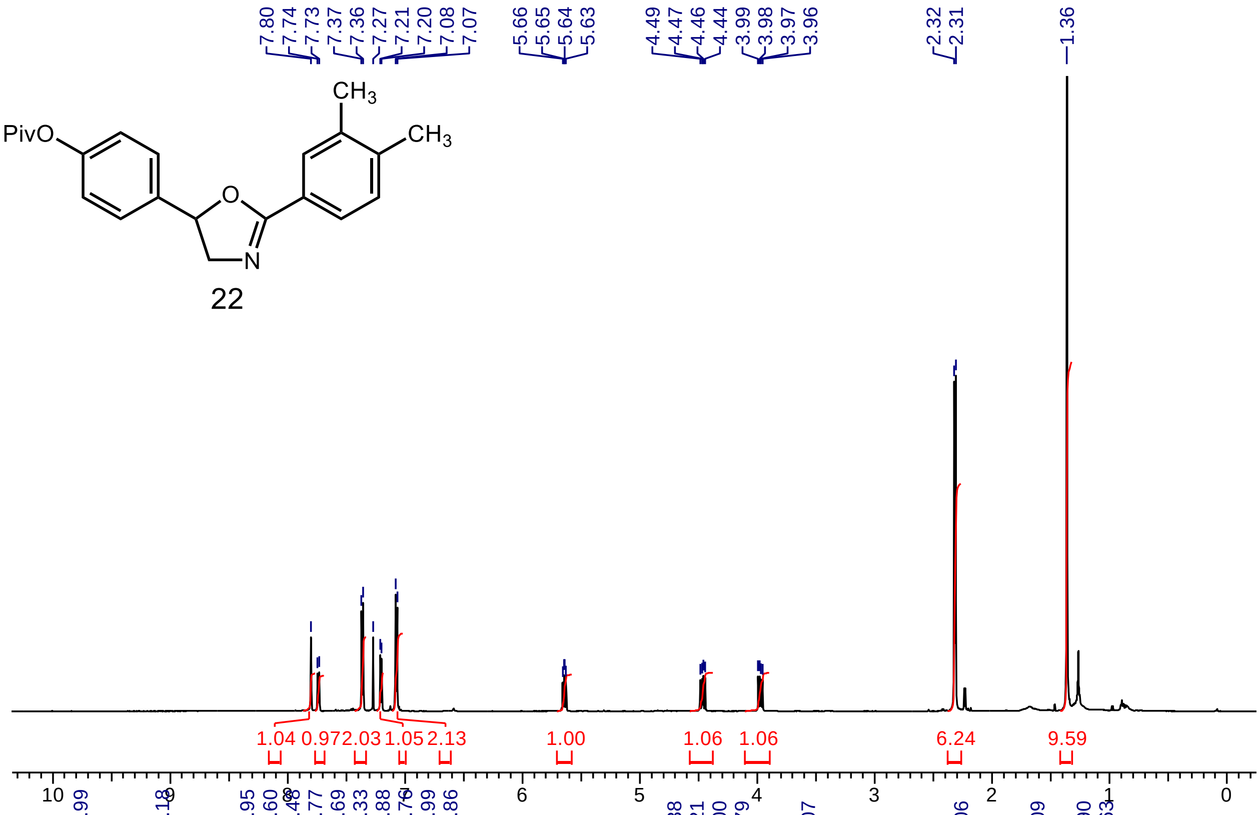
PivO
O
N
CH3
CH3
22
7.80
7.74
7.73
7.37
7.36
7.27
7.21
7.20
7.08
7.07
5.66
5.65
5.64
5.63
4.49
4.47
4.46
4.44
3.99
3.98
3.97
3.96
2.32
2.31
1.36
1.04
0.97
2.03
1.05
2.13
1.00
1.06
1.06
6.24
9.59
10
9
8
7
6
5
4
3
2
1
0

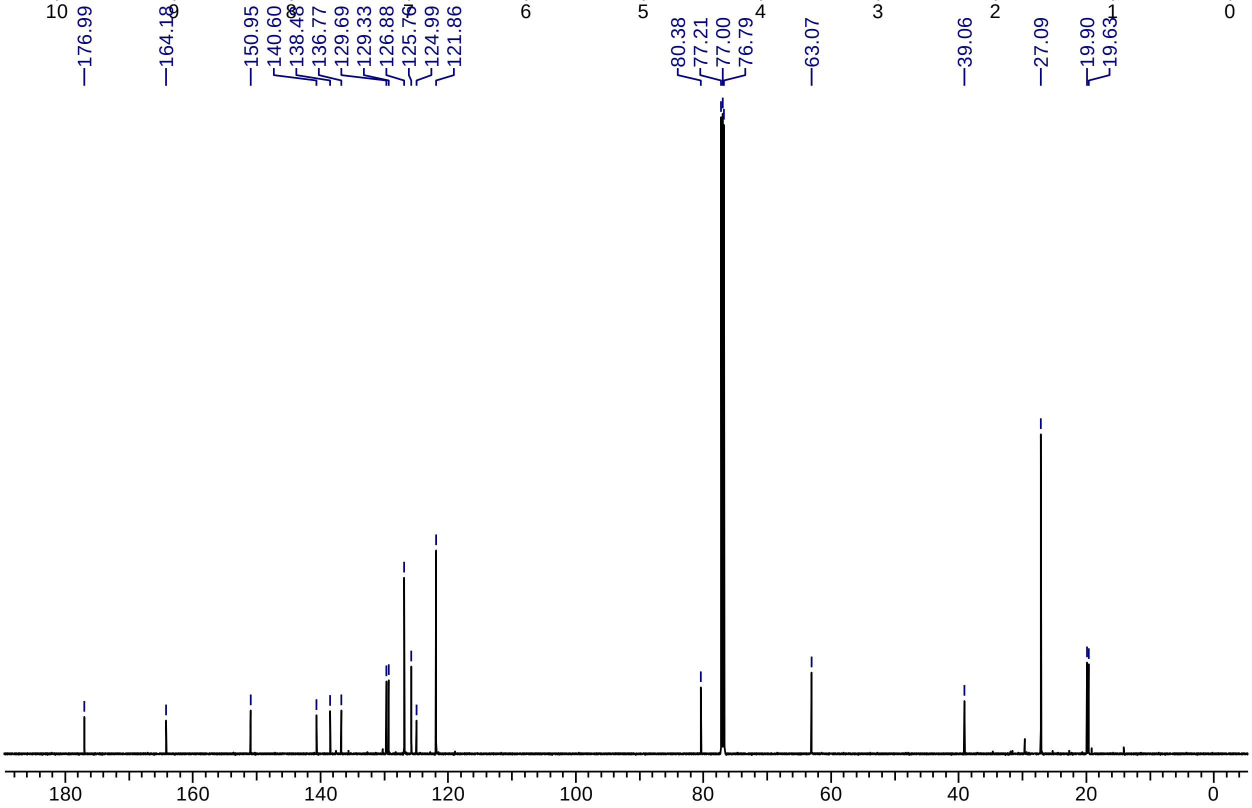
176.99
164.18
150.95
140.60
138.48
136.77
129.69
129.33
126.88
125.70
124.99
121.86
80.38
77.21
77.00
76.79
63.07
39.06
27.09
19.90
19.63
180
160
140
120
100
80
60
40
20
0

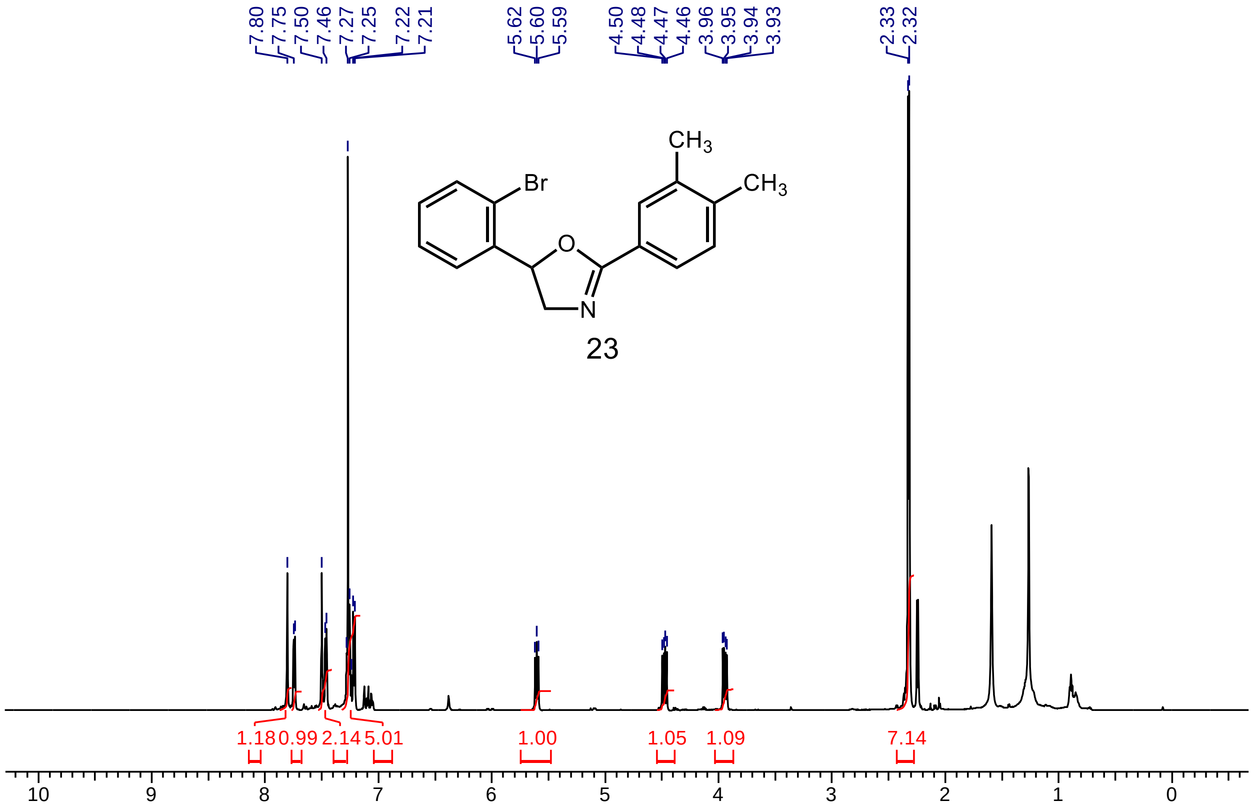
7.80
7.75
7.50
7.46
7.27
7.25
7.22
7.21
5.62
5.60
5.59
4.50
4.48
4.47
4.46
3.96
3.95
3.94
3.93
2.33
2.32
Br
O
N
CH_3
CH_3
23
1.18
0.99
2.14
5.01
1.00
1.05
1.09
7.14
10
9
8
7
6
5
4
3
2
1
0

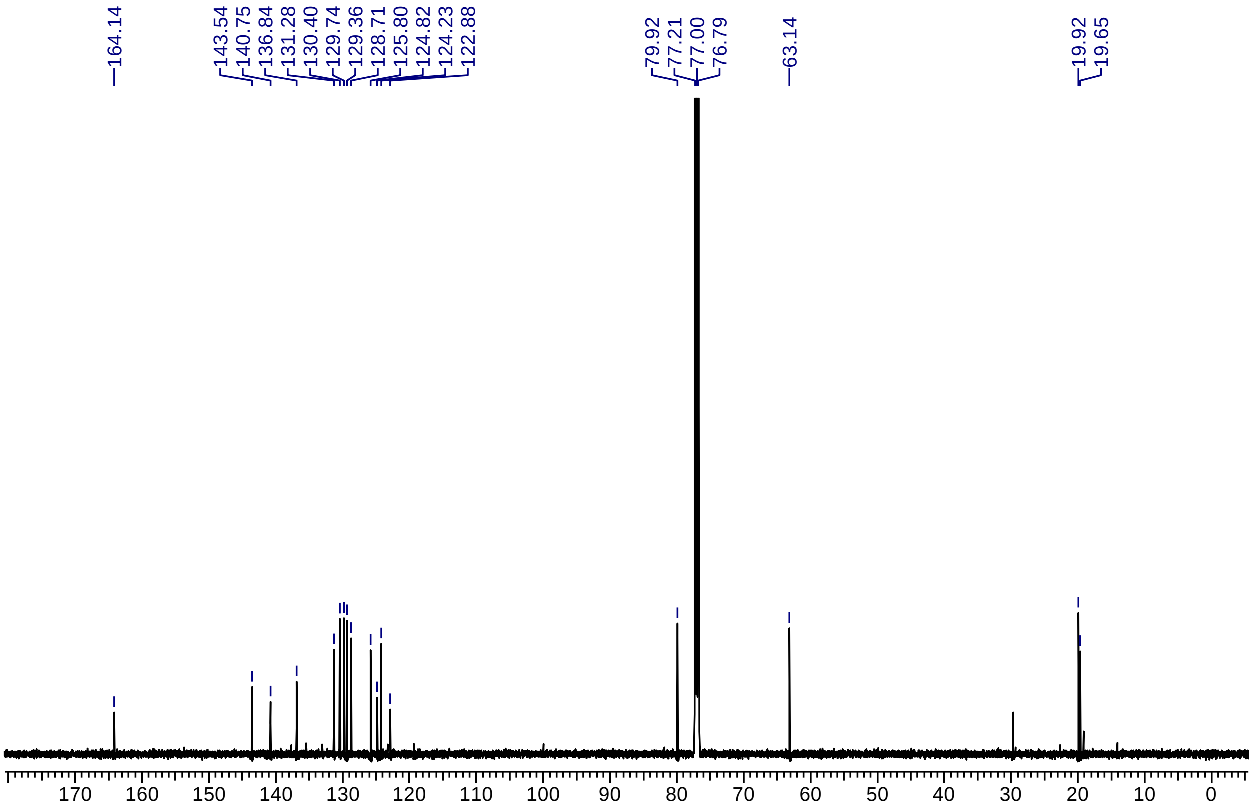
164.14
143.54
140.75
136.84
131.28
130.40
129.74
129.36
128.71
125.80
124.82
124.23
122.88
79.92
77.21
77.00
76.79
63.14
19.92
19.65
170
160
150
140
130
120
110
100
90
80
70
60
50
40
30
20
10
0

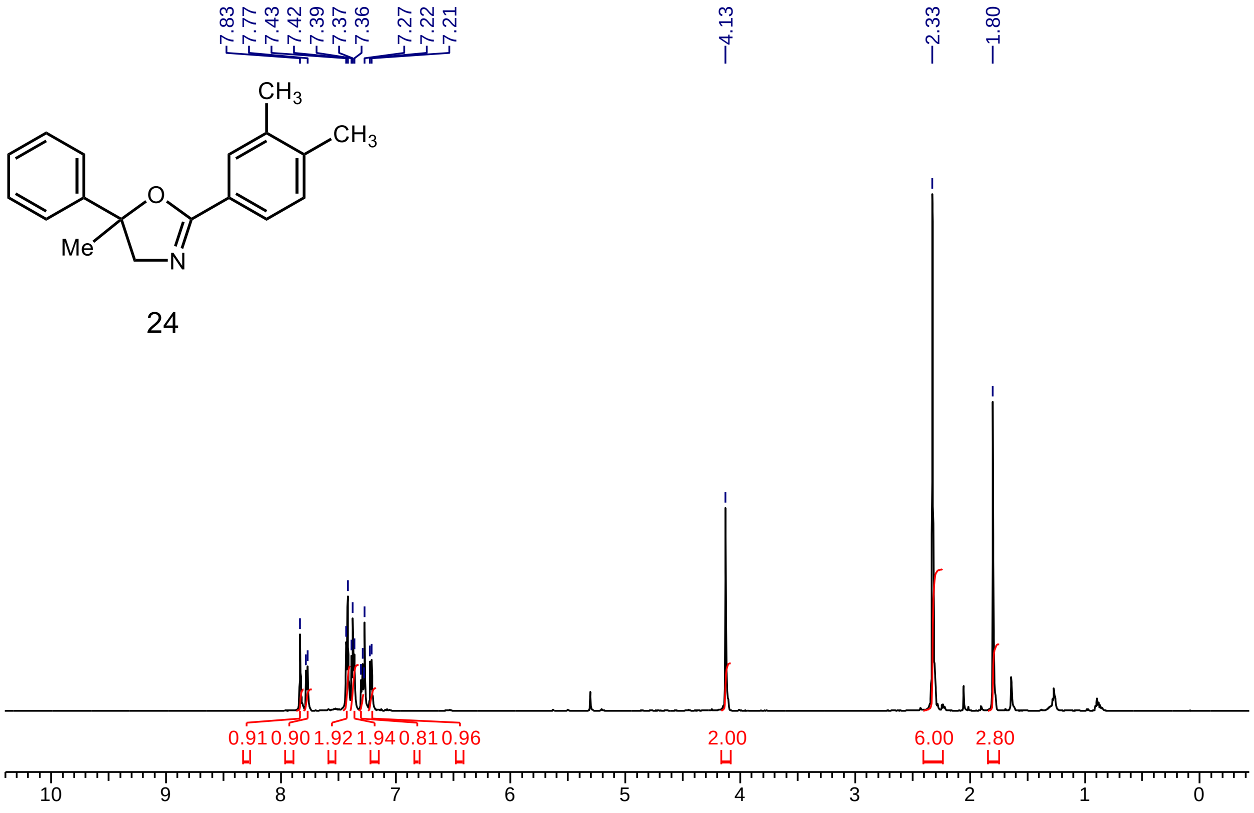
7.83
7.77
7.43
7.42
7.39
7.37
7.36
7.27
7.22
7.21
4.13
2.33
1.80
CH3
CH3
O
N
Me
24
0.91
0.90
1.92
1.94
0.81
0.96
2.00
6.00
2.80
10
9
8
7
6
5
4
3
2
1
0

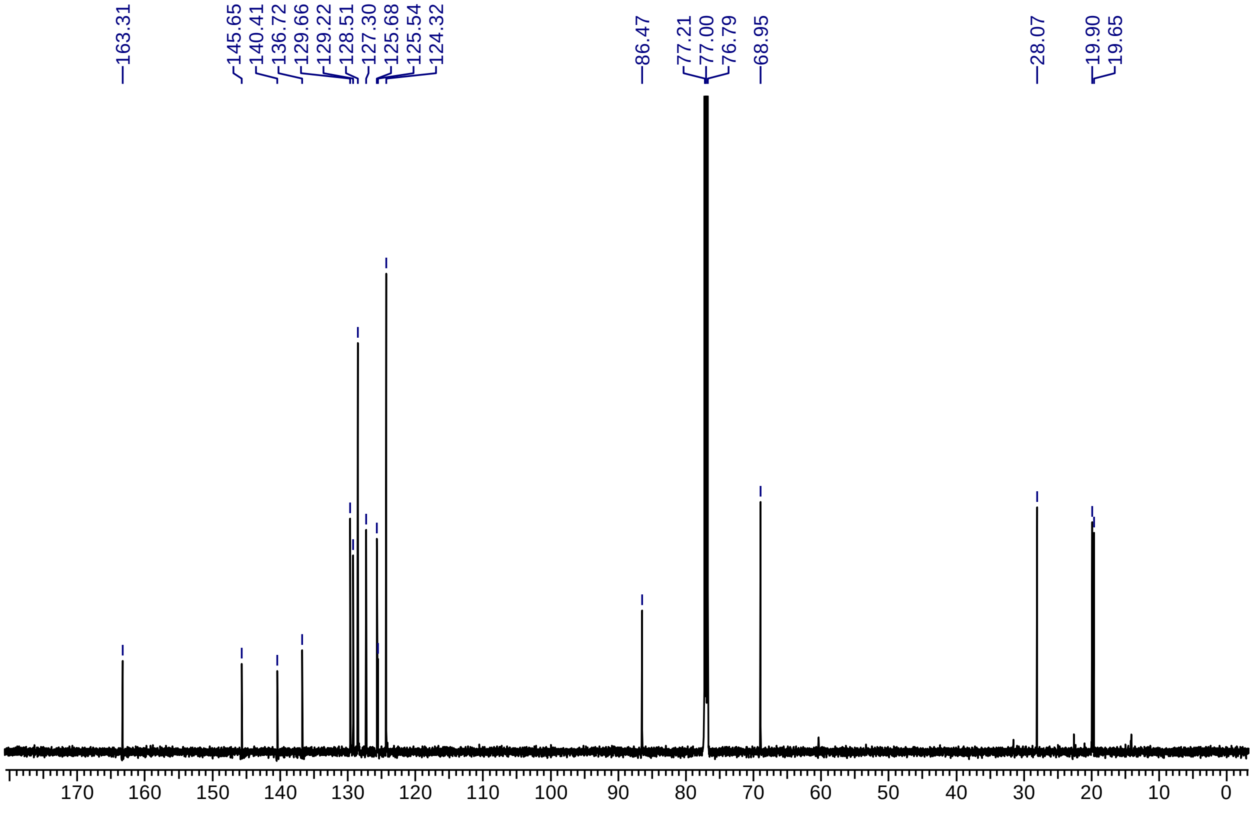
163.31
145.65
140.41
136.72
129.66
129.22
128.51
127.30
125.68
125.54
124.32
86.47
77.21
77.00
76.79
68.95
28.07
19.90
19.65
170
160
150
140
130
120
110
100
90
80
70
60
50
40
30
20
10
0

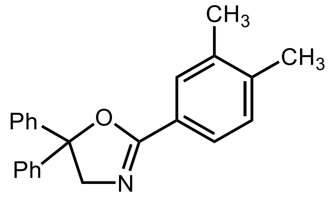
CH3
CH3
Ph
Ph
O
N

25

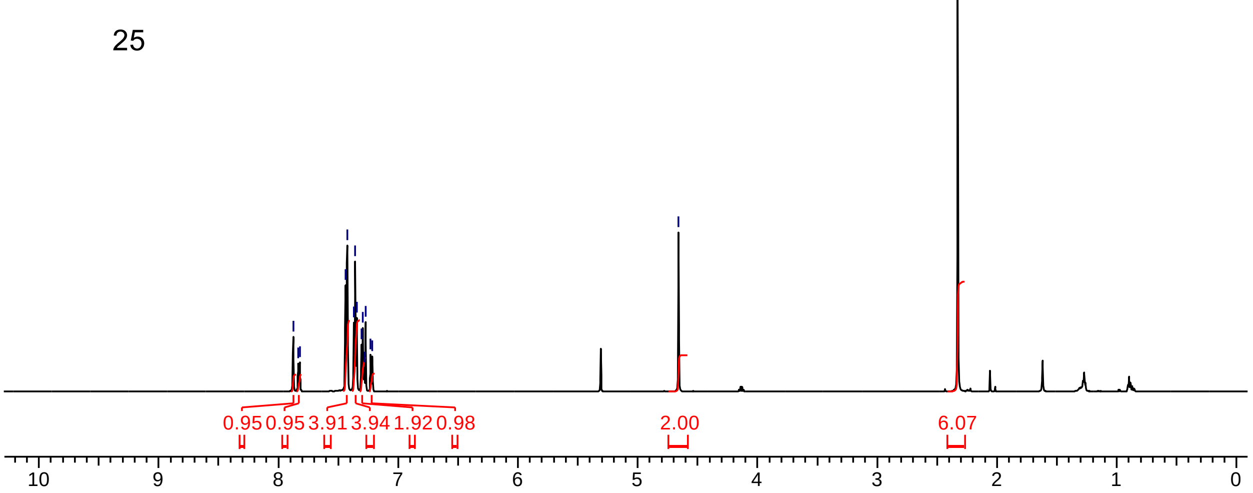
7.88
7.82
7.44
7.43
7.37
7.36
7.35
7.29
7.27
4.66
2.33
0.95
0.95
3.91
3.94
1.92
0.98
2.00
6.07
10 9 8 7 6 5 4 3 2 1 0

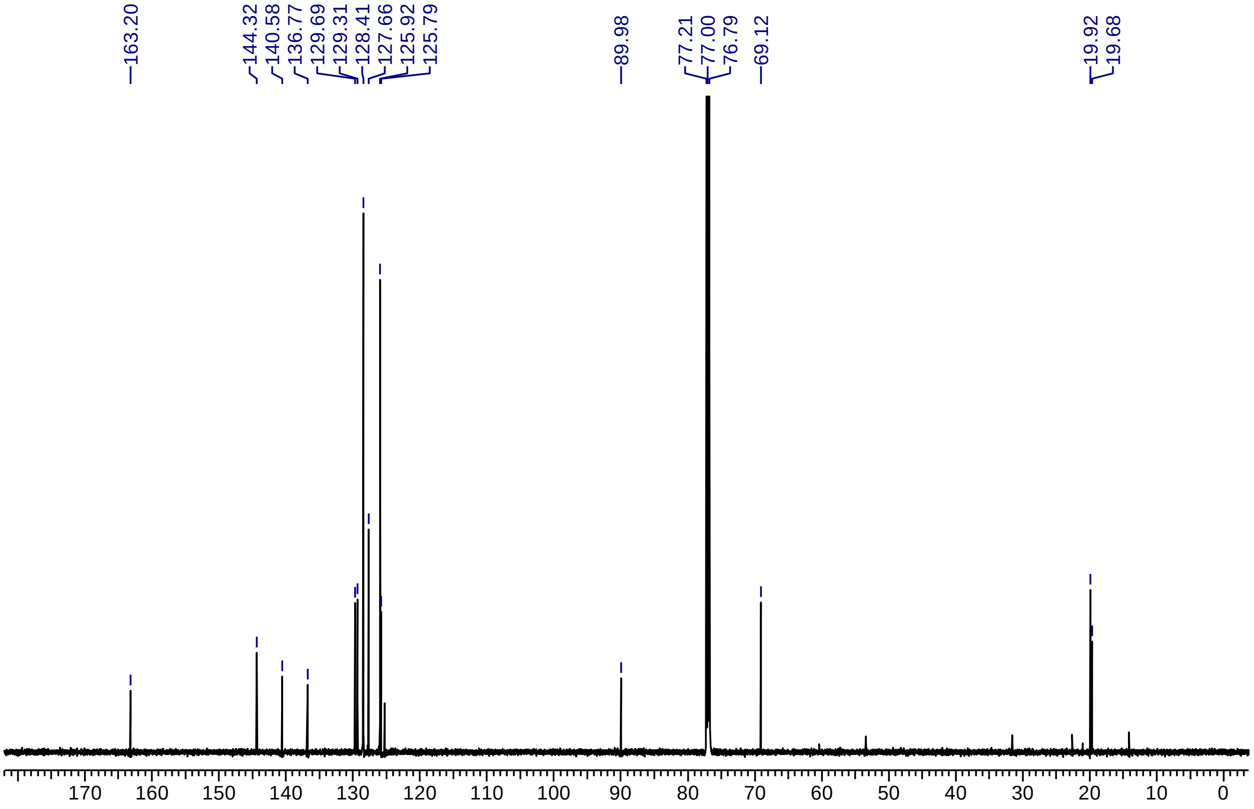
163.20
144.32
140.58
136.77
129.69
129.31
128.41
127.66
125.92
125.79
89.98
77.21
77.00
76.79
69.12
19.92
19.68
170 160 150 140 130 120 110 100 90 80 70 60 50 40 30 20 10 0
